# Silvaticusins A–D: *ent*-kaurane diterpenoids and a cyclobutane-containing *ent*-kaurane dimer from *Isodon silvaticus*

**DOI:** 10.1007/s13659-024-00465-9

**Published:** 2024-08-15

**Authors:** Qi-Xiu Hai, Kun Hu, Su-Ping Chen, Yang-Yang Fu, Xiao-Nian Li, Han-Dong Sun, Hong-Ping He, Pema-Tenzin Puno

**Affiliations:** 1 College of Chinese Materia Medica and Yunnan Key Laboratory of Southern Medicinal Utilization, Yunnan University of Chinese Medicine, Kunming, 650500 People’s Republic of China; 2 Key Laboratory of Phytochemistry and Natural Medicines, Kunming Institute of Botany, Chinese Academy of Sciences, Kunming, 650201 People’s Republic of China

**Keywords:** *Isodon silvaticus*, *ent*-Kaurane diterpenoid, *ent*-Kaurane dimer, Cyclobutane moiety, Cytotoxicity

## Abstract

Three new *ent*-kaurane diterpenoids, silvaticusins A–C (**1**–**3**), along with a new *ent*-kaurane dimer silvaticusin D (**4**) were isolated from the aerial parts of *Isodon silvaticus*. The structures of these new compounds were established mainly by comprehensive analysis of their NMR and MS data. The absolute configuration of compounds **1** and **4** were determined using a single-crystal X-ray diffraction and computational methods, respectively. Compounds **2** and **3** were found to exhibit remarkable cytotoxic effects against five human tumor cell lines (HL-60, A-549, SMMC-7721, MDA-MB-231, and SW-480), with IC_50_ values spanning from 1.27 ± 0.08 to 7.52 ± 0.33 μM.

**Graphical Abstract:**

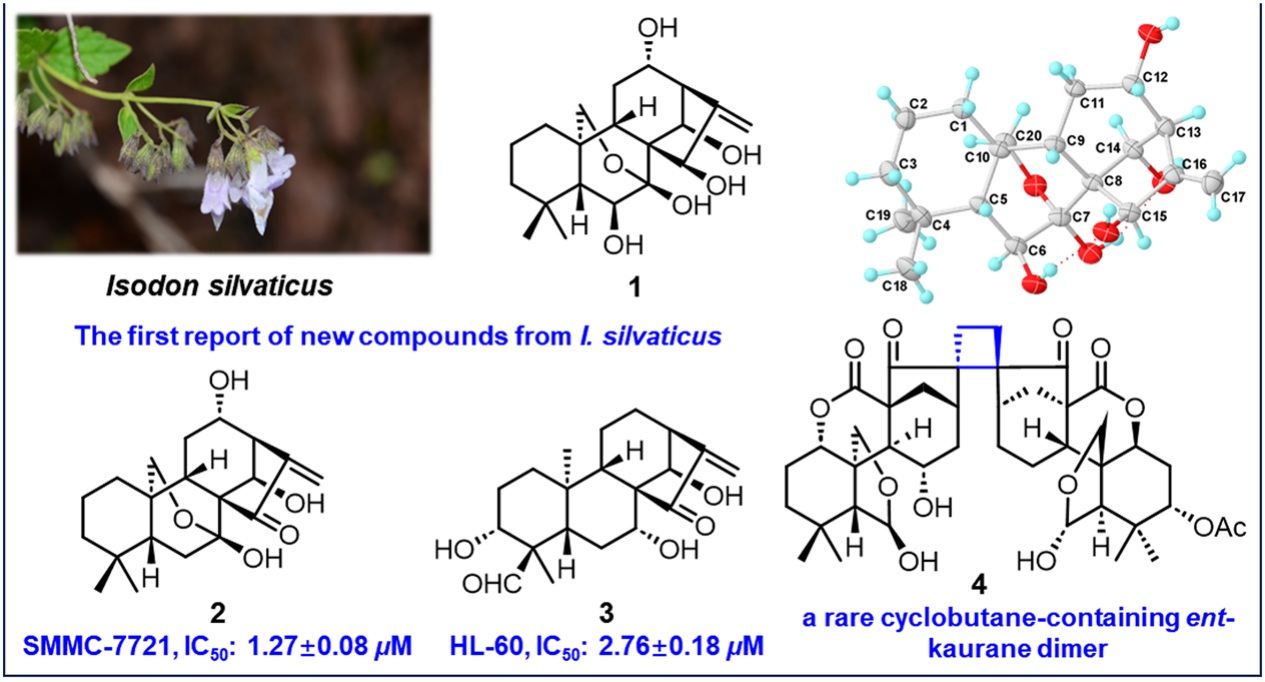

**Supplementary Information:**

The online version contains supplementary material available at 10.1007/s13659-024-00465-9.

## Introduction

*Isodon* species, highly esteemed for their medicinal virtues, harbor a rich array of diterpenoids, with *ent*-kauranoids being the most prominent class [1, 2]. The recent discovery of labdane-based meroditerpenoids with cyclobutane moieties, including isoscopariusins B and C [3], along with clerodane-based dimers scospirosins A and B [4], and scoparicacids A–C [5] from *I. scoparius*, further highlights the chemical versatility of these plants. Furthermore, the bioactivities of these compounds are particularly notable. For example, eriocalyxin B, derived from *I*. *eriocalyx* var. *laxiflora*, has recently been found to demonstrate significant therapeutic potential in oncology by effectively inhibiting cell migration in triple-negative breast cancer [6].

**Fig. 1:**
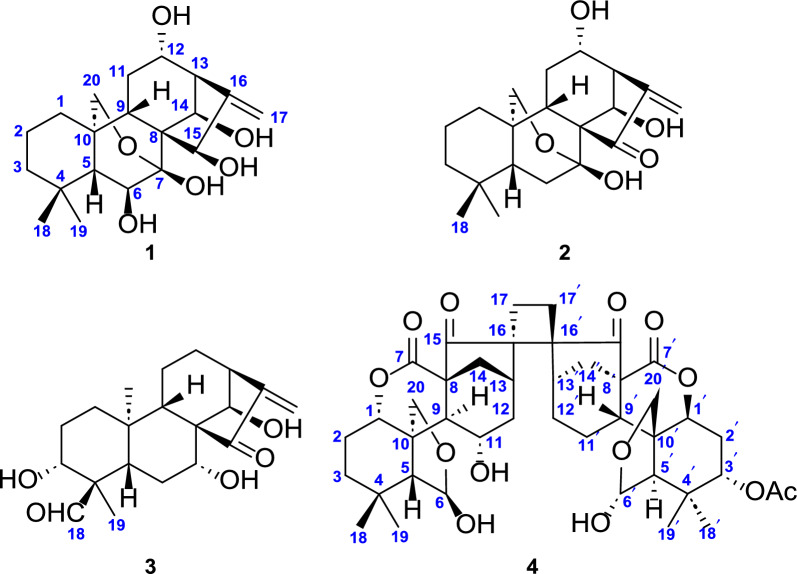
Chemical structures of silvaticusins A–D (**1**–**4**)

In a concerted effort to uncover natural products with distinctive structures and potent bioactivities from the genus *Isodon*, a comprehensive phytochemical study was conducted on *Isodon silvaticus* collected from Changdu in the Tibet Autonomous Region. To date, there has been a scarcity of information regarding the chemical composition of *I. silvaticus*, except that a reported synthetic derivative acetyl-macrocalin B [7] has been isolated from this plant. This compound has undergone an in-depth pharmacological assessment, revealing its remarkable capacity to inhibit the growth of non-small cell lung cancer and esophageal squamous cell carcinoma in both in vitro and in vivo models [8, 9]. The current study resulted in the isolation of silvaticusins A–D (**1**–**4**) (Fig. 1), involving two new C-20-oxygenated-*ent*-kaurane diterpenoids (**1** and** 2**), a new C-20 non-oxygenated-*ent*-kaurane diterpenoid (**3**), and a new dimeric diterpenoid (**4**) characterized by a cyclobutane moiety formed through a [2 + 2] cycloaddition between two 6,7-seco-*ent*-kaurane diterpenoids. Meanwhile, 37 miscellaneous known diterpenoids were characterized from the same plant material (Fig. S44 and Table S13). This study presents the first characterization of new compounds from *I. silvaticus*. Notably, the discovery of silvaticusin D (**4**) enriches the limited array of *ent*-kaurane dimers with a cyclobutane ring. This group now includes maoecrystal M (7,20-epoxy-*ent*-kaurane dimer) [10], bisjaponins A and B (6,7-seco-*ent*-kaurane dimer, 6,7-seco-/7,20-epoxy-*ent*-kaurane dimer, respectively) [11], and bistenuifolin L and M (C-20 non-oxygenated *ent*-kaurane dimers) [12]. The cyclobutane moiety in these compounds, formed through [2 + 2] cycloaddition, is a key structural feature that may confer unique biological properties and synthetic challenges, underscoring their importance in the field of natural product chemistry [13, 14]. The structures of silvaticusins A–D (**1**–**4**) were determined through a combinatorial use of spectroscopic analysis, X-ray diffraction, and quantum chemical calculations. Notably, compounds **2** and **3** were found to exhibit remarkable cytotoxic activities against HL-60, A-549, SMMC-7721, MDA-MB-231, and SW-480 human tumor cell lines with IC_50_ ranging from 1.27 ± 0.08 to 7.52 ± 0.33 μM. Herein, the structure elucidation and bioactivity evaluation of silvaticusins A–D (**1**–**4**) were reported.

## Results and discussion

**Fig. 2:**
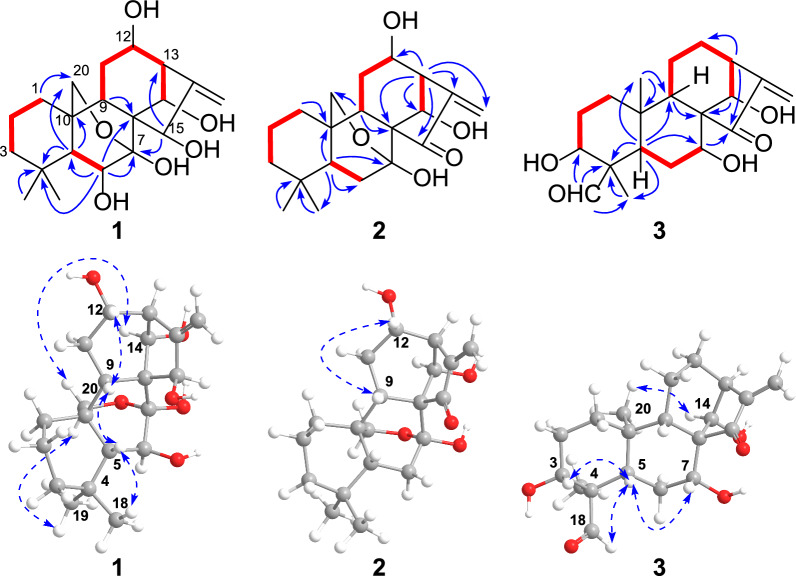
^1^H − ^1^H COSY (bold), selected HMBC (arrow), and key ROESY (dashed arrow) correlations of silvaticusins A–C (**1**–**3**)

Silvaticusin A (**1**), which was obtained as colorless crystals, had the molecular formula C_20_H_30_O_6_ as inferred from the (+)-HRESIMS ion at *m*/*z* 389.1936 [M + Na]^+^ (calcd 389.1535) and supported by ^13^C NMR data. The IR spectrum exhibited characteristic absorption bands indicative of hydroxy (3391 cm^−1^), and olefinic (1633 cm^−1^) functionalities. Its ^1^H NMR spectrum showed resonances of two methyl groups at *δ*_H_ 1.05 (s) and 1.15 (s), four oxygenated methines at *δ*_H_ 4.21 (d, *J* = 5.3 Hz), 4.32 (t, *J* = 8.2 Hz), 5.34 (br s), and 5.72 (br s), and two olefinic protons at *δ*_H_ 5.36 (d, *J* = 1.9 Hz) and 5.74 (d, *J* = 1.9 Hz) (Table 1). Its ^13^C NMR and DEPT spectra featured twenty carbon resonances, including two methyl groups, six methylenes (one olefinic, one oxygenated), seven methines (four oxygenated), five non-protonated carbons (three quaternary) (Table 2). Comprehensive analysis of the 1D and 2D NMR data of **1** manifested that its structure resembled that of the 7,20-epoxy-*ent*-kauranoid, enmenol [15]. The H_2_-1/H_2_-2/H_2_-3, H-5/H-6, and H-9/H_2_-11/H-12/H-13/H-14 correlations in the ^1^H − ^1^H COSY spectrum, in tandem with the H_2_-1/C-20, H-6/C-4, H-6/C-7, H-6/C-8 correlations in the HMBC spectrum (Fig. 2) indicated that silvaticusin A (**1**) possesses a hydroxyl group at the C-12 position, differing from the C-1 hydroxylation in enmenol, while the rest of their structures were consistent.

**Table 1:** ^1^H NMR spectroscopic data of silvaticusins A–C (**1**–**3**) (*δ* in ppm, *J* in Hz)

No	1^a^	2^a^	3^b^
1a	0.89, m	0.82, m	1.93, overlap
1b	1.27, m	1.15 d, (13.5)	1.65, overlap
2a	1.08, m	1.26, m	1.92, overlap
2b	1.31, m	1.26, m	1.92, overlap
3a	1.23, m	1.09, m	4.04, m
3b	1.23, m	1.34, m	–
5	1.56, d, (5.3)	1.41, dd, (11.7, 6.9)	1.59, br d, (12.4)
6a	4.21, d, (5.3)	2.04, m	2.16, q, (12.4)
6b	–	3.57, m	1.74, m
7	–	–	4.80, dt, (12.4, 4.7)
9	2.53, dd, (15.3, 6.3)	1.56, m	1.42, overlap
11a	1.79, m	1.99, m	1.43, overlap
11b	1.95, m	1.99, m	1.52, m
12a	4.32, t, (8.2)	4.26, t, (8.2)	1.66, overlap
12b	–	–	0.83, m
13	3.18, br s	3.52, br s	3.23, br s
14	5.34, br s	5.59, br s	5.09, br s
15	5.72, br s	–	–
17a	5.36, d, (1.9)	5.49, s	6.30, s
17b	5.74, d, (1.9)	6.38, s	5.39, s
18	1.15, s	1.04, s	9.5, s
19	1.05, s	0.77, s	1.39, s
20a	4.07, d, (9.6)	4.09, d, (10.0)	1.07, s
20b	4.12, d, (9.6)	4.31, d, (10.0)	–

^a^Recorded on a 500 MHz NMR spectrometer in pyridine-*d*_5_. ^*b*^Recorded on an 800 MHz NMR spectrometer in pyridine-*d*_5_

**Table 2:** ^13^C NMR spectroscopic data of silvaticusins A–C (**1**–**3**) (*δ* in ppm)

No	1^a^	2^a^	3^b^
1	31.0, CH_2_	30.9, CH_2_	30.3, CH_2_
2	41.5, CH_2_	19.0, CH_2_	26.4, CH_2_
3	19.1, CH_2_	40.9, CH_2_	70.6, CH
4	34.0, C	34.0, C	55.1, C
5	57.8, CH	48.9, CH	43.6, CH
6	73.3, CH	32.3, CH_2_	30.9, CH_2_
7	99.9, C	98.1, C	72.6, CH
8	53.5, C	59.8, C	60.8, C
9	40.0, CH	48.9, CH	53.3, CH
10	36.1, C	36.1, C	37.6, C
11	27.1, CH_2_	28.2, CH_2_	17.0, CH_2_
12	76.5, CH	74.7, CH	36.8, CH_2_
13	57.9, CH	55.3, CH	45.8, CH
14	74.3, CH	71.9, CH	74.5, CH
15	73.3, CH	203.3, C	206.6, C
16	156.3, C	149.7, C	148.5, C
17	111.1, CH_2_	119.1, CH_2_	115.6, CH_2_
18	33.6, CH_3_	20.8, CH_3_	205.8, CH
19	22.5, CH_3_	32.4, CH_3_	8.6, CH_3_
20	65.7, CH_2_	66.4, CH_2_	17.3, CH_3_

^a^Recorded on a 500 MHz NMR spectrometer in pyridine-*d*_5_. ^b^Recorded on a 600 MHz NMR spectrometer in pyridine-*d*_5_

**Fig. 3:**
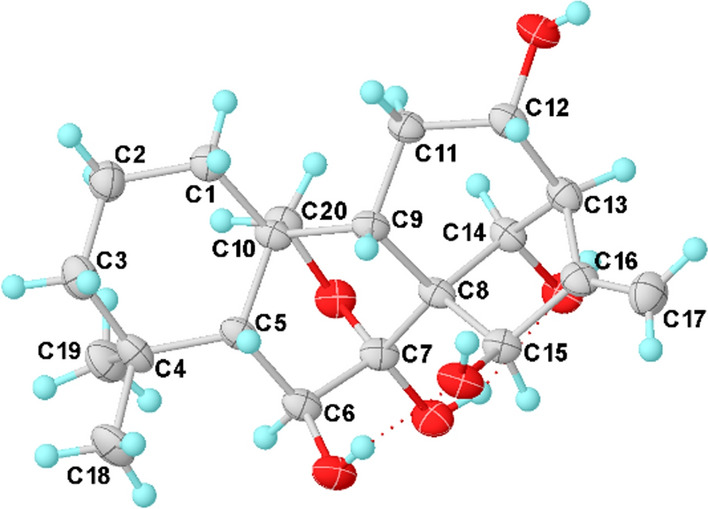
Crystallographic structure of silvaticusin A (**1**)

As for the configurational determination of **1**, assigning H-5 as *β*-oriented, the ^3^*J*_H-5/H-6_ (5.3 Hz) indicated that H-6 was *α*-oriented. The H-9/H-5*β*, H-12/H-9, H_3_-18/H-5*β*, H-20b/H_3_-19, and H-14/H-20a correlations in the ROESY spectrum indicated that H-9, H-12, HO-14 adopted *β* orientation, while C-20 adopted *α* orientation. But the orientation of HO-15 could not be conclusively assigned. Fortunately, well-formed crystals of compound **1** were successfully procured using methanol and subsequently underwent X-ray crystallographic analysis. The refined Cu K*α* data yielded a Flack parameter of –0.13(15), which enabled the determination of its absolute configuration as 5*R*, 6*S*, 7*R*, 9*R*, 10*R*, 12*S*, 13*R*, 14*R*, and 15*R* (Fig. 3). Thus, the structure of silvaticusin A (**1**) was defined as 6*β*,7*β*,12*α*,14*β*,15*β*-pentahydroxy-7,20-epoxy-*ent*-kaur-16-ene.

Silvaticusin B (**2**), which was obtained as a white amorphous powder, had the molecular formula C_20_H_28_O_5_ as established by the ( +)-HRESIMS ion at *m*/*z* 349.2010 [M + H]^+^ (calcd 349.2010). The ^1^H and ^13^C NMR data of compound **2** (Tables 1 and 2) were similar to those of **1**. The ^1^H − ^1^H COSY spectrum exhibited correlations of H_2_-1/H_2_-2/H_2_-3, H-5/H_2_-6, H-9/H_2_-11/H-12, and H-13/H-14 (Fig. 2). And the HMBC spectrum displayed correlations from H-5 to C-6, C-7, C-10, and C-19, from H-13 to C-8, C-12, C-14, C-15, C-16 and C-17. These findings demonstrated that compound **2** features a carbonyl at C-15 instead of a hydroxy group as in **1**, and a methylene at C-6 instead of the oxygenated methine in **1**. Analysis of the ROESY spectrum of **2** uncovered the *β* orientations of H-12, HO-14. Thus, the structure of silvaticusin B (**2**) was assigned as 7*β*,12*α*,14*β*-trihydroxy-7,20-epoxy-*ent*-kaur-16-en-15-one.

Silvaticusin C (**3**) was isolated as a white amorphous powder. The molecular formula C_20_H_28_O_5_ was deduced from HREIMS and ^13^C NMR data. A thorough examination of the ^1^H and ^13^C NMR data for compound **3** revealed that its structure bore a close resemblance to that of the C-20 non-oxygenated-*ent*-kaurane diterpenoid macroclyxin C [16], sharing an identical molecular weight of 348 Daltons. The ^1^H − ^1^H COSY spectrum, with key correlations of H_2_-1/H_2_-2/H-3, H-5/H_2_-6/H-7, and H-9/H_2_-11/H_2_-12/H-13/H-14, and the HMBC spectrum (Fig. 2), showing correlations from CHO-18 to C-3, C-4, and C-19, and from H-13 to C-12 and C-15, have pinpointed a hydroxyl group at C-12 in silvaticusin C (**3**). This differs from macroclyxin C's hydroxyl group at C-3, with the rest of their structures remains congruent. As for the stereochemistry of **3**, appointing H-5 as *β*-oriented, the H-3/H-5*β*, H-7/H-5*β*, H-9/H-5*β*, CHO-18/H-5*β*, H_3_-20/H_3_-19, H-14/H_3_-20 correlations in the ROESY spectrum indicated the *β* orientation of H-3, H-7, CHO-18, and HO-14. Therefore, the structure of silvaticusin C (**3**) was assigned as 3*α*,7*α*,14*β*-trihydroxy-*ent*-kaur-16-en-18-al-15-one.

**Fig. 4:**
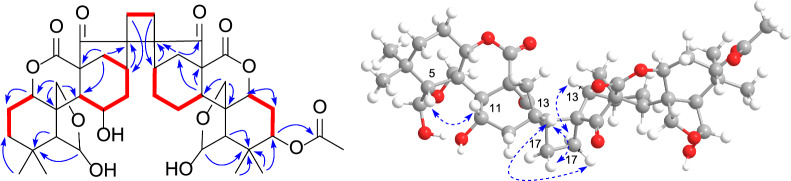
^1^H − ^1^H COSY (bold), selected HMBC (arrow), and key ROESY (dashed arrow) correlations of silvaticusin D (**4**)

Silvaticusin D (**4**) was isolated as a white amorphous powder. The molecular formula of C_42_H_55_O_14_ was inferred from the (+)-HRESIMS ion at *m*/*z* 789.3458 [M + Na]^+^ (calcd 789.3457) and ^13^C NMR data. Detailed analysis of the ^1^H and ^13^C NMR data of **4** suggested that it is a diterpene dimer composed of two 6,7-seco-6,20-epoxy-*ent*-kauranoids analogous to the structures of epinodosin [17, 18] and enmein-3-acetate [19], respectively. This deduction was further supported by H-1/H_2_-2/H_2_-3, and H-9/H-11/H_2_-12/H-13/H-14, H-1′/H_2_-2′/H-3′, and H-9′/H_2_-11′/H_2_-12′/H-13′/H-14′ correlations in the ^1^H − ^1^H COSY spectrum, as well as HMBC correlations from H-6 to C-4, C-5, C-10, C-20, from H-11 to C-8, C-10, C-13, from H-6′ to C-4′, C-10′, C-20′, from H-3′ to C-1′, C-2′, C-4′, C-5′, -OAc. The lack of NMR signals for the typical terminal double bonds at C-16/C-17, a feature usually presents in *ent*-kauranoids, in both monomers of compound **4**, implies that these double bonds are involved in the dimerization. This, combined with the one remaining degree of unsaturation and key ^1^H − ^1^H COSY correlation between H-17 and H-17′, along with HMBC correlations of H-13/C-16, H_2_-17/C-13, H-13′/C-16′, and H_2_-17′/C-13′, supported a head-to-head [2 + 2] cycloaddition mechanism for dimerization. Accordingly, the planar structure of compound **4** is established as illustrated in Fig. 4.

**Fig. 5:**
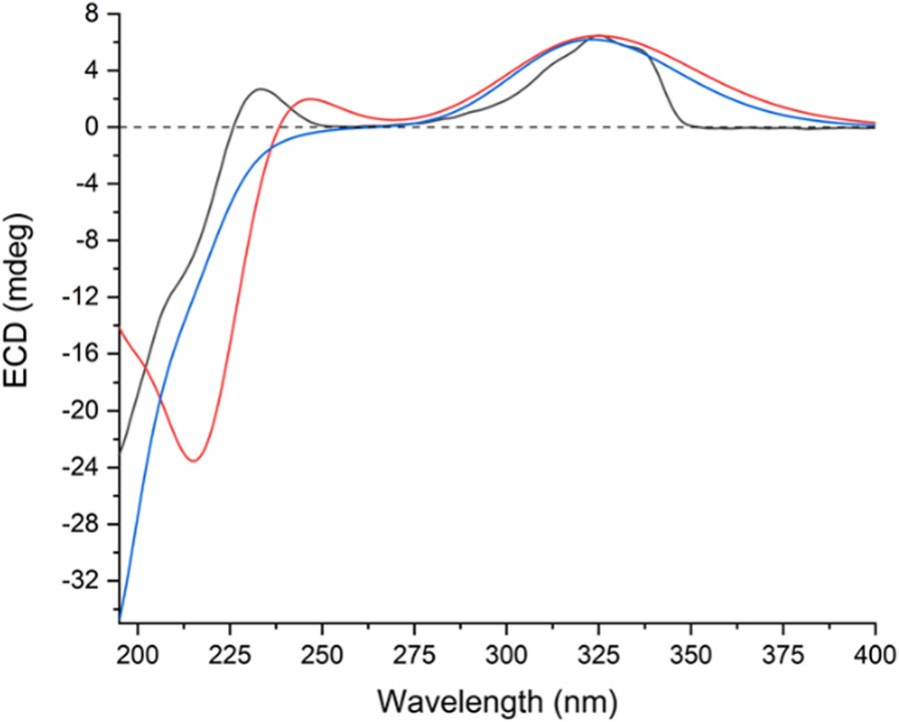
Experimental ECD spectrum of silvaticusin D (**4**) (black). Calculated ECD spectra of (1*S*, 5*R*, 6*R*, 8*S*, 9*S*, 10*S*, 11*S*, 13*S*, 16*R*, 1′*S*, 3′*S*, 5′*R*, 6′*R*, 8′*S*, 9′*S*, 10′*S*, 13′*R*, 16′*R*)-**4** (**4A**) at PBE0-SCRF/6–311 + G(2d,p) (red) and CAM-B3LYP-SCRF/6–311 + G(2d,p) (blue, shift =  + 15 nm) level of theory

The stereochemistry of most chiral centers was determined using ROESY correlations and coupling constants, except for C-3′, where overlapping NMR signals obscured its configuration determination. As for the two new chiral centers (C-16 and C-16′) formed during dimerization, correlations of H-13/H-17a, H-13/H-17′a, and H-13′/H-17′b in the ROESY spectrum suggested the *α* orientation of C-17 methylene, and the *β* orientation of C-17′. Then, the two possible epimers, (1S*, 5R*, 6R*, 8S*, 9S*, 10S*, 11S*, 13S*, 16R*, 1′S*, 3′S*, 5′R*, 6′R*, 8′S*, 9′S*, 10′S*, 13′R*, 16′R*)-**4** (**4a**) and (1S*, 5R*, 6R*, 8S*, 9S*, 10S*, 11S*, 13S*, 16R*, 1′S*, 3′R*, 5′R*, 6′R*, 8′S*, 9'S*, 10′S*, 13′R*, 16′R*)-**4** (**4b**) were subjected to NMR computation at the mPW1PW91-SCRF/6–31 + G(d,p)//B3LYP-D3BJ/6-31G(d) level of theory. In the subsequent DP4 + analysis [20] comparing isomers **4a** and **4b**, **4a** exhibited a conclusive probability of 100% (Figure S46), thereby substantiating its designation as the correct structure. Then, TDDFT ECD calculation of (1*S*, 5*R*, 6*R*, 8*S*, 9*S*, 10*S*, 11*S*, 13*S*, 16*R*, 1′*S*, 3′*S*, 5′*R*, 6′*R*, 8′*S*, 9′*S*, 10′*S*, 13′*R*, 16′*R*)-**4** (**4A**) were carried out at the PBE0-SCRF/6–311 + G(2d,p) and CAM-B3LYP-SCRF/6–311 + G(2d,p) level of theory, respectively, and the calculated curves matched the experimental curve (Fig. 5), thereby determining the absolute configuration of compound **4** as 1*S*, 5*R*, 6*R*, 8*S*, 9*S*, 10*S*, 11*S*, 13*S*, 16*R*, 1′*S*, 3′*S*, 5′*R*, 6′*R*, 8′*S*, 9′*S*, 10′*S*, 13′*R*, and 16′R (Table 3).

**Table 3:** ^1^H and ^13^C NMR spectroscopic data of silvaticusin D (**4**) (*δ* in ppm)

No	*δ*_H_ (mult., *J* in Hz)	*δ*_C_, type	No	*δ*_H_ (mult., *J* in Hz)	*δ*_C_, type
1	5.65, m	78.5, CH	2′a	2.13, m	27.2, CH_2_
2a	1.83, m	23.7, CH_2_	2′b	2.27, (12.9)	–
2b	2.04, s	–	3′	5.01, overlap	77.3, CH
3a	1.30, m	37.1, CH_2_	4′	–	34.5, C
3b	1.46, m	–	5′	2.42, d, (3.3)	51.2, CH
4	–	31.3, C	6′	5.78, d, (3.3)	101.8, CH
5	2.71, d, (3.1)	55.6, CH	7′	–	172.0, C
6	5.77, d, (3.1)	101.8, CH	8′	–	58.0, C
7	–	172.4, C	9′	2.84, m	47.6, CH
8	–	57.8, C	10′	–	49.7, C
9	2.80, d, 3.7	49.7, CH	11′a	2.11, m	19.1, CH_2_
10	–	49.6, C	11′b	1.56, m	
11	5.00, m	65.1, CH_2_	12′a	1.36, m	21.6, CH_2_
12a	1.75, m	33.3, CH_2_	12′b	1.37, m	
12b	2.09, m	–	13′	3.12, d, (11.1)	33.9, CH
13	3.32, m	34.2, CH	14′a	2.47, d, (13.3)	32.2, CH_2_
14a	2.55, m	33.4, C	14′b	2.50, d, (13.3)	
14b	3.80, m	–	15′	–	216.5, C
15	–	216.8, C	16′	–	63.2, C
16	–	63.3, C	17′a	1.91, m	23.9, CH_2_
17a	2.20, m	24.3, CH_2_	17′b	2.21, s	
17b	1.89, m	–	18′	1.02, s	27.3, CH_3_
18	0.98, s	23.1, CH_3_	19′	0.99, s	22.5, CH_3_
19	0.96, s	32.9, CH_3_	20′a	4.46, d, (9.0)	74.3, CH_2_
20a	4.27, d, (9.0)	73.9, CH_2_	20′b	4.25, d, (9.0)	
20b	4.52, d, (9.0)	–	3′-OAc	–	169.8, C
1′	4.88, m	72.9, CH		2.23, s	20.6, CH_3_

Recorded on an 800 MHz NMR spectrometer in pyridine-*d*_5_

Previous research on diterpenoids from the *Isodon* genus has shown that the *exo*-methylene cyclopentanone D-ring system is crucial for their antitumor and anti-inflammatory effects, with C-14 hydroxylation usually enhancing these effects [21]. In this research, compounds **2** and **3**, which possess this D-ring system, were evaluated for cytotoxicity against HL-60, A-549, SMMC-7721, MDA-MB-231, and SW-480 cell lines using the MTS assay [22], with cisplatin and paclitaxel as positive controls. Compounds **2** and **3** exhibited significant cytotoxic activity, with IC_50_ values ranging from 1.27 ± 0.08 to 7.52 ± 0.33 μM for all five cell lines (Table 4).

**Table 4:** Cytotoxic activities of compounds **2** and **3** against five human tumor cell lines

Compound	HL-60	A-549	SMMC-7721	MDA-MB-231	SW-480
**2**	1.65 ± 0.02	3.23 ± 0.14	1.27 ± 0.08	1.99 ± 0.05	2.51 ± 0.14
**3**	2.76 ± 0.18	7.52 ± 0.33	4.13 ± 0.09	5.43 ± 0.11	4.48 ± 0.11
Paclitaxel	< 0.008	< 0.008	0.095 ± 0.007	< 0.008	< 0.008
Cisplatin	2.92 ± 0.15	11.95 ± 0.25	20.52 ± 0.63	19.28 ± 0.30	21.32 ± 1.05

Results are expressed as IC_50_ values in *μ*M. Cisplatin and paclitaxel were used as positive controls

## Experimental

### General experimental procedures

General experimental procedures can be found in the Supplementary Material (Part 1).

### Plant material

The source of the plant material can be found in the Supplementary Material (Part 2).

### Extraction and isolation

The detailed procedures for extraction and isolation can be found in in the Supplementary Material (Part 3).

### Characteristic data of compounds 1–4

Silvaticusin A (**1**): colorless needles; mp 195 ~ 197 °C; $[\alpha]_{\text{D}}^{20}$ − 16.04 (*c* 0.106, MeOH); IR (KBr) *ν*_max_ 3391, 2948, 2929 cm^−1^; ^1^H and ^13^C NMR data, see Tables 1 and 2; positive-ion HREIMS [M + Na]^+^ *m*/*z* 389.1936 (calcd for C_20_H_30_O_6_Na, 389.1935).

Crystallographic data for silvaticusin A (**1**): C_20_H_30_O_6_•2(H_2_O), *M* = 402.47, *a* = 6.4305(2) Å, *b* = 16.4111(5) Å, *c* = 18.9447(5) Å, *α* = 90°, *β* = 90°, *γ* = 90°, *V* = 1999.26(10) Å^3^, *T* = 150.(2) K, space group *P*212121, *Z* = 4, *μ*(Cu Kα) = 0.851 mm^−1^, 14,239 reflections measured, 3632 independent reflections (*R*_*int*_ = 0.1309). The final *R*_*1*_ values were 0.0415 (*I* > 2*σ*(*I*)). The final *wR*(*F*^2^) values were 0.1046 (*I* > 2*σ*(*I*)). The final *R*_*1*_ values were 0.0639 (all data). The final *wR*(*F*^2^) values were 0.1146 (all data). The goodness of fit on *F*^2^ was 1.042. Flack parameter = − 0.13(15).

Silvaticusin B (**2**): white, amorphous powder; $[\alpha]_{\text{D}}^{20}$ − 48.66 (*c* 0.06, MeOH); IR (KBr) *ν*_max_ 3427, 2948, 2928, 1724 cm^−1^; ^1^H and ^13^C NMR data, see Tables 1 and 2; positive-ion HREIMS [M + H]^+^ *m*/z 349.2010 (calcd for C_20_H_29_O_5_, 349.2010).

Silvaticusin C (**3**): white, amorphous powder; $[\alpha]_{\text{D}}^{25}$ − 73.33 (*c* 0.09, MeOH); ^1^H and ^13^C NMR data, see Tables 1 and 2; positive-ion HREIMS [M + H]^+^ *m*/*z* 349.2008 (calcd for C_20_H_29_O_5_, 349.2010).

Silvaticusin D (**4**): white, amorphous powder; $[\alpha]_{\text{D}}^{25}$ − 22.61 (*c* 0.092, MeOH); ^1^H and ^13^C NMR data, see Table 3; positive-ion HREIMS [M + Na]^+^ *m*/*z* 789.3458 (calcd for C_42_H_55_O_14_Na, 789.3457).

### X-ray crystal structure analysis

The X-ray crystal structure analysis of silvaticusin A (**1**) can be found in in the Supplementary Material (Part 4).

### The cytotoxicity assay

The cytotoxicity assay has been described previously [23].

### Supplementary Information

Supplementary Material 1. Supplementary data associated with this article (^1^H, ^13^C NMR, DEPT, HSQC, HMBC, ^1^H − ^1^H COSY, NOESY, HREIMS, UV, ECD and IR spectra of silvaticusins A–D (**1**–**4**); Computational data of silvaticusin D (**4**)).

## Data Availability

The data that support the findings of this study are openly available in the Science Data Bank at https://doi.org/10.57760/sciencedb.09413.
