# Supplementary material for: Silvaticusins A–D: ent-kaurane diterpenoids and a cyclobutane-containing ent-kaurane dimer from Isodon silvaticus
Source: Nat Prod Bioprospect. 2024 Aug 15;14(1):45. doi: 10.1007/s13659-024-00465-9 (PMC11324621; doi:10.1007/s13659-024-00465-9)
Supplement: Supplementary file 1 — Supplementary Material 1. Supplementary data associated with this article (1H, 13C NMR, DEPT, HSQC, HMBC, 1H − 1H COSY, NOESY, HREIMS, UV, ECD and IR spectra of silvaticusins A–D (1–4); Computational data of silvaticusin D (4)). [file 13659_2024_465_MOESM1_ESM.docx]

**Supplementary Material**

**Silvaticusins A–D: *ent*-kaurane diterpenoids and a** **cyclobutane-containing *ent*-kaurane dimer from *Isodon silvaticus***

Qi-Xiu Hai^1,2^, Kun Hu^2^, Su-Ping Chen^2^, Yang-Yang Fu^2^, Xiao-Nian Li^2^, Han-Dong Sun^2^, Hong-Ping He^1,*^, Pema-Tenzin Puno^2,*^

^1^College of Chinese Materia Medica and Yunnan Key Laboratory of Southern Medicinal Utilization, Yunnan University of Chinese Medicine, Kunming 650500, People’s Republic of China

^2^Key Laboratory of Phytochemistry and Natural Medicines, Kunming Institute of Botany, Chinese Academy of Sciences, Kunming 650201, People’s Republic of China

*Correspondence:

Hong-Ping He

[95431111@qq.com](mailto:95431111@qq.com)

Pema-Tenzin Puno

[punopematenzin@mail.kib.ac.cn](mailto:punopematenzin@mail.kib.ac.cn)

**Contents of Supplementary Material**

[**1.** **General experimental procedures** 1](#_Toc170918288)

[**2.** **Plant material** 1](#_Toc170918289)

[**3.** **Extraction and isolation** 1](#_Toc170918290)

[**4.** **X-ray crystal structure analysis** 2](#_Toc170918291)

[**5.** **NMR, MS, UV, ECD, IR spectra, and OR of silvaticusin A (1)** 3](#_Toc170918292)

[**6.** **NMR, MS, UV, ECD, IR spectra, and OR of silvaticusin B (2)** 9](#_Toc170918293)

[**7.** **NMR, MS, UV, ECD spectra, and OR of silvaticusin C (3)** 15](#_Toc170918294)

[**8.** **NMR, MS, UV, ECD spectra, and OR of silvaticusin D (4)** 21](#_Toc170918295)

[**9.** **Computational data of silvaticusin D (4)** 27](#_Toc170918296)

[**10.** **Information of known compounds** 54](#_Toc170918297)

1. **General experimental procedures**

Melting points were obtained on an XRC-1 apparatus and are uncorrected; Optical rotations were measured in MeOH with Horiba SEPA-300 and JASCO P-1020 polarimeters. UV spectra were obtained on a Shimadzu UV-2401A UV spectrometer; IR spectra were obtained on a Tenor27 infrared spectrometer. ESIMS and HRESIM were measured using an API-QSTAR-TOF mass spectrometer; NMR spectra were recorded on Bruker DRX-500, DRX-600, and DRX-800 spectrometers using TMS as the internal standard. All chemical shifts (*δ*) are expressed in ppm relative to the solvent signals; X-ray diffraction data were collected utilizing a Bruker APEX DUO instrument; The analytical and semi preparative HPLC were Agilent 1100, 1200 and 1260 HPLC, with a Zorbax SB-C18 column (Agilent, 4.6 mm x 250 mm, 1 mL/min; 9.4 mm x 250 mm, 3 mL/min) and a diode array detector (DAD) as the detector; The preparative HPLC is Agilent 1260 HPLC, with a Zorbax SB-C18 (21.2 mm x 250 mm) column. The normal phase silica gel plate for thin-layer chromatography, silica gel for sample mixing, and silica gel for column chromatography are all produced by Qingdao Marine Chemical, Inc.; The solvents used include petroleum ether, chloroform, acetone, ethyl acetate, and methanol, all of which are industrial grade solvents and are used after redistillation; Lichoprep RP-18 (40-63 um) is produced by Merk Company; MCI (75-150 um) is produced by Mitsubishi Chemical Company in Japan; Thin-layer chromatography was performed on precoated TLC plates (200−250 μm thickness, silica gel 60 F254, Qingdao Marine Chemical, Inc.), and spots were visualized by UV light (254 nm) or by spraying heated silica gel plates with 10% H_2_SO_4_ in ethanol.

1. **Plant material**

The aerial parts of *Isodon silvaticus* were collected in the Changdu of the Tibetan Autonomous Region, People’s Republic of China, in August 2020 and identified by Prof. Chun-Lei Xiang at the Kunming Institute of Botany. A specimen (KIB 2020081713) has been deposited in in our laboratory.

1. **Extraction and isolation**

The aerial parts of *I. silvaticus* (13.5 kg) were extracted with 70% aqueous acetone three times (for three days each time) at room temperature and filtered. The filtrate was concentrated under reduced pressure to a volume of 5 L and then partitioned between ethyl acetate and *n*-butanol. The ethyl acetate-soluble portion (750 g) was subjected to silica gel column chromatography (CC) (6.75 kg, 80~100 mesh), eluting with dichloromethane/acetone (1:0~0:1 gradient system). Seven fractions (A–E) were obtained from the silica gel column. Fraction B (chloroform/acetone, 9:1; 242 g) which was a brown gum, individually decolorized on MCI gel, after which they were eluted with methanol/water (30%, 60%, 90%, 100% and 100% acetone) to yield fractions B1–B5. Fraction B2 (161 g) was separated into six subfractions (B2-1–B2-6) using silica gel CC (petroleum ether/acetone, 10:1 to 0:1 gradient). B2-2 (2.04 g) was subjected to semipreparative HPLC (acetonitrile/water) to obtain **3** (1.6 mg). Fraction C (chloroform/acetone, 8:2; 142 g) which was a brown gum, individually decolorized on MCI gel, after which they were eluted with methanol/water (20%, 40%, 60%, 100%) to yield fractions C1–C4. Fraction C2 (48 g) was subjected to RP-18 CC (methanol/water, 25:75 to 90:10 gradient) and then semipreparative HPLC (acetonitrile/water) to yield **2** (4.2 mg), and C3 (36 g) was submitted to semipreparative HPLC (methanol/water) to obtain **1** (801.2 mg) and **4** (1.1 mg).

1. **X-ray crystal structure analysis**

The intensity data for silvaticusin A (**1**) was collected at 100 K on a Bruker APEX DUO diffractometer equipped with an APEX II CCD using Cu K*α* radiation. Cell refinement and data reduction were performed with Bruker SAINT. The structures were solved by direct methods using SHELXS-97, expanded using difference Fourier techniques, and refined by the program and full-matrix least-squares calculations. The non-hydrogen atoms were refined anisotropically, and hydrogen atoms were fixed at calculated positions.

1. **NMR, MS, UV, ECD, IR spectra, and OR of** **silvaticusin A (1)**

**
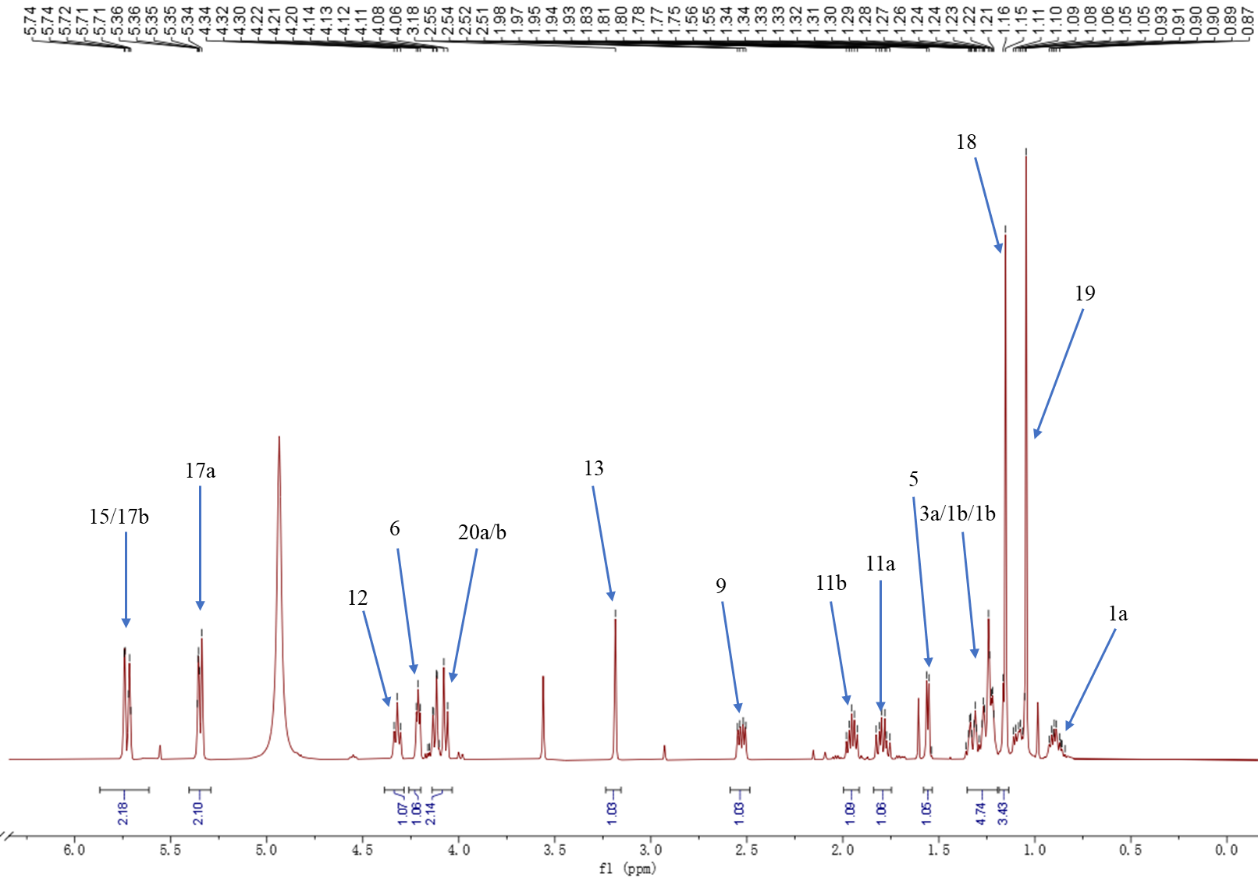
**

**Fig. S1** ^1^H NMR spectrum of silvaticusin A (**1**) (pyridine-*d*_5_, 500 MHz).

**
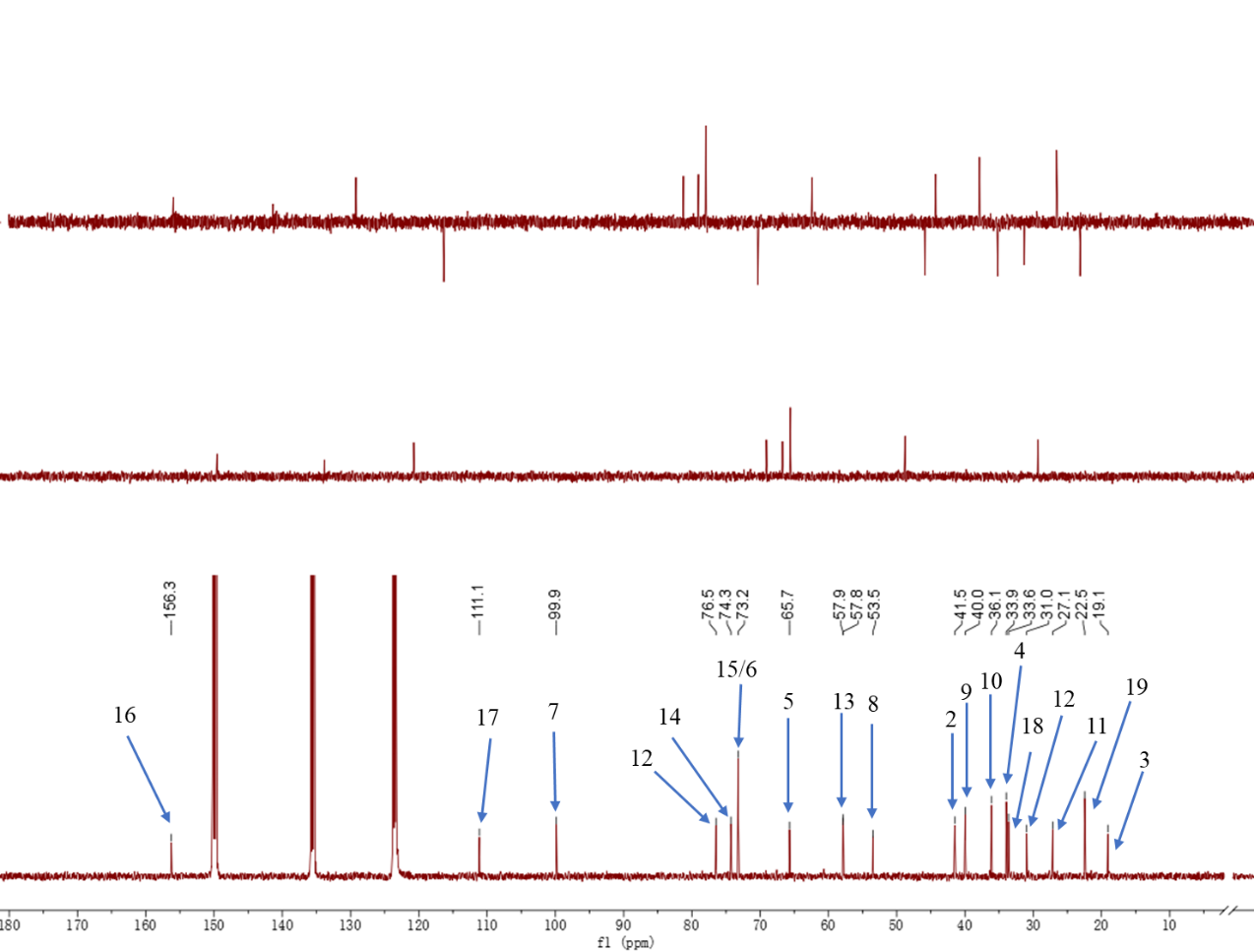
**

**Fig. S2** ^13^C NMR spectrum of silvaticusin A (**1**) (pyridine-*d*_5_, 125 MHz).

**
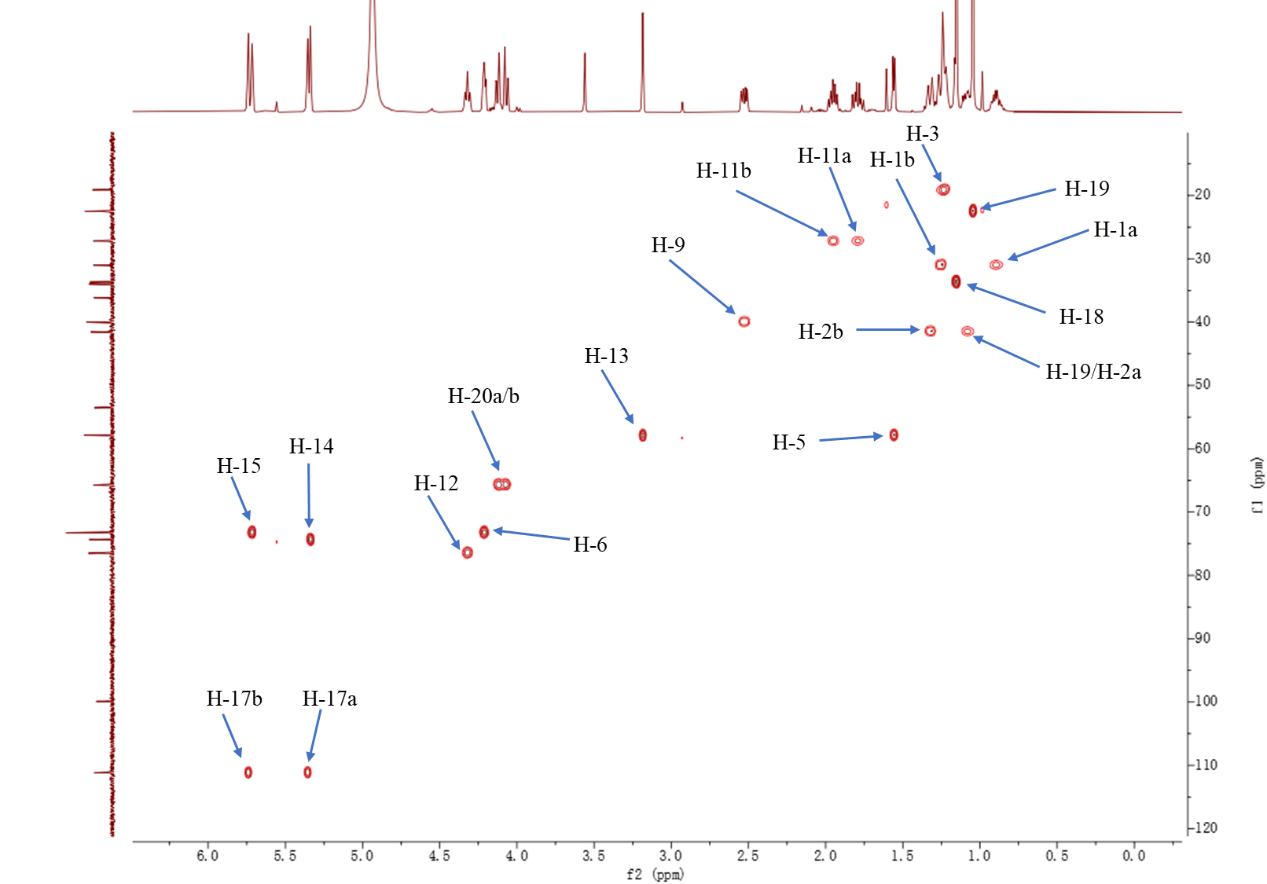
**

**Fig. S3** HSQC spectrum of silvaticusin A (**1**) (pyridine-*d*_5_, 500 MHz).

**
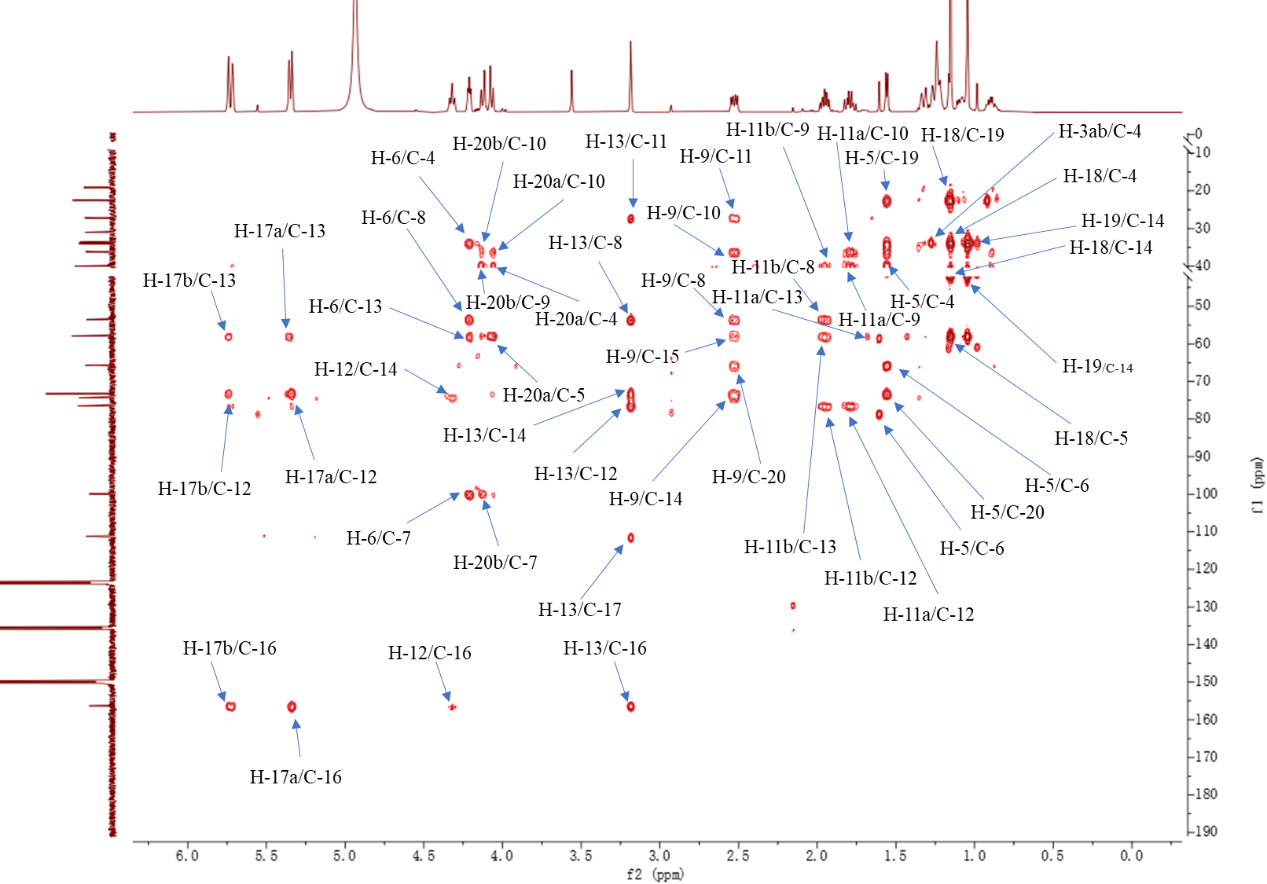
**

**Fig. S4** HMBC spectrum of silvaticusin A (**1**) (pyridine-*d*_5_, 500 MHz).

**
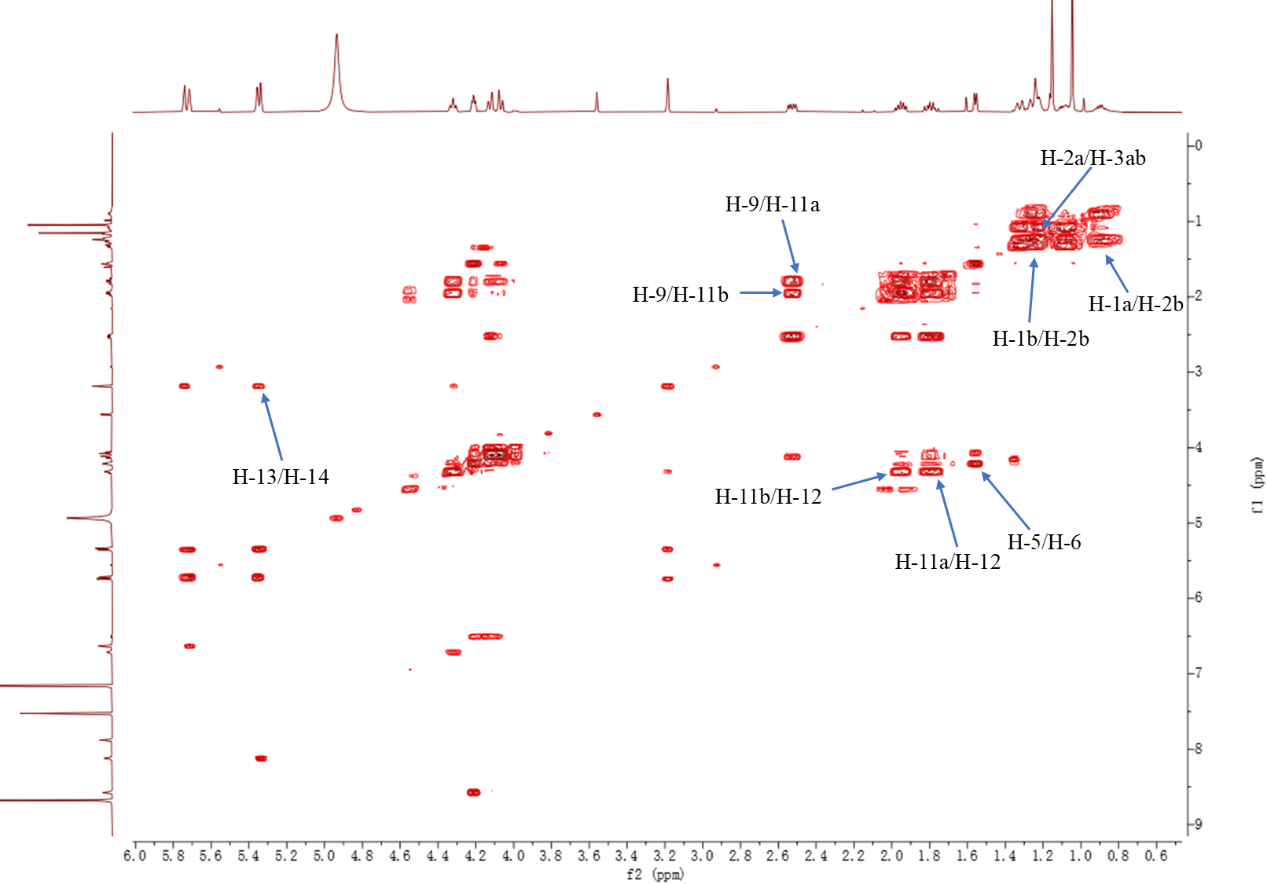
**

**Fig. S5** ^1^H–^1^H COSY spectrum of silvaticusin A (**1**) (pyridine-*d*_5_, 500 MHz).

**
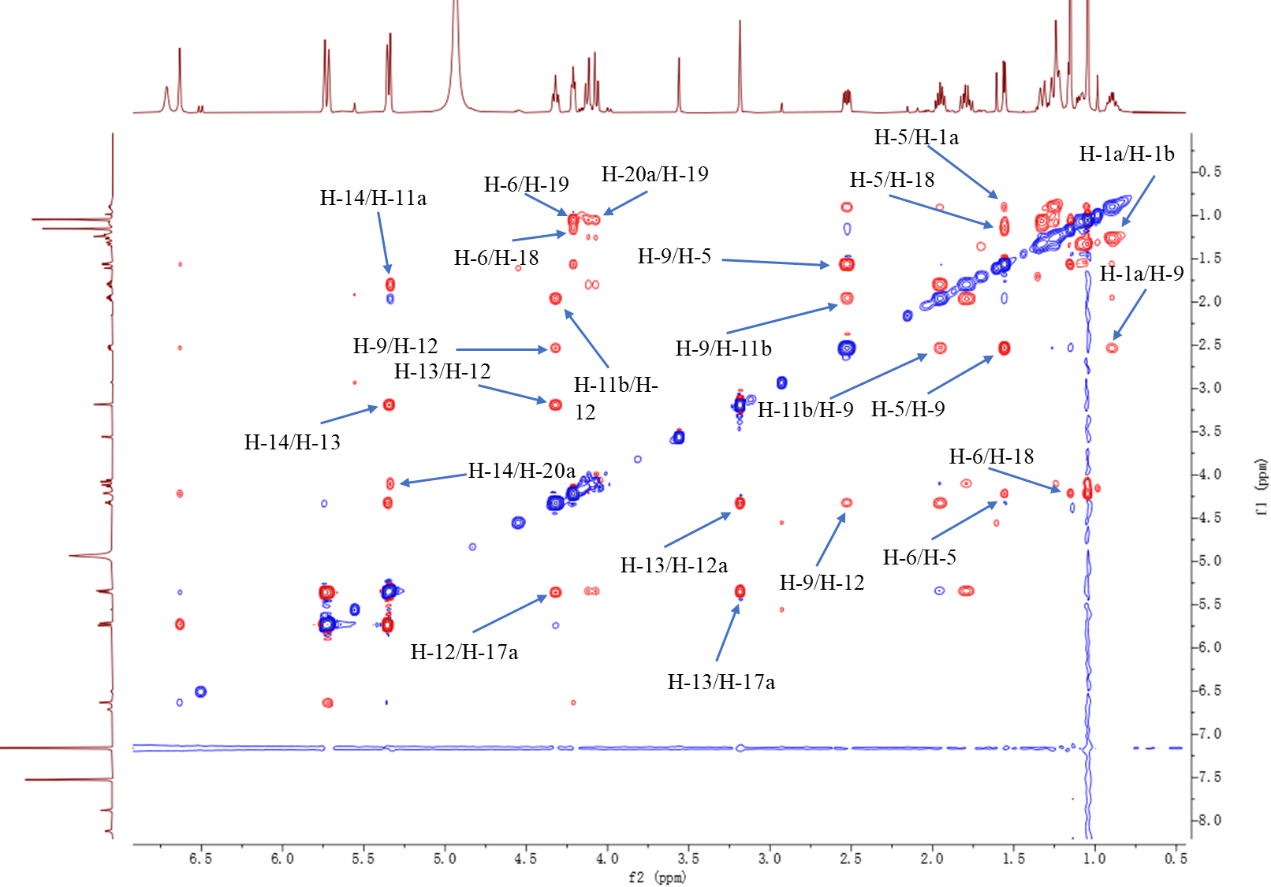
**

**Fig. S6** ROESY spectrum of silvaticusin A (**1**) (pyridine-*d*_5_, 500 MHz).


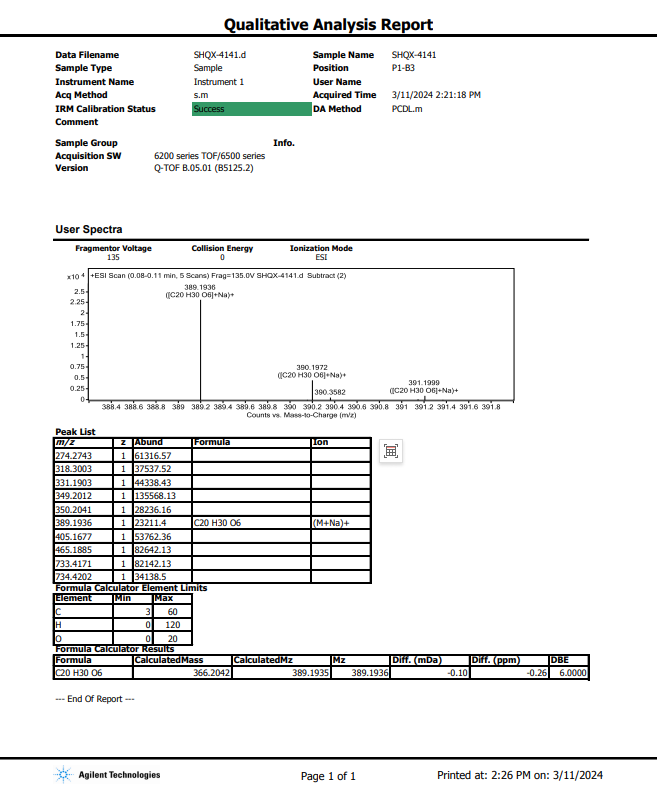


**Fig. S7** HRESIMS spectrum of silvaticusin A (**1**).


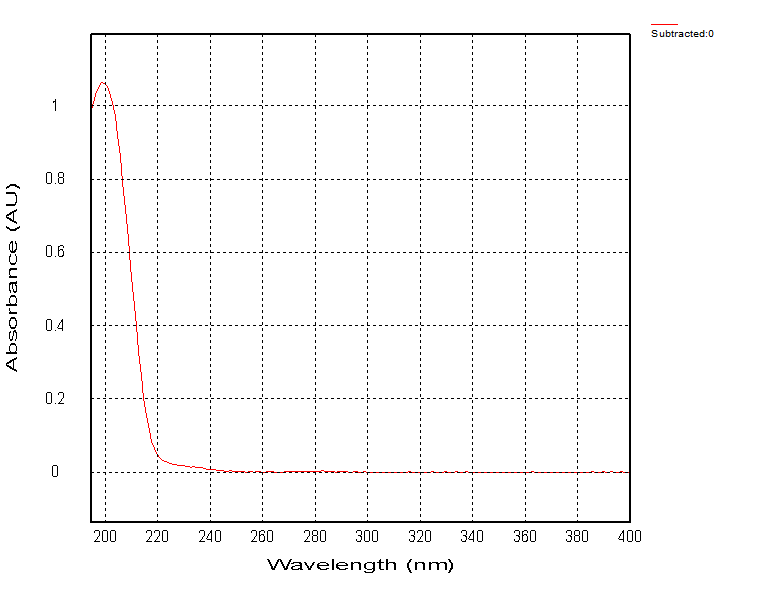


**Fig. S8** UV spectrum of silvaticusin A (**1**).


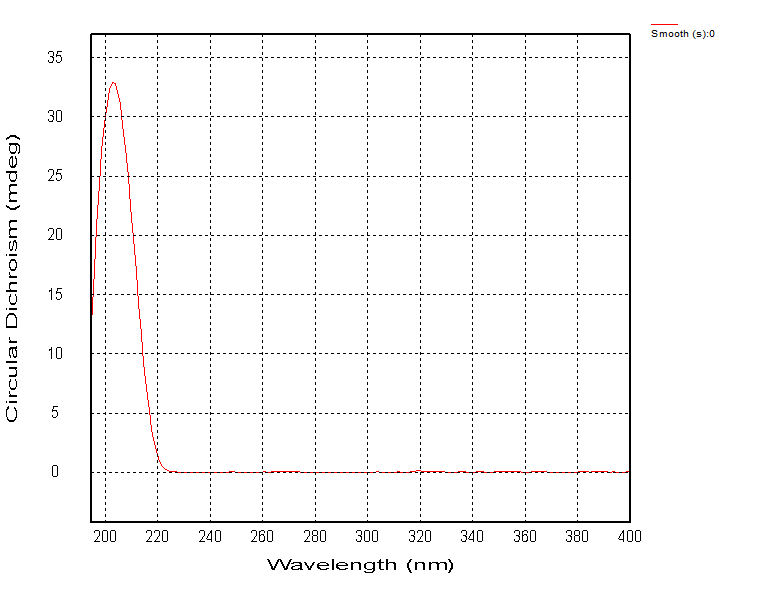


**Fig. S9** ECD spectrum of silvaticusin A (**1**).

**Fig. S10** IR spectrum of silvaticusin A (**1**).


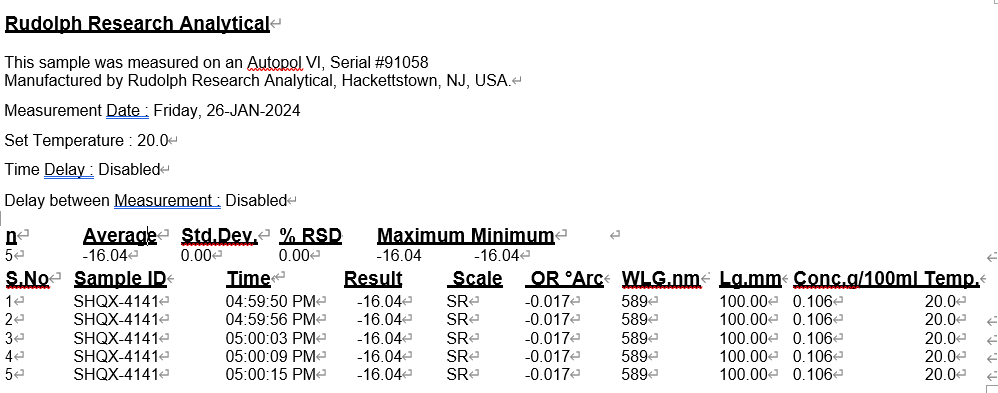


**Fig. S11** OR of silvaticusin A (**1**).

1. **NMR, MS, UV, ECD, IR spectra, and OR of silvaticusin B (2)**

**
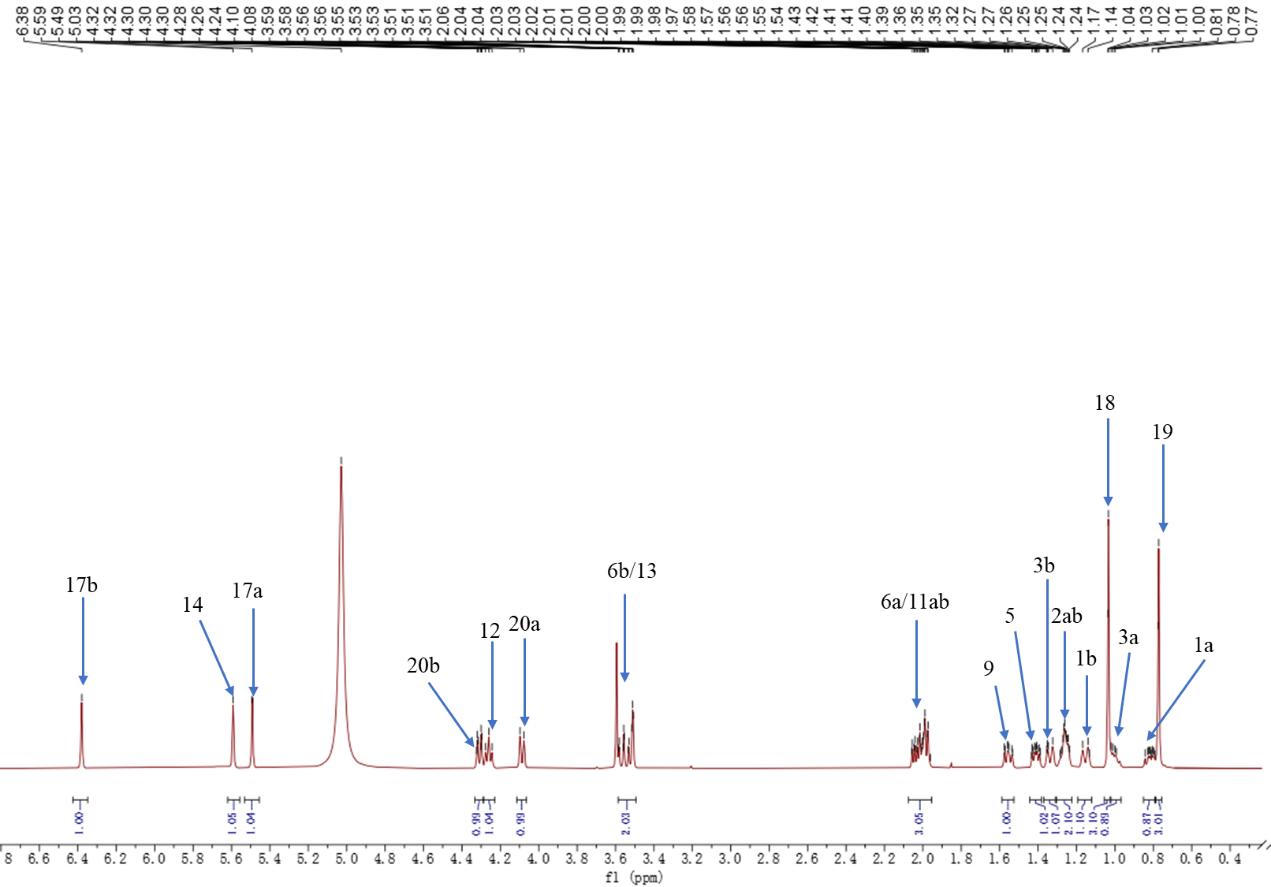
**

**Fig. S12** ^1^H NMR spectrum of silvaticusin B (**2**) (pyridine-*d*_5_, 500 MHz).

**
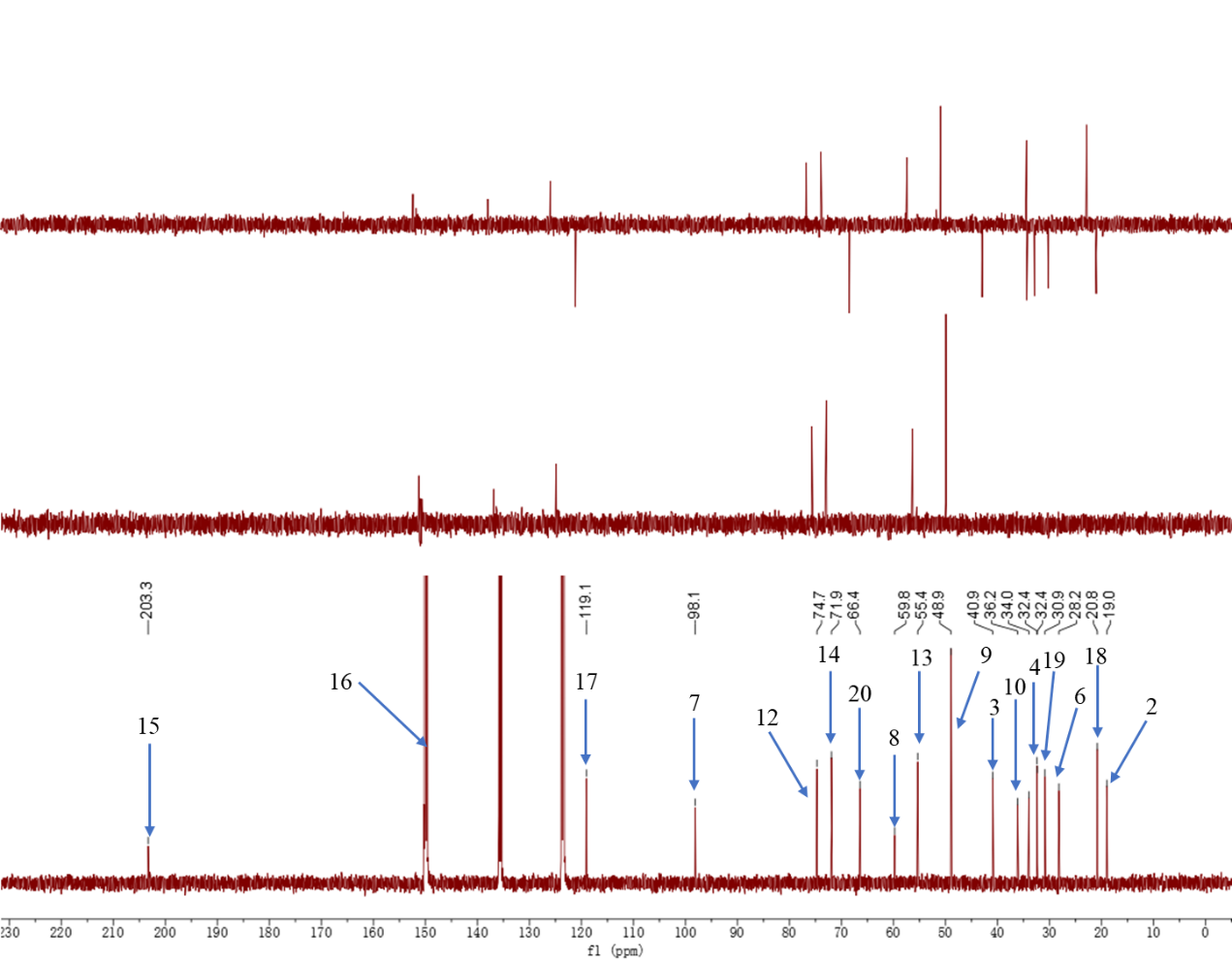
**

**Fig. S13** ^13^C NMR spectrum of silvaticusin B (**2**) (pyridine-*d*_5_, 125 MHz).

**
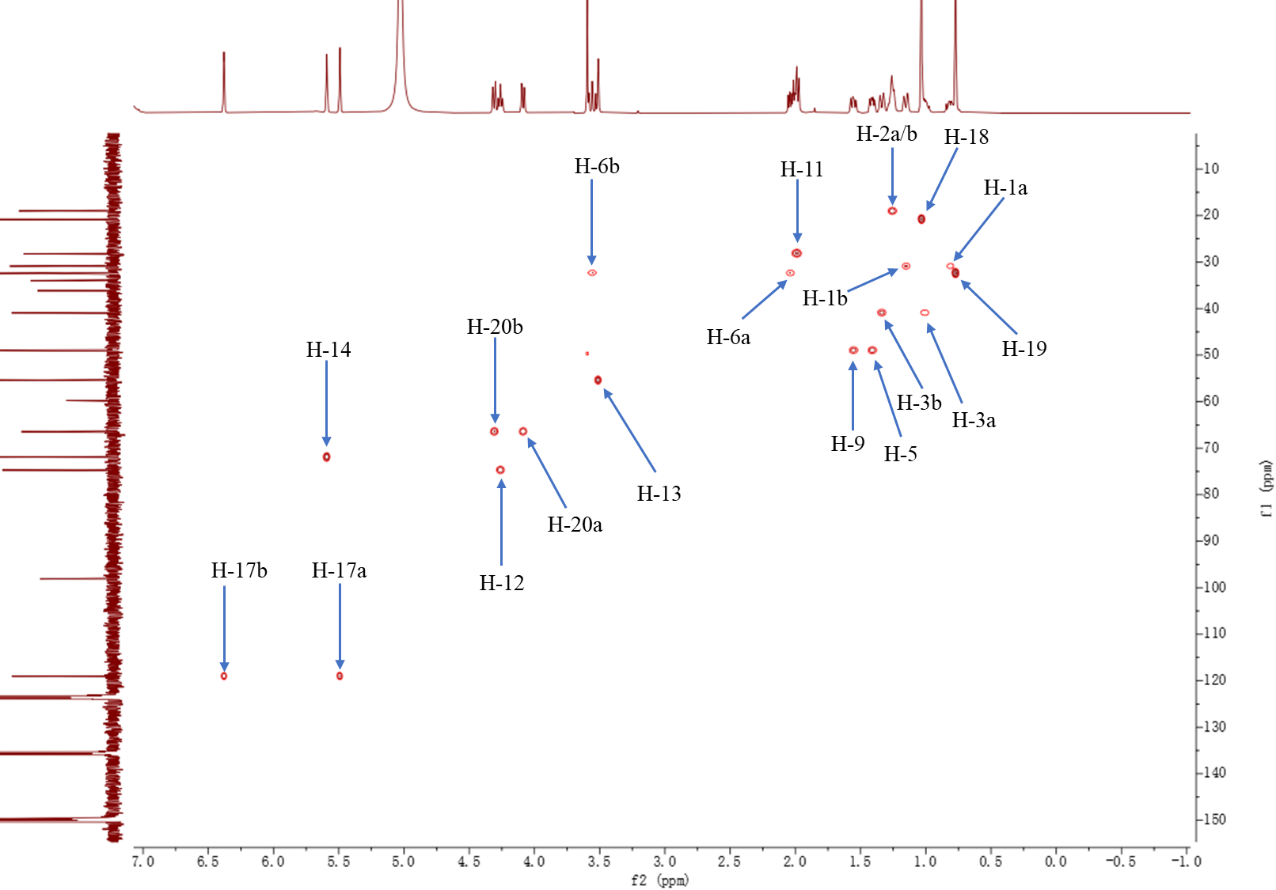
**

**Fig. S14** HSQC spectrum of silvaticusin B (**2**) (pyridine-*d*_5_, 500 MHz).

**
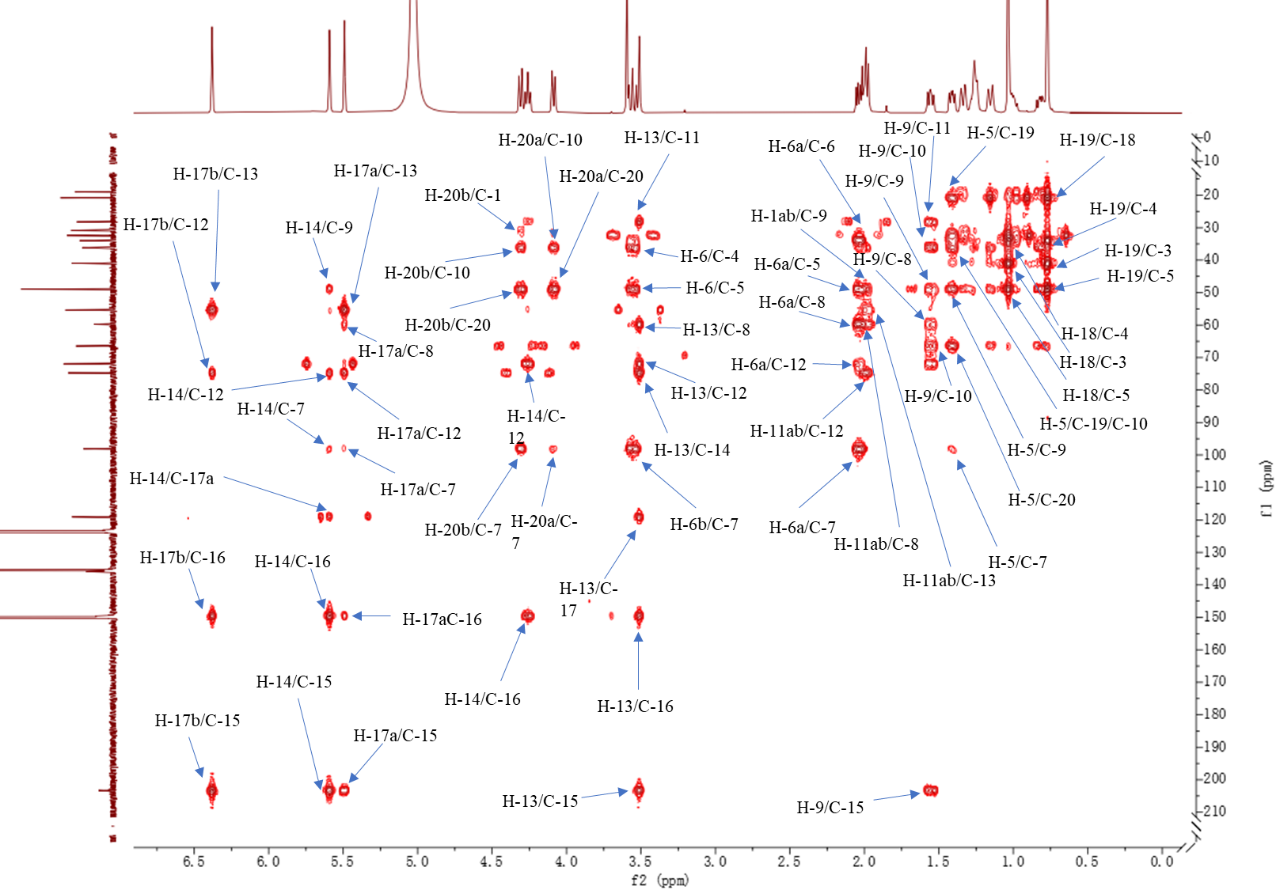
**

**Fig. S15** HMBC spectrum of silvaticusin B (**2**) (pyridine-*d*_5_, 500 MHz).

**
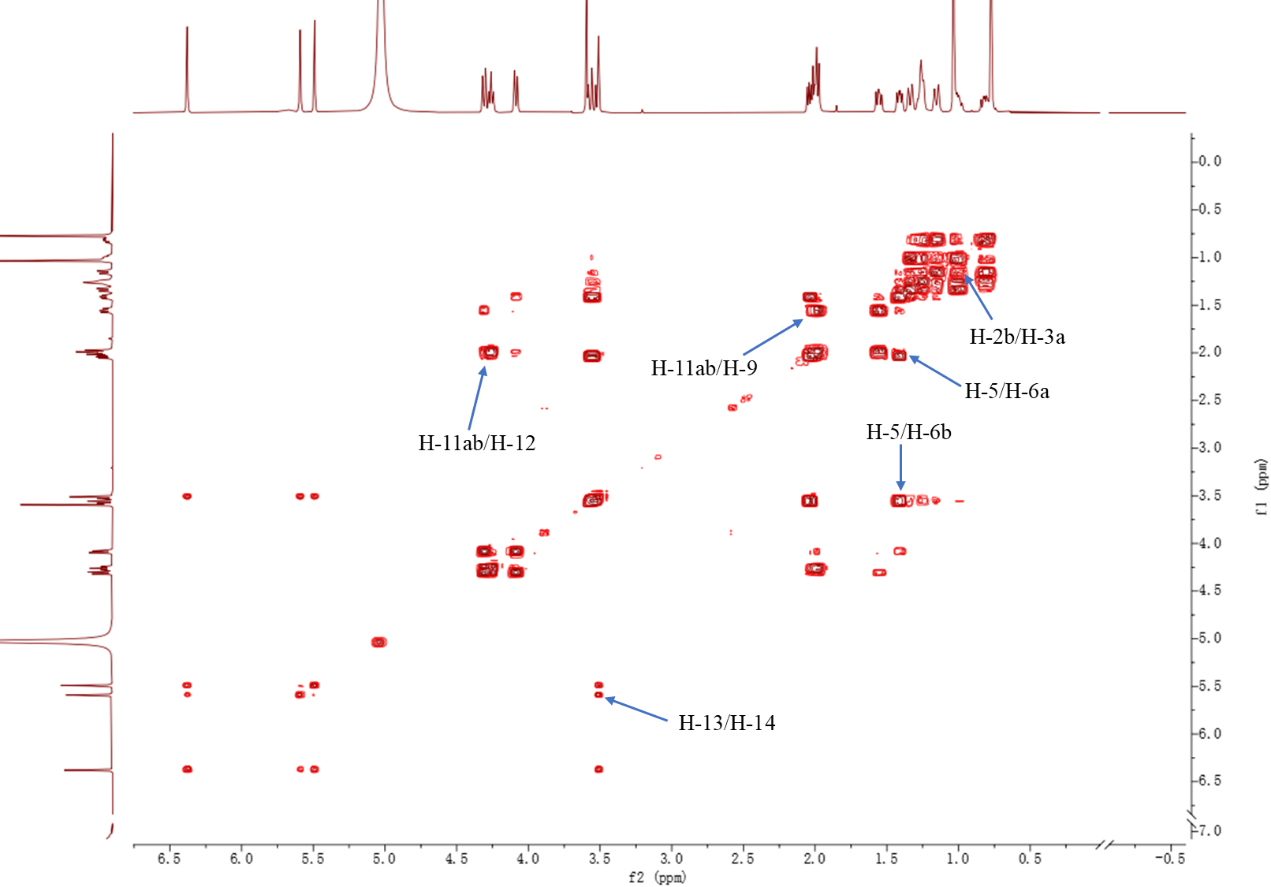
**

**Fig. S16** ^1^H-^1^H COSY spectrum of silvaticusin B (**2**) (pyridine-*d*_5_, 500 MHz).

**
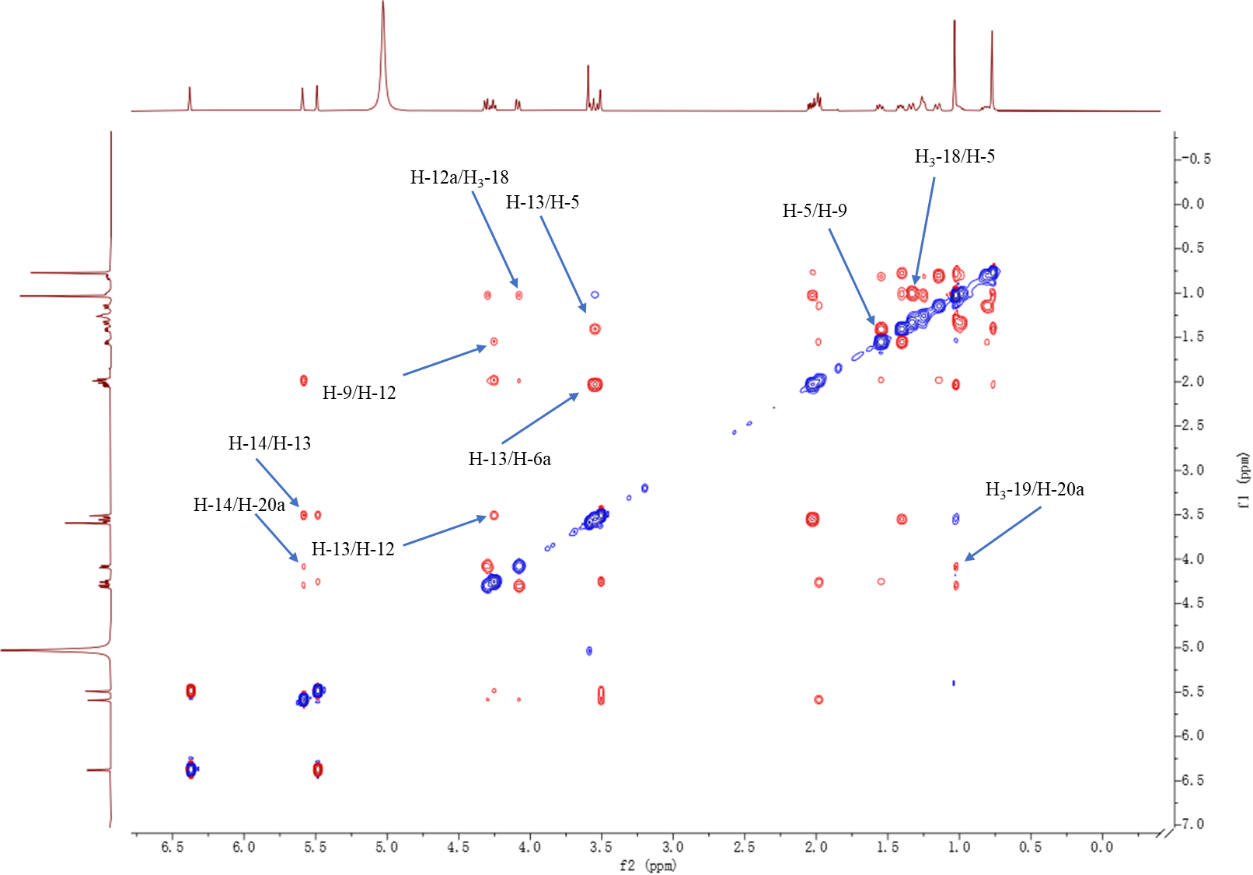
**

**Fig. S17** ROESY spectrum of silvaticusin B (**2**) (pyridine-*d*_5_, 500 MHz).


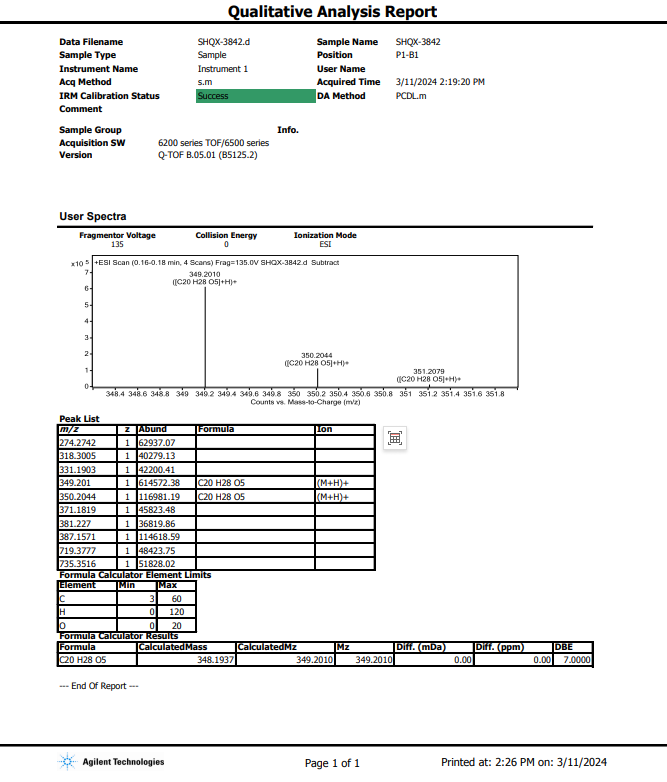


**Fig. S18** HRESIMS spectrum of silvaticusin B (**2**).


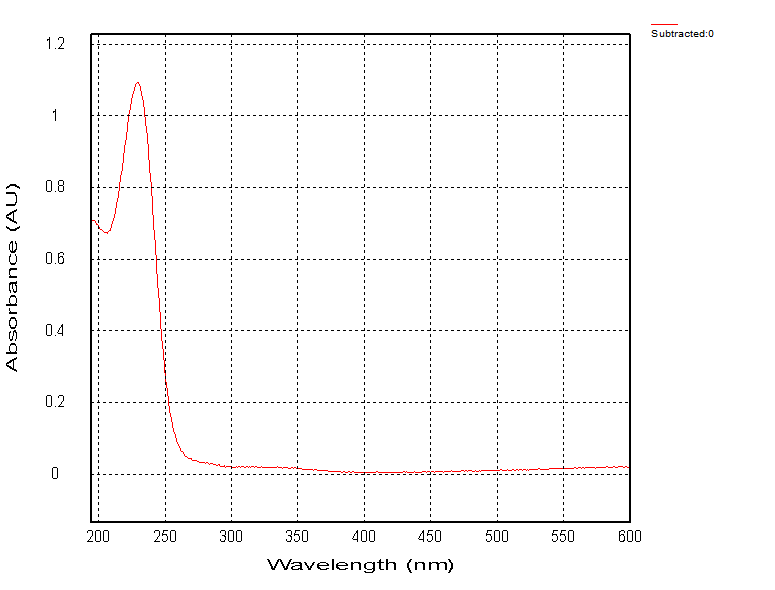


**Fig. S19** UV spectrum of silvaticusin B (**2**).


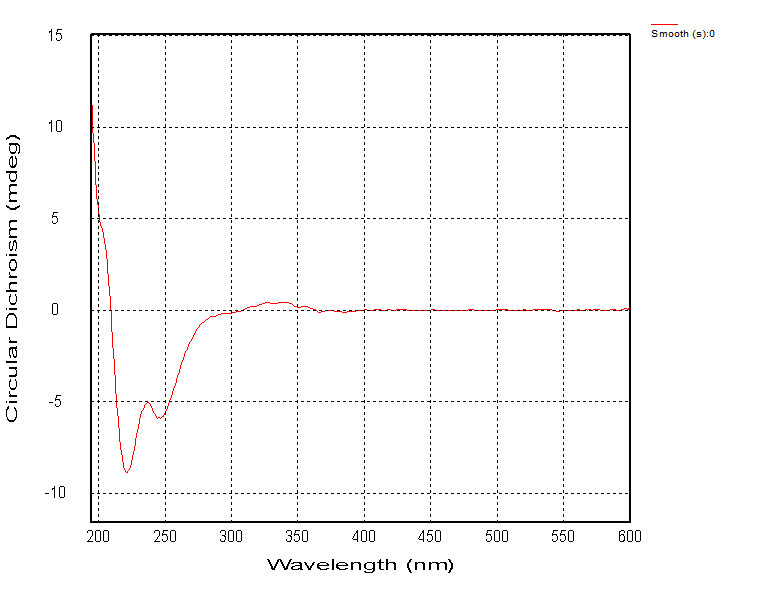


**Fig. S20** ECD spectrum of silvaticusin B (**2**).

**Fig. S21** IR spectrum of silvaticusin B (**2**).


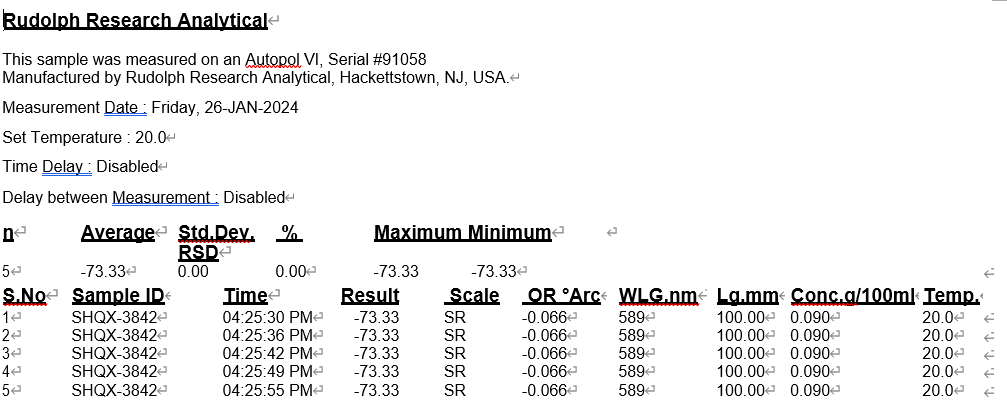


**Fig. S22** OR of silvaticusin B (**2**).

1. **NMR, MS, UV, ECD spectra, and OR of silvaticusin C (3)**


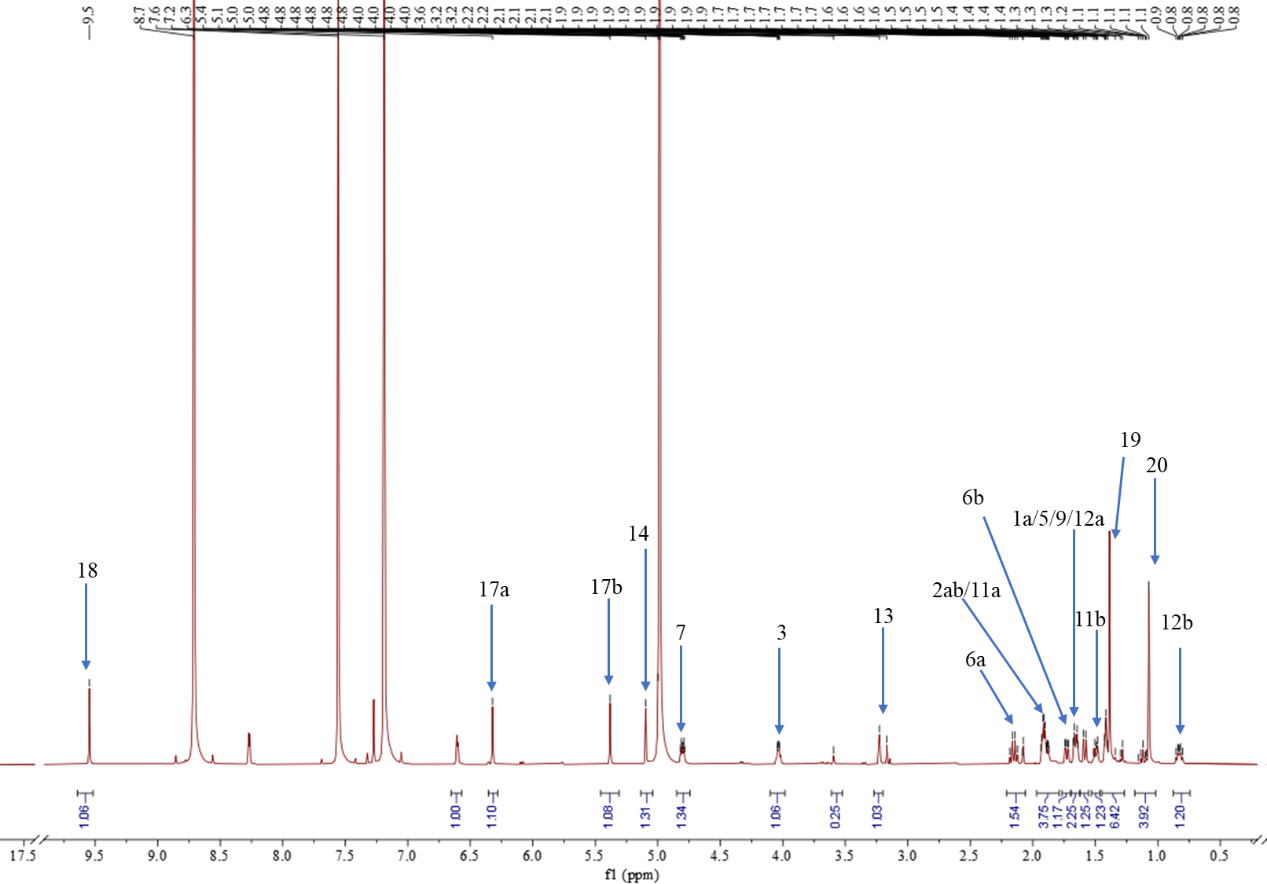


**Fig. S23** ^1^H NMR spectrum of silvaticusin C (**3**) (pyridine-*d*_5_, 600 MHz).


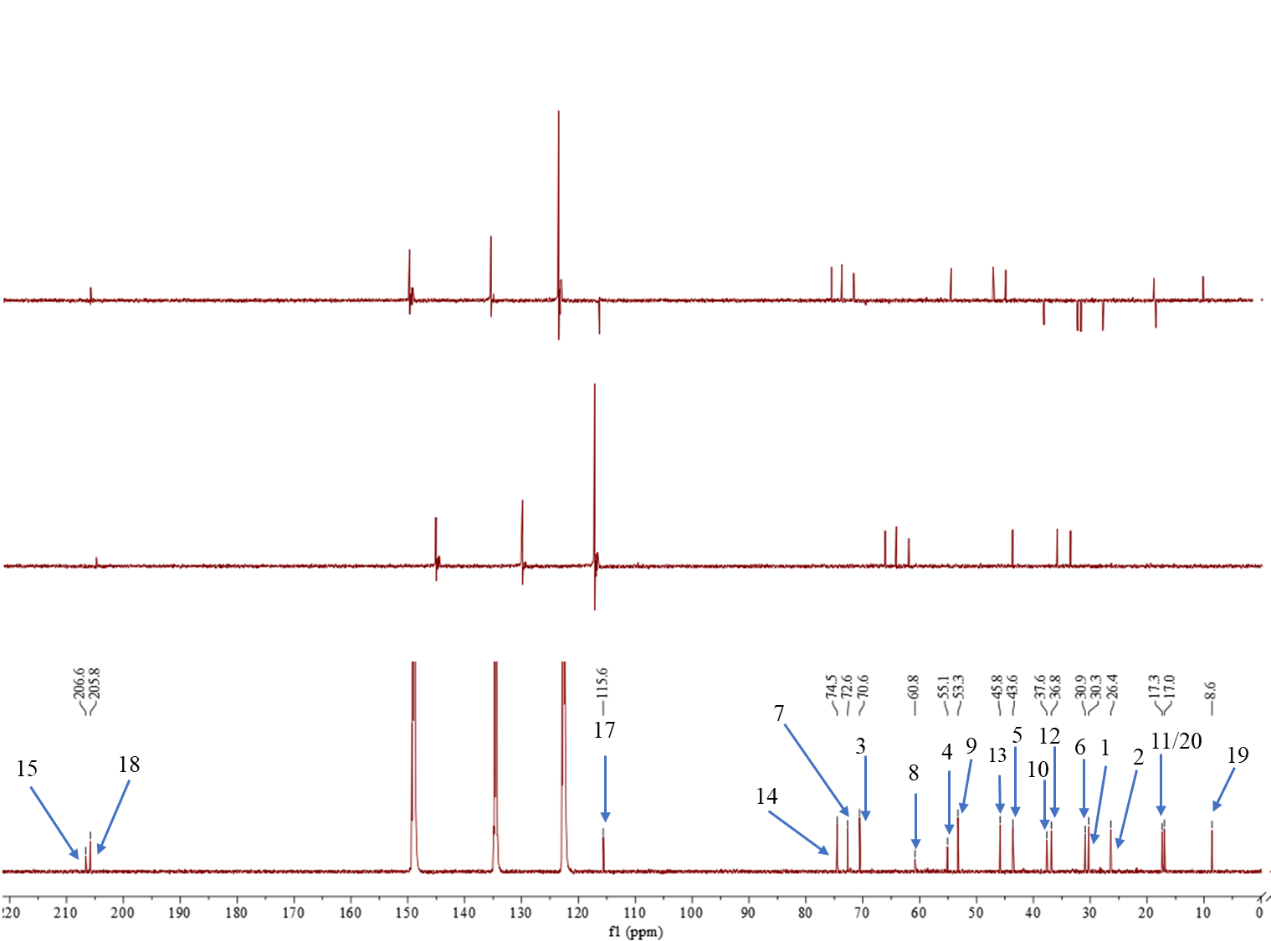


**Fig. S24** ^13^C NMR spectrum of silvaticusin C (**3**) (pyridine-*d*_5_, 150 MHz).


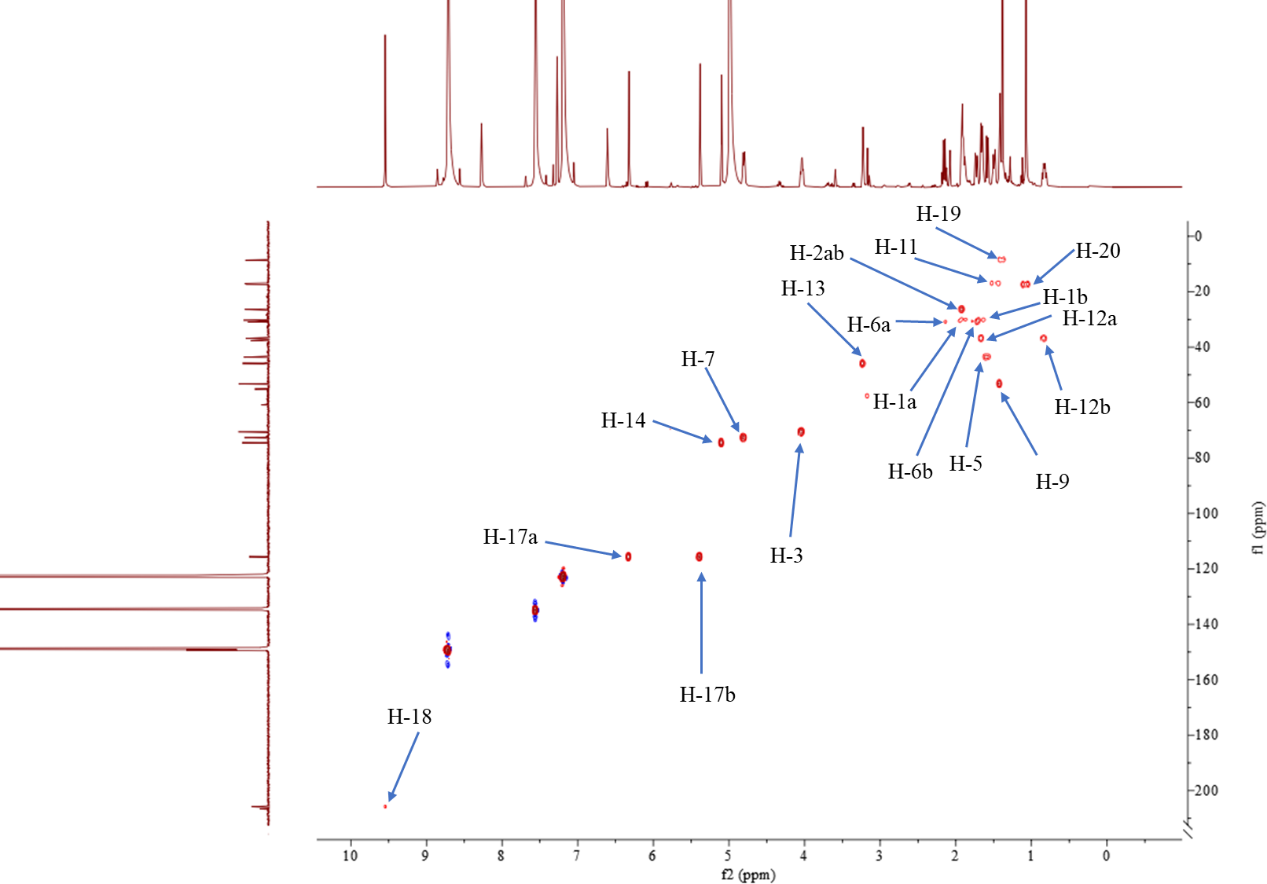


**Fig. S25** HSQC spectrum of silvaticusin C (**3**) (pyridine-*d*_5_, 600 MHz).


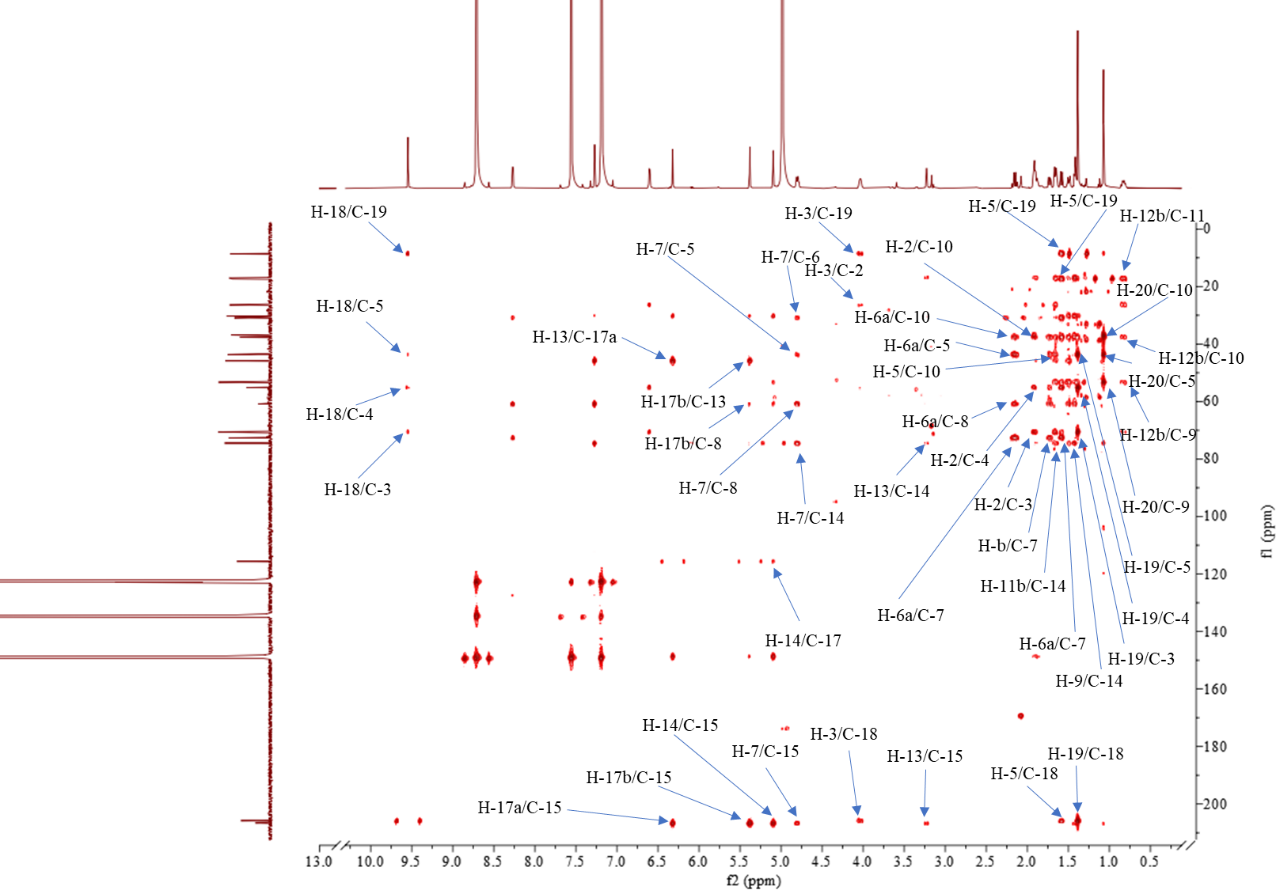


**Fig. S26** HMBC spectrum of silvaticusin C (**3**) (pyridine-*d*_5_, 600 MHz).


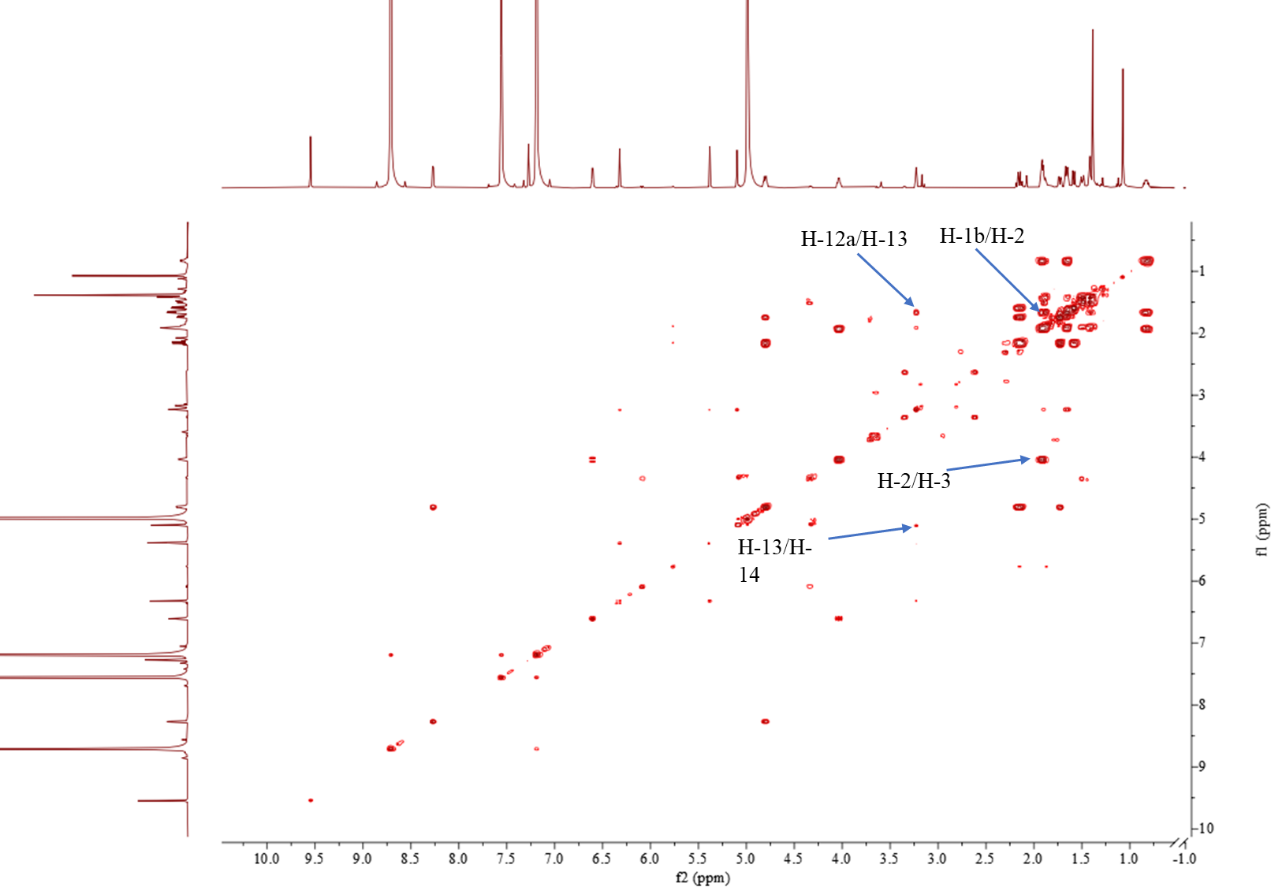


**Fig. S27** ^1^H-^1^H COSY spectrum of silvaticusin C (**3**) (pyridine-*d*_5_, 600 MHz).


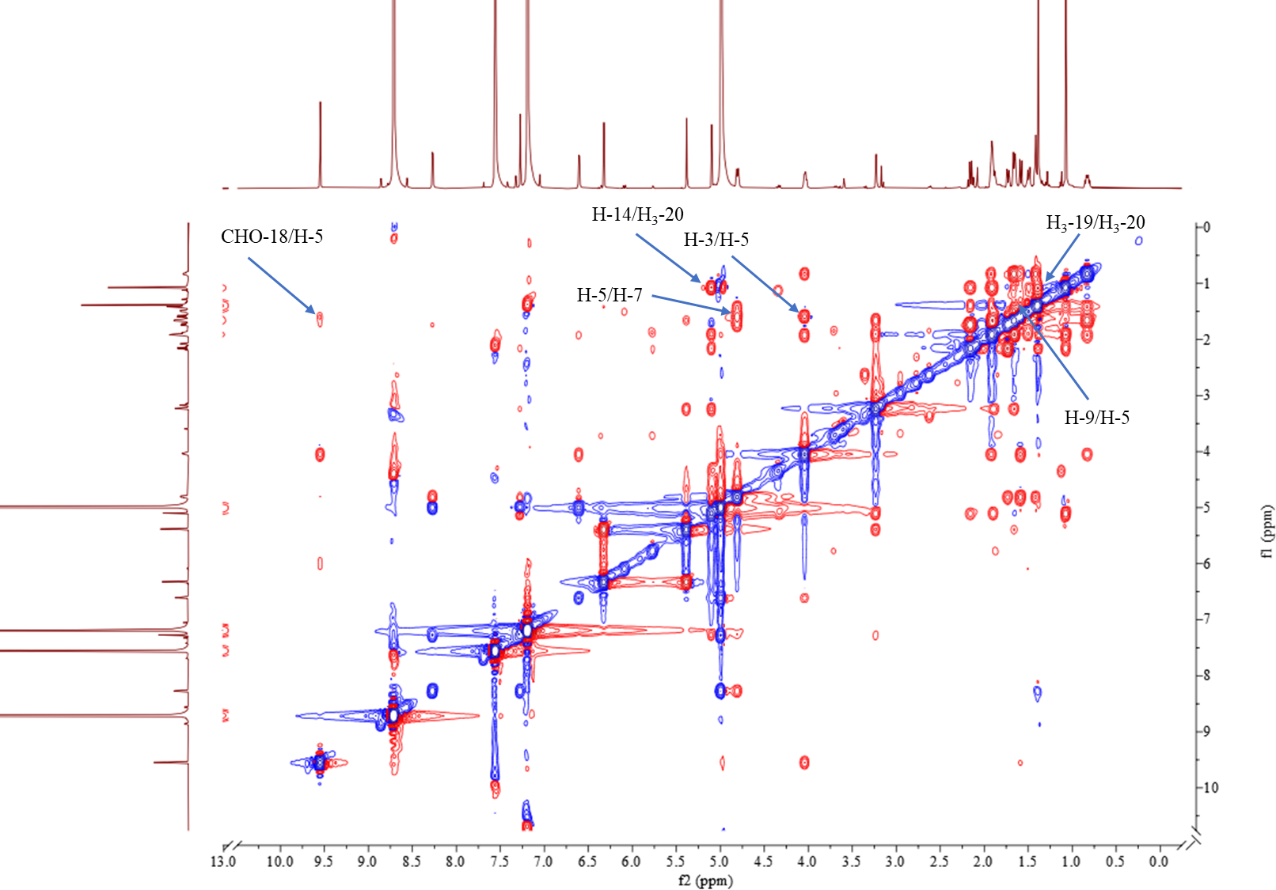


**Fig. S28** ROESY spectrum of silvaticusin C (**3**) (pyridine-*d*_5_, 600 MHz).


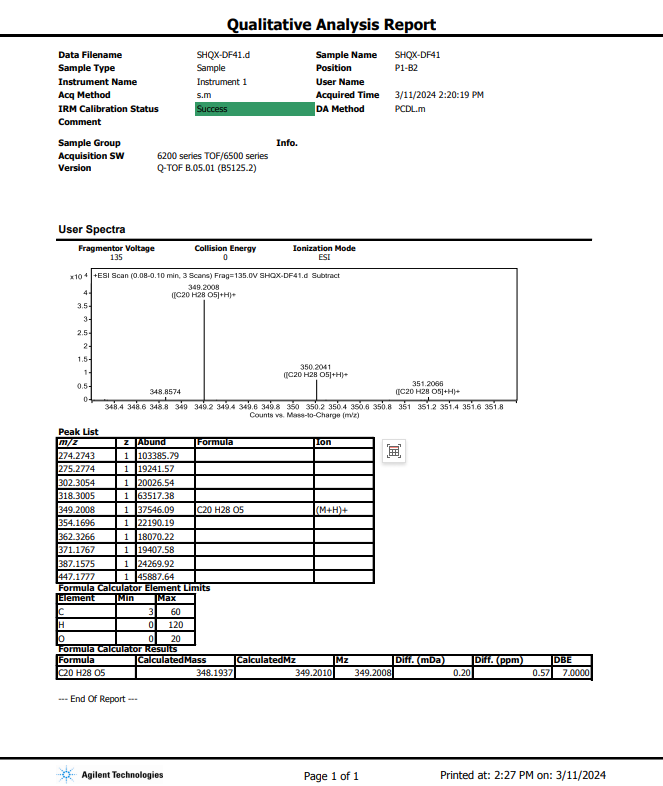


**Fig. S29** HRESIMS spectrum of silvaticusin C (**3**).


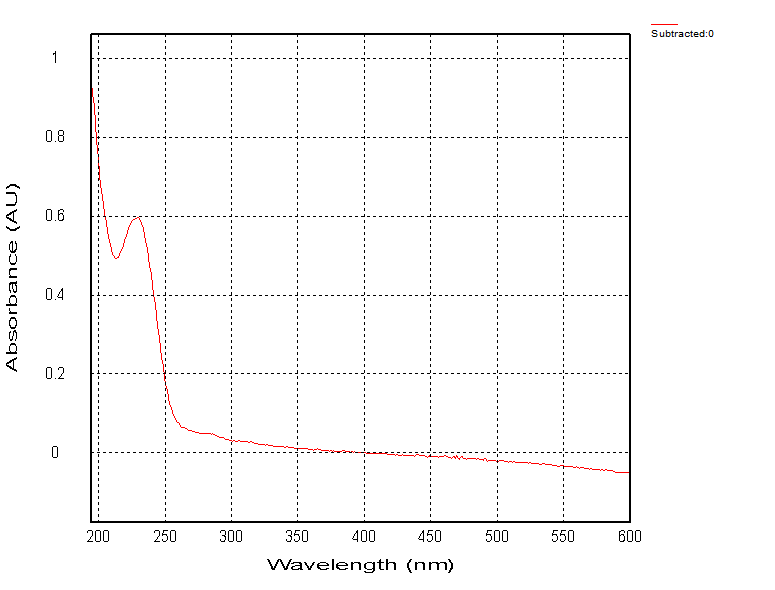


**Fig. S30** UV spectrum of silvaticusin C (**3**).


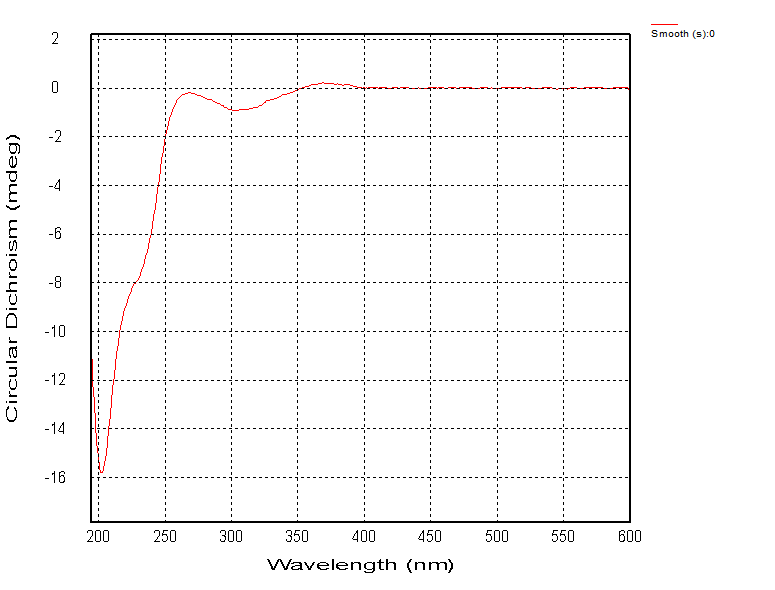


**Fig. S31** ECD spectrum of silvaticusin C (**3**).


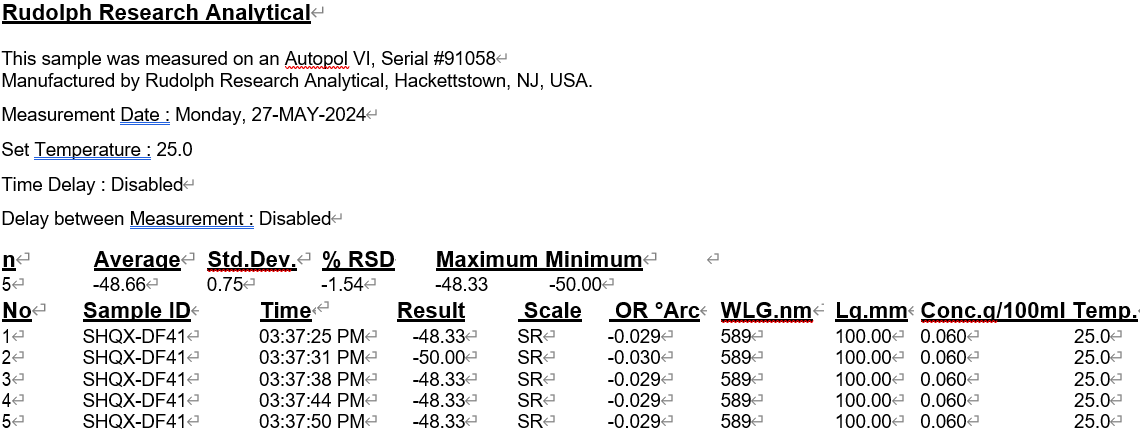


**Fig. S32** OR spectrum of silvaticusin C (**3**).

1. **NMR, MS, UV, ECD spectra, and OR of silvaticusin D (4)**

**
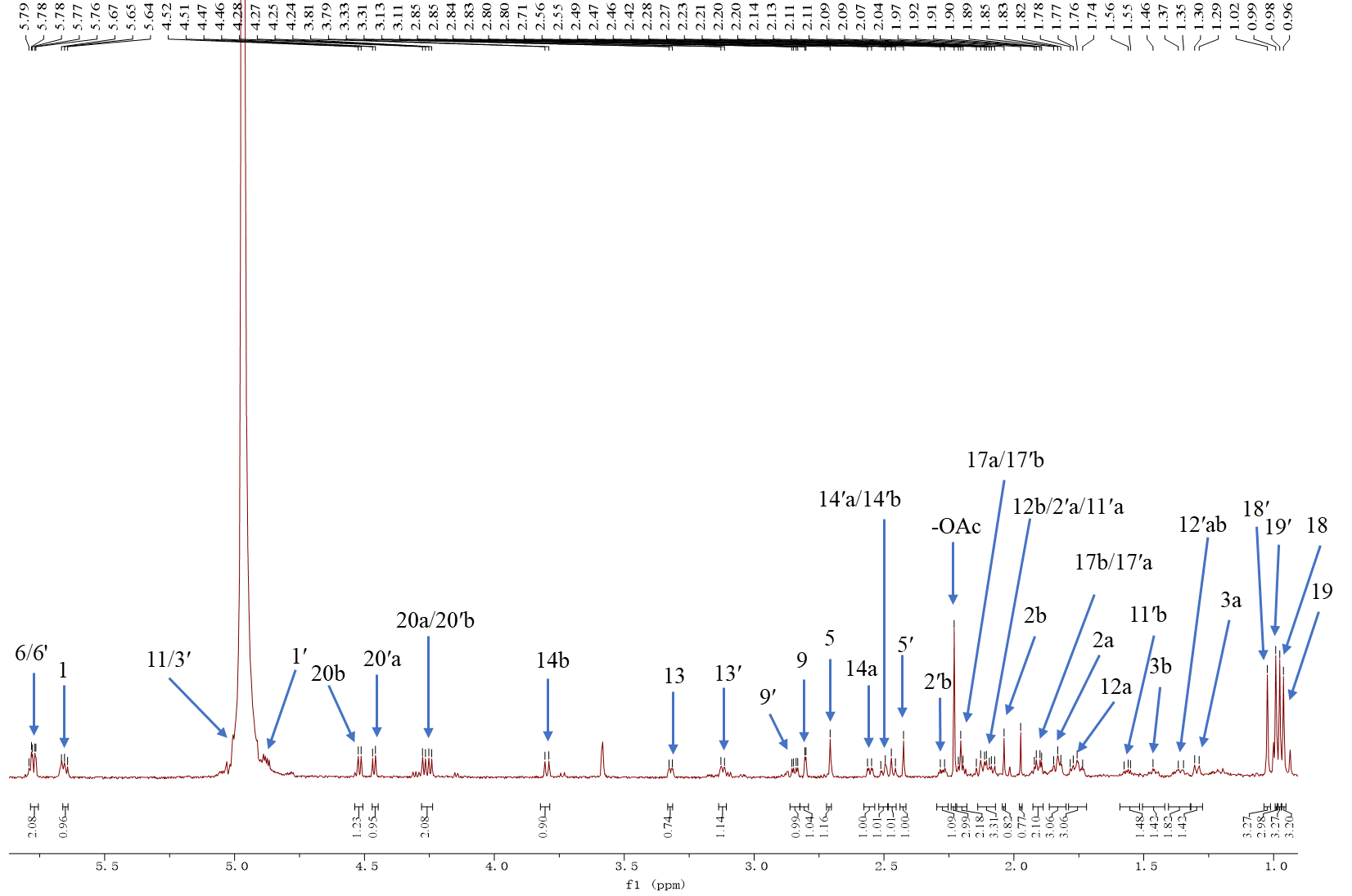
**

**Fig. S33** ^1^H NMR spectrum of silvaticusin D (**4**) (pyridine-*d*_5_, 800 MHz).

**
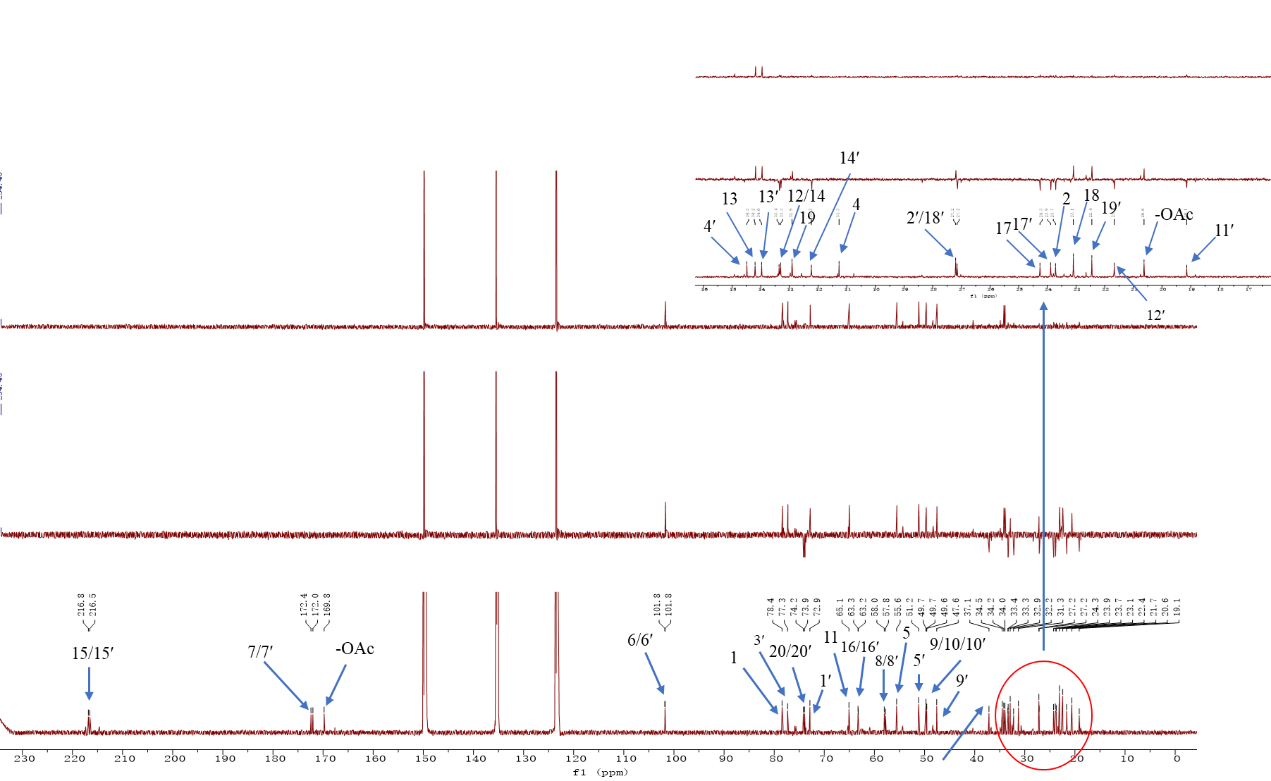
**

**Fig. S34** ^13^C NMR spectrum of silvaticusin D (**4**) (pyridine-*d*_5_, 200 MHz).


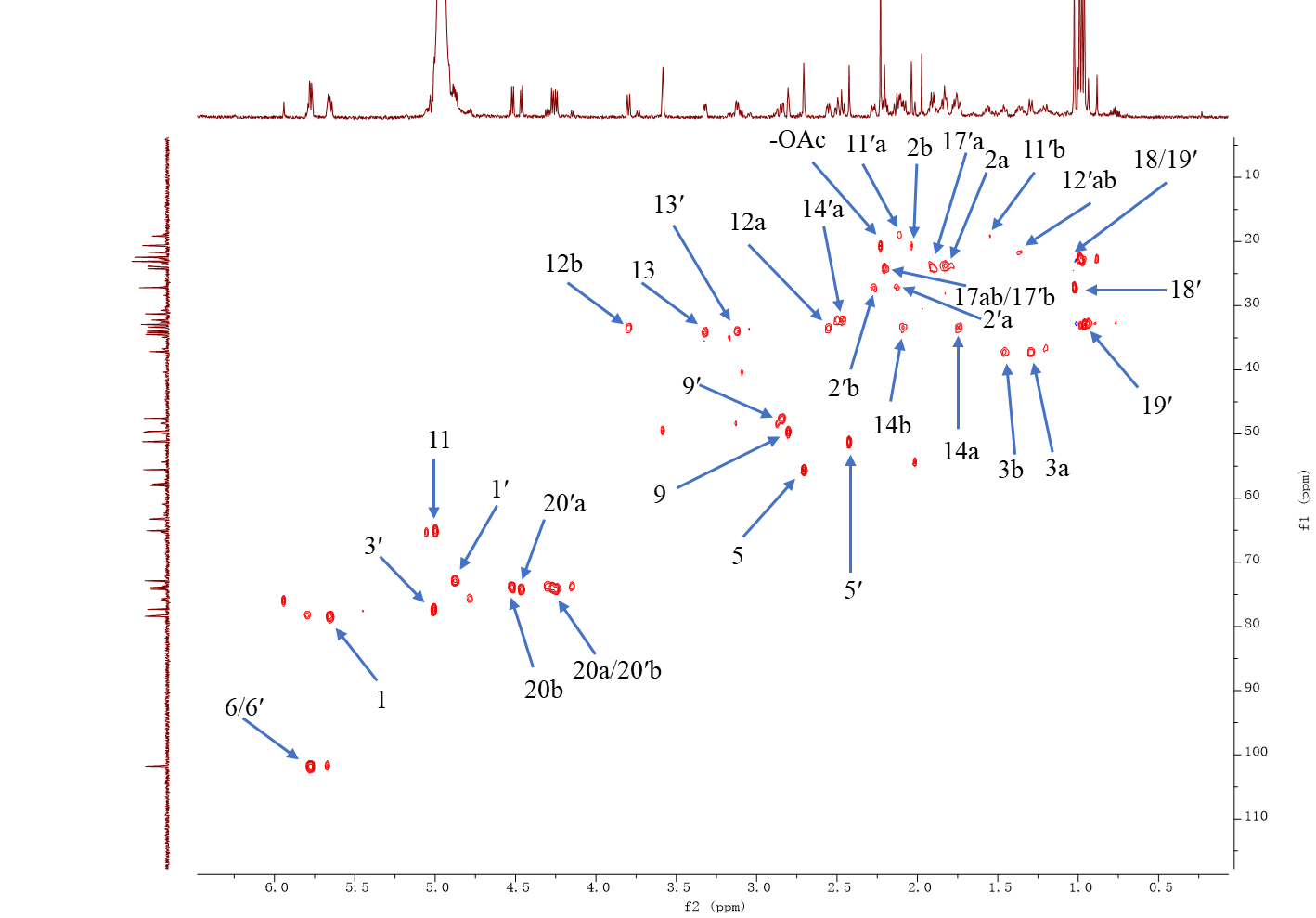


**Fig. S35** HSQC spectrum of silvaticusin D (**4**) (pyridine-*d*_5_, 800 MHz).


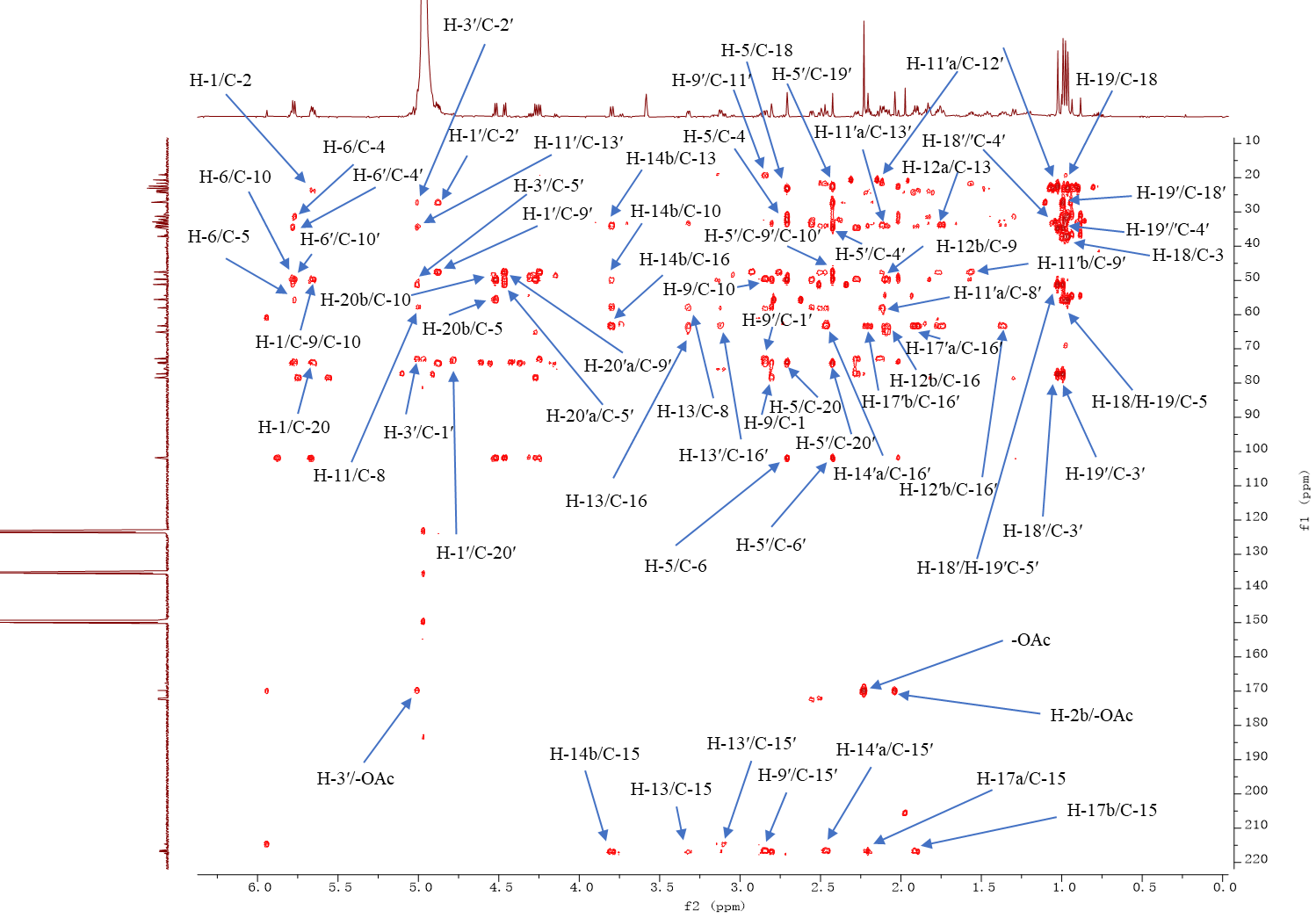


**Fig. S36** HMBC spectrum of silvaticusin D (**4**) (pyridine-*d*_5_, 800 MHz).


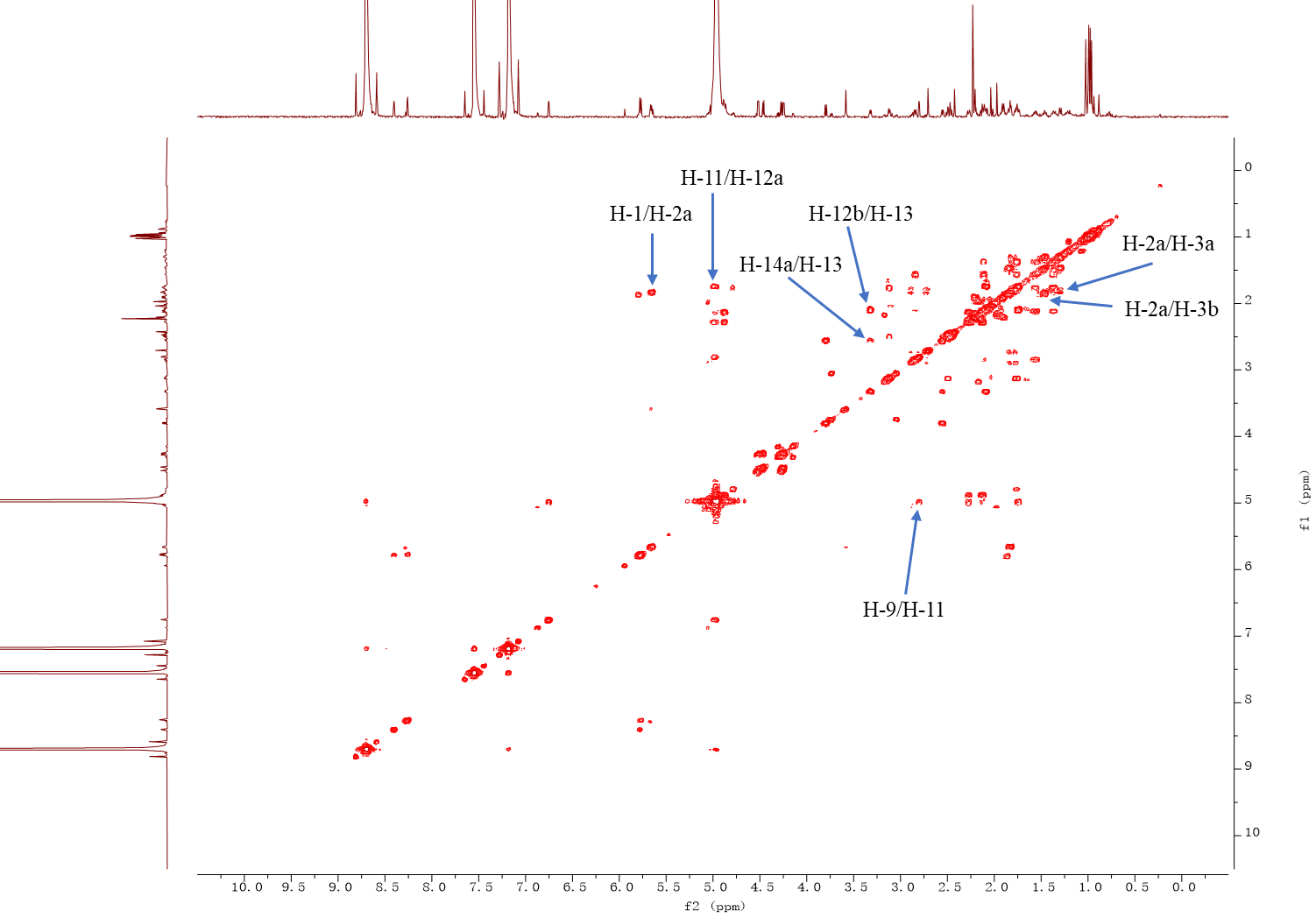


**Fig. S37** ^1^H–^1^H COSY spectrum of silvaticusin D (**4**) (pyridine-*d*_5_, 800 MHz).


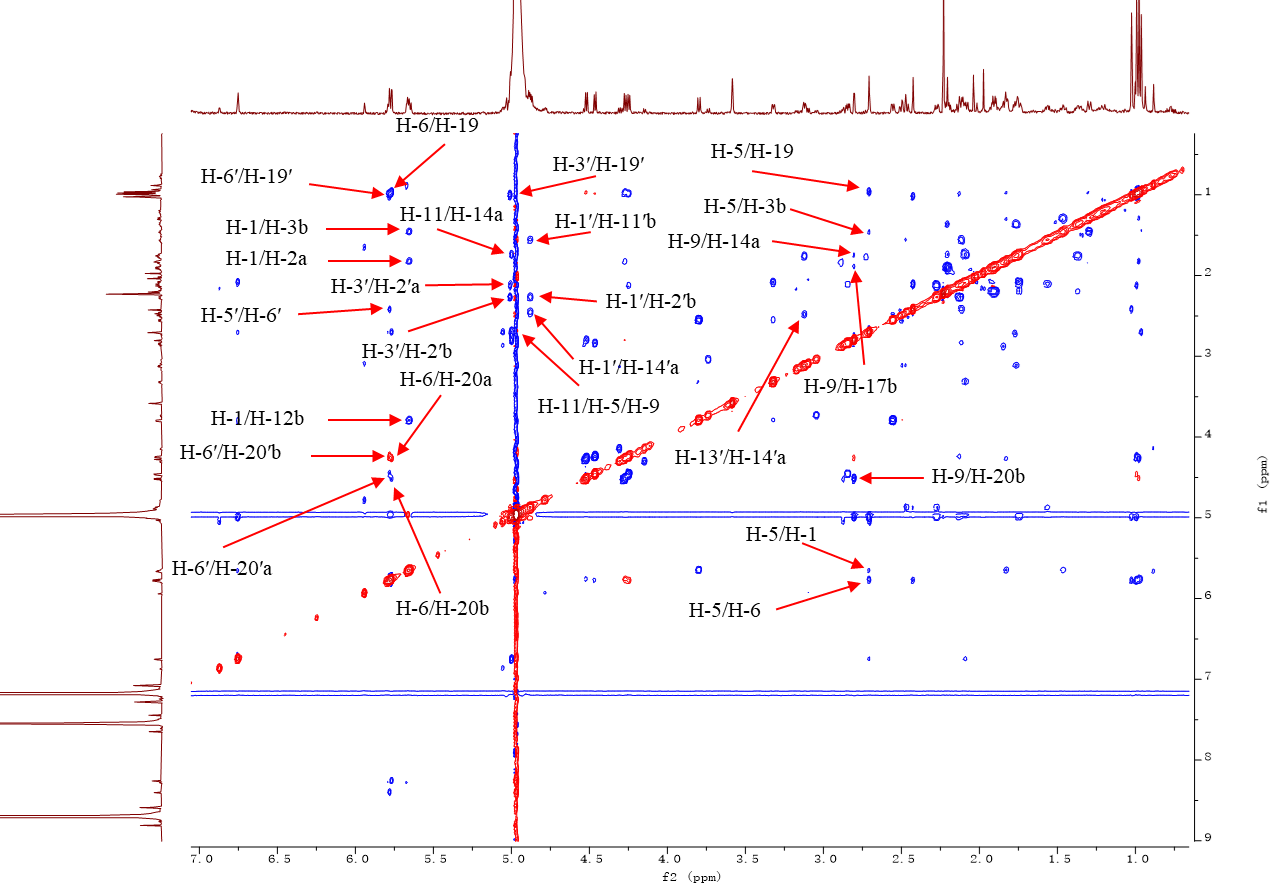


**Fig. S38** ROESY spectrum of silvaticusin D (**4**) (pyridine-*d*_5_, 800 MHz).


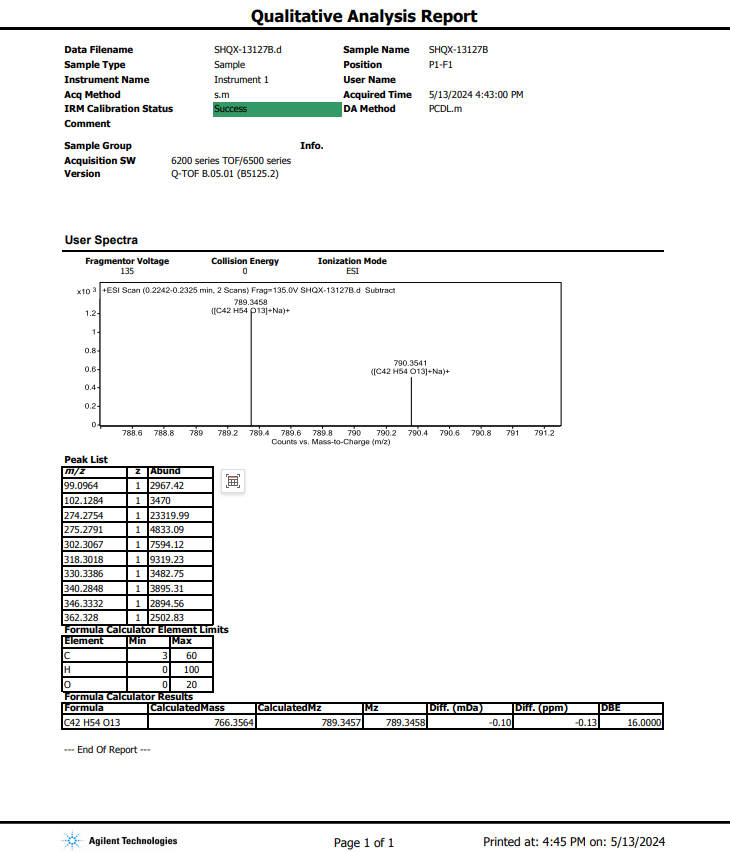


**Fig. S39** HRESIMS spectrum of silvaticusin D (**4**).


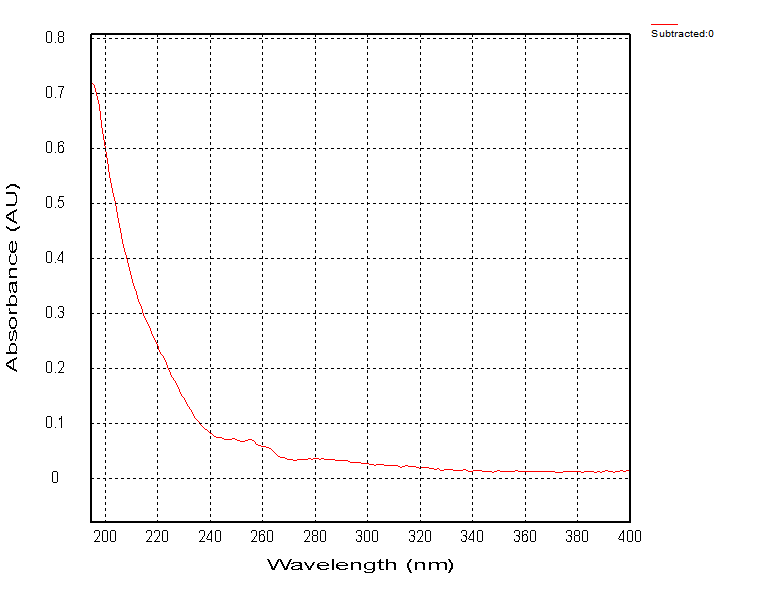


**Fig. S40** UV spectrum of silvaticusin D (**4**).


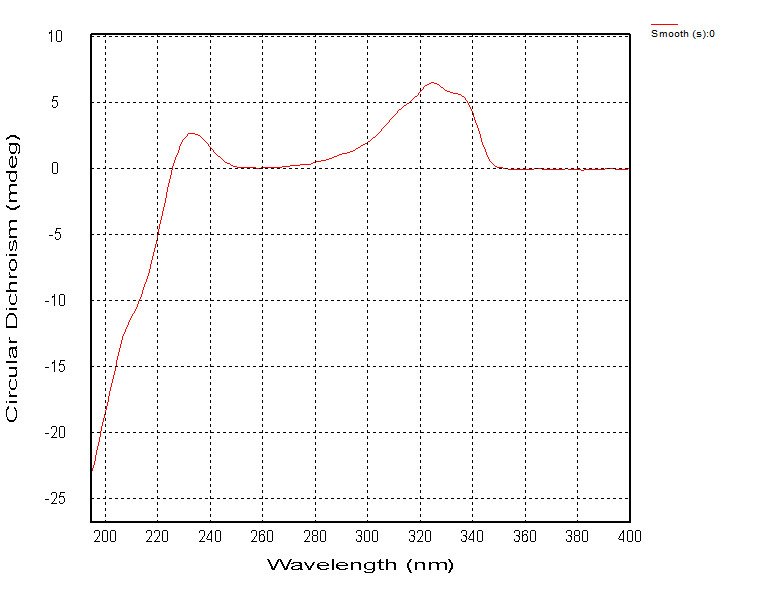


**Fig. S41** ECD spectrum of silvaticusin D (**4**).


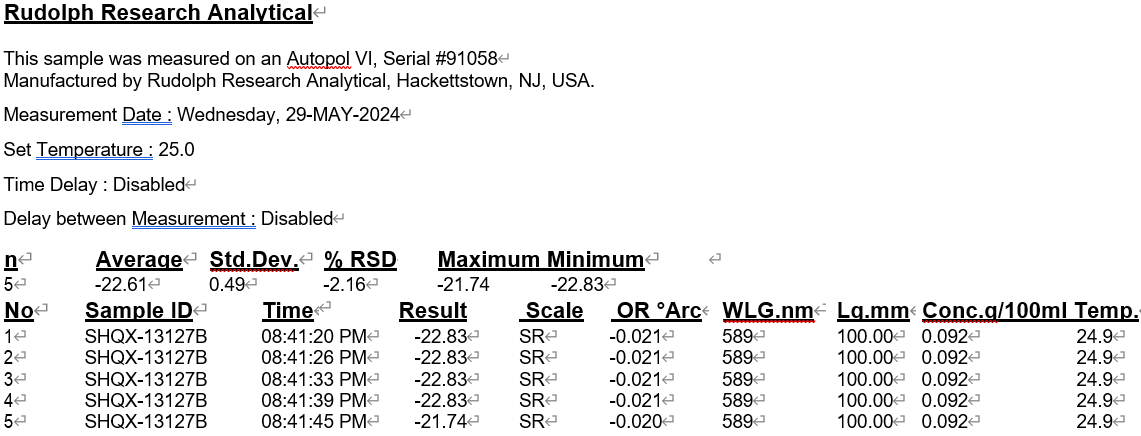


**Fig. S42** OR spectrum of silvaticusin D (**4**).

1. **Computational data of silvaticusin D (4)**

**9.1 Methods for NMR and ECD calculation**

Conformational searching of **4a** and **4b** (Fig. S43) were performed with the Crest code (version 3.0.1) using the default procedure [1]. Those conformers within an energy cut-off of 4 kcal/mol were subjected to DFT geometry optimizations at B3LYP-D3BJ/6-31G(d) level of theory in the gas phase. Frequency analysis of all optimized conformers were undertaken at the same level of theory to ensure that no imaginary frequency exists. More accurate energies of optimized conformers were evaluated at M06-2X-D3/6-311+G(2d,p) level of theory in the gas phase, and were then added to “thermal correction to Gibbs free energies” obtained by frequency analysis to get the Gibbs free energy of each conformer. Those two B3LYP geometries with RMSD below 0.15 Å and energy difference below 0.15 kcal were regarded as duplicate conformers, and the one with higher energy was removed. Subsequently, Room-temperature (298.15 K) equilibrium populations were calculated according to Boltzmann distribution law:

$$p_{i}= \frac{n_{i}}{\sum_{j} n_{j}}= \frac{e^{-\Delta G_{i}/RT}}{\sum_{j} e^{-\Delta G_{j}/RT}}$$

Where *P_i_* is the population of the *i^th^* conformer; *n_i_* the number of molecules in *i^th^* conformer; *ΔG* is the relative Gibbs free energy (kcal/mol); *T* is room temperature (298.15 K); *R* is the ideal gas constant (0.0019858995).

NMR shielding tensors of all dereplicated conformers were calculated with the GIAO method at mPW1PW91-SCRF/6-31+G(d,p) level (pyridine, IEFPCM solvent model). The shielding constants obtained were converted into chemical shifts by referencing to TMS at 0 ppm (*δ*_cal_ = *σ*_TMS_ – *σ*_cal_), where the *σ*_TMS_ was the shielding constant of TMS calculated at the same level. For **4a** and **4b**, the parameters *a* and *b* of the linear regression *δ*_cal_ = *aδ*_exp_ + *b*; the correlation coefficient, *R^2^*; the mean absolute error (*MAE*) defined as Σn |*δ*_cal_ – *δ*_exp_|/n; the corrected mean absolute error, *CMAE*, defined as Σn |*δ*_corr_ – *δ*_exp_|/n, where *δ*_corr_ = (*δ*_cal_ – b)/a, were calculated. Then, DP4+ probability analysis [2] were undertaken using the calculated NMR chemical shifts and scripts provided by Sarotti, *et al*, and DP4+ probabilities of **4a** and **4b** were obtained (Fig. S46).

For ECD calculation, those conformers with a population over 2% were subjected to TDDFT calculations at PBE0/6-311+G(2d,p) and CAM-B3LYP/6-311+G(2d,p) level of theory (MeOH, IEFPCM solvent model), and 36 excited states were calculated for each conformer. The coordinates for calculated ECD curves were generated using the Multiwfn software (version 3.8) [3].

The geometry optimization, single-point energy calculation, NMR shielding constant calculation, TDDFT ECD calculation were all completed in Gaussian 16 program [4].

[1] P. Pracht, S. Grimme, C. Bannwarth, F. Bohle, S. Ehlert, G. Feldmann, J. Gorges, M. Müller, T. Neudecker, C. Plett, S. Spicher, P. Steinbach, P.A. Wesołowski, F. Zeller, CREST—A program for the exploration of low-energy molecular chemical space. *J. Chem. Phys.* 21 (2024) 14110.

[2] N. Grimblat, M.M. Zanardi, A.M. Sarotti, Beyond DP4: an Improved Probability for the Stereochemical Assignment of Isomeric Compounds using Quantum Chemical Calculations of NMR Shifts, *J. Org. Chem.* 80 (2015), 12526–12534.

[3] T. Lu, F. Chen, Multiwfn: A multifunctional wavefunction analyzer, *J. Comput. Chem.* 33 (2012) 580–592.

[4] M.J. Frisch, G.W. Trucks, H.B. Schlegel, G.E. Scuseria, M.A. Robb, J.R. Cheeseman, G. Scalmani, V. Barone, B. Mennucci, G.A. Petersson, H. Nakatsuji, M. Caricato, X. Li, H.P. Hratchian, A.F. Izmaylov, J. Bloino, G. Zheng, J.L. Sonnenberg, M. Hada, M. Ehara, K. Toyota, R. Fukuda, J. Hasegawa, M. Ishida, T. Nakajima, Y. Honda, O. Kitao, H. Nakai, T. Vreven, J.A. Montgomery, J.E.P. Jr., F. Ogliaro, M. Bearpark, J.J. Heyd, E. Brothers, K.N. Kudin, V.N. Staroverov, T. Keith, R. Kobayashi, J. Normand, K. Raghavachari, A. Rendell, J.C. Burant, S.S. Iyengar, J. Tomasi, M. Cossi, N. Rega, J.M. Millam, M. Klene, J.E. Knox, J.B. Cross, V. Bakken, C. Adamo, J. Jaramillo, R. Gomperts, R.E. Stratmann, O. Yazyev, A.J. Austin, R. Cammi, C. Pomelli, J.W. Ochterski, R.L. Martin, K. Morokuma, V.G. Zakrzewski, G.A. Voth, P. Salvador, J.J. Dannenberg, S. Dapprich, A.D. Daniels, O. Farkas, J.B. Foresman, J.V. Ortiz, J. Cioslowski, D.J. Fox, Gaussian 16, Revision C.01; Gaussian, Inc., Wallingford CT: **2016**.

**9.2 General results for NMR calculation**

**Fig. S43** Chemical structures of (1*S**, 5*R**, 6*R**, 8*S**, 9*S**, 10*S**, 11*S**, 13*S**, 16*R**, 1'*S**, 3'*S**, 5'*R**, 6'*R**, 8'*S**, 9'*S**, 10'*S**, 13'*R**, 16'*R**)-**4** (**4a**) and (1*S**, 5*R**, 6*R**, 8*S**, 9*S**, 10*S**, 11*S**, 13*S**, 16*R**, 1'*S**, 3'*R**, 5'*R**, 6'*R**, 8'*S**, 9'*S**, 10'*S**, 13'*R**, 16'*R**)-**4** (**4b**).

**Table S1** Experimental and calculated ^13^C NMR chemical shifts of **4a** and **4b**.

| **No.** | ***δ_exptl._* (ppm)** | ***δ_calcd._* (ppm)** | | **No.** | ***δ_exptl._* (ppm)** | ***δ_calcd._* (ppm)** | |
| --- | --- | --- | --- | --- | --- | --- | --- |
|  |  | **4a** | **4b** |  |  | **4a** | **4b** |
| 1 | 78.5 | 75.9 | 77.6 | 4' | 34.5 | 37.9 | 38.7 |
| 2 | 23.7 | 25.7 | 26.1 | 5' | 51.2 | 51.9 | 53.6 |
| 3 | 37.1 | 37.8 | 37.9 | 6' | 101.8 | 103.5 | 103 |
| 4 | 31.3 | 33.4 | 34.1 | 7' | 172 | 171.1 | 170.9 |
| 5 | 55.6 | 52.3 | 53.4 | 8' | 58 | 62 | 62.1 |
| 6 | 101.8 | 103.2 | 104.1 | 9' | 47.6 | 50.5 | 49.5 |
| 7 | 172.4 | 170.5 | 170.2 | 10' | 49.7 | 52.3 | 52.1 |
| 8 | 57.8 | 62.1 | 61.5 | 11' | 19.1 | 22.2 | 22 |
| 9 | 49.7 | 53.3 | 54.8 | 12' | 21.6 | 23.7 | 24.3 |
| 10 | 49.6 | 53.3 | 51.9 | 13' | 33.9 | 37.8 | 39.1 |
| 11 | 65.1 | 65.7 | 65.5 | 14' | 32.2 | 34.8 | 35.9 |
| 12 | 33.4 | 36 | 36.5 | 15' | 216.5 | 218.1 | 220 |
| 13 | 34.2 | 37.5 | 38.9 | 16' | 63.2 | 65.8 | 66 |
| 14 | 33.3 | 34.8 | 36.1 | 17' | 23.9 | 26 | 27.6 |
| 15 | 216.8 | 218 | 220 | 18' | 27.3 | 27 | 27.8 |
| 16 | 63.3 | 66.3 | 67 | 19' | 22.5 | 23.9 | 18.5 |
| 17 | 24.3 | 26.4 | 27.5 | 20' | 74.3 | 75.3 | 75.1 |
| 18 | 32.9 | 31.6 | 31.7 | 21' | 169.8 | 168.7 | 169.5 |
| 19 | 23.1 | 24.1 | 24.3 | 22' | 20.6 | 22.1 | 22.1 |
| 20 | 73.9 | 74.8 | 74.7 |  |  |  |  |
| 1' | 72.9 | 72.5 | 74.5 | **R^2^** | **-** | **0.9989** | **0.9983** |
| 2' | 27.2 | 30.1 | 30.5 | **MAE** | **-** | **2.1** | **2.5** |
| 3' | 77.3 | 76.7 | 74.7 | **CMAE** | **-** | **1.4** | **1.7** |

**Table S2** Experimental and calculated ^1^H NMR chemical shifts of **4a** and **4b**.

| **No.** | ***δ_exptl._* (ppm)** | ***δ_calcd._* (ppm)** | | **No.** | ***δ_exptl._* (ppm)** | ***δ_calcd._* (ppm)** | |
| --- | --- | --- | --- | --- | --- | --- | --- |
|  |  | **4a** | **4b** |  |  | **4a** | **4a** |
| 1 | 5.65 | 4.41 | 4.4 | 2'a | 2.13 | 1.74 | 1.79 |
| 2b | 2.04 | 1.7 | 1.72 | 3' | 5.01 | 5.12 | 4.48 |
| 2a | 1.83 | 1.69 | 1.7 | 5' | 2.42 | 2 | 1.82 |
| 3a | 1.3 | 1.23 | 1.23 | 6' | 5.78 | 5.28 | 5.28 |
| 3b | 1.46 | 1.24 | 1.28 | 9' | 2.84 | 2.34 | 2.36 |
| 5 | 2.71 | 3.5 | 3.48 | 11'b | 1.56 | 1.47 | 1.28 |
| 6 | 5.77 | 5.28 | 5.29 | 11'a | 2.11 | 1.66 | 1.64 |
| 9 | 2.8 | 2.21 | 2.26 | 12'a | 1.36 | 1.3 | 1.24 |
| 11 | 5 | 3.98 | 3.96 | 12'b | 1.37 | 1.81 | 1.75 |
| 12a | 2.55 | 0.93 | 0.88 | 13' | 3.12 | 2.62 | 2.48 |
| 12b | 3.8 | 2.16 | 2.17 | 14a' | 2.47 | 2.2 | 1.95 |
| 13 | 3.32 | 2.66 | 2.5 | 14b' | 2.5 | 2.28 | 2.29 |
| 14a | 1.75 | 2.11 | 1.87 | 17'a | 1.91 | 1.86 | 1.67 |
| 14b | 2.09 | 2.24 | 2.25 | 17'b | 2.21 | 2.09 | 2.33 |
| 17b | 1.89 | 1.87 | 1.71 | 18' | 1.33 | 0.7 | 0.78 |
| 17a | 2.2 | 2.1 | 2.32 | 19' | 0.99 | 0.82 | 0.87 |
| 18 | 0.98 | 0.8 | 0.8 | 20'b | 4.25 | 4.01 | 4.06 |
| 19 | 0.96 | 0.78 | 0.78 | 20'a | 4.46 | 4.08 | 4.06 |
| 20a | 4.27 | 4 | 4.01 | 22' | 2.23 | 2.03 | 1.98 |
| 20b | 4.52 | 4.05 | 4.07 | **R^2^** | **-** | **0.8945** | **0.8969** |
| 1' | 4.88 | 4.71 | 4.34 | **MAE** | **-** | **0.41** | **0.45** |
| 2'b | 2.27 | 1.91 | 1.79 | **CMAE** | **-** | **0.33** | **0.33** |

**
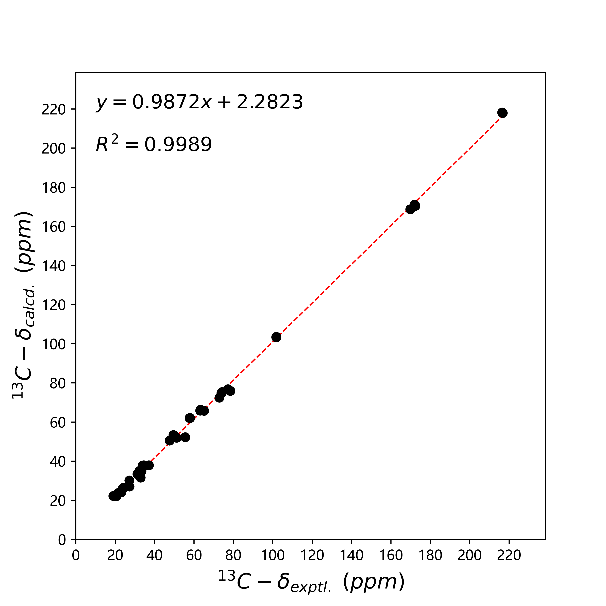

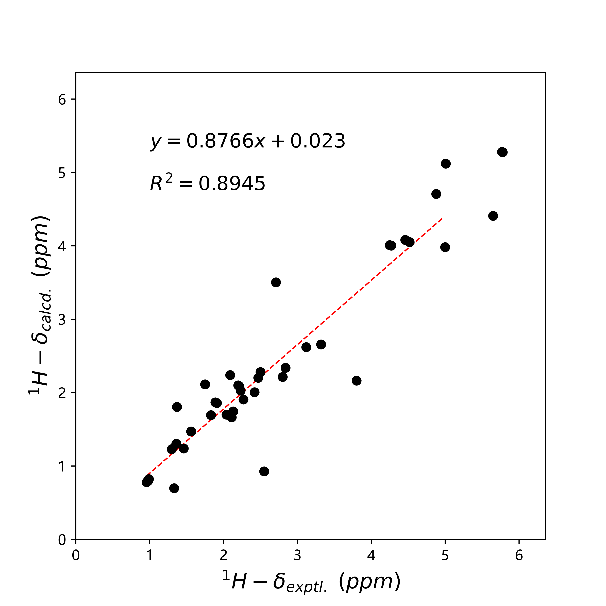
**

**Fig. S44** Linear regression analysis between the experimental and calculated NMR chemical shifts of **4a**.


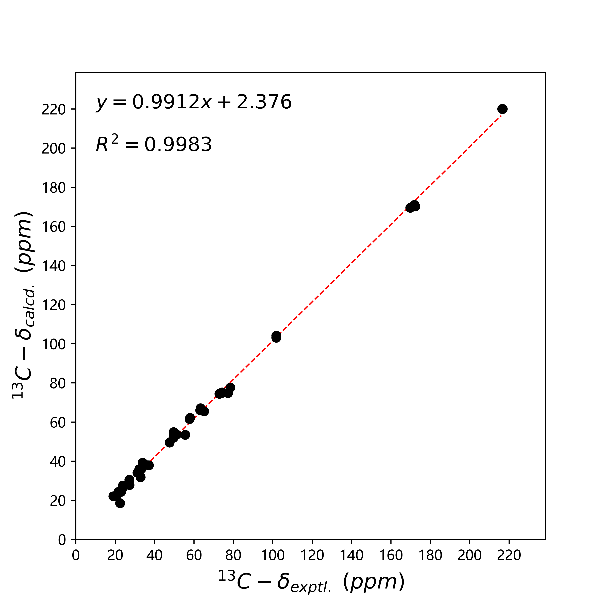

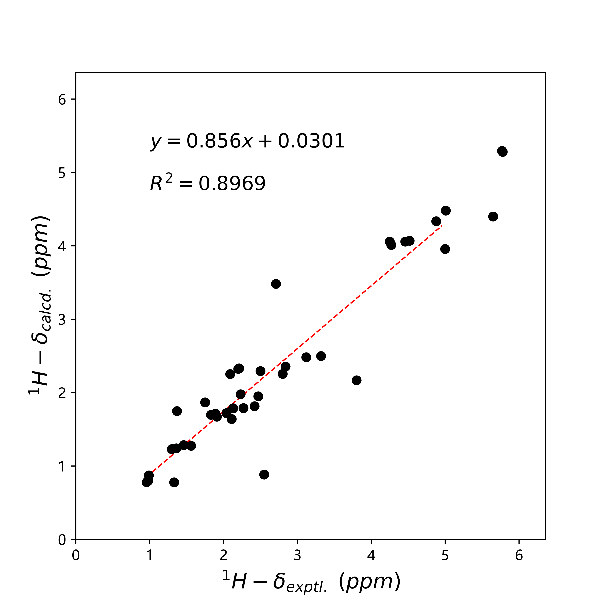


**Fig. S45** Linear regression analysis between the experimental and calculated NMR chemical shifts of **4b**.


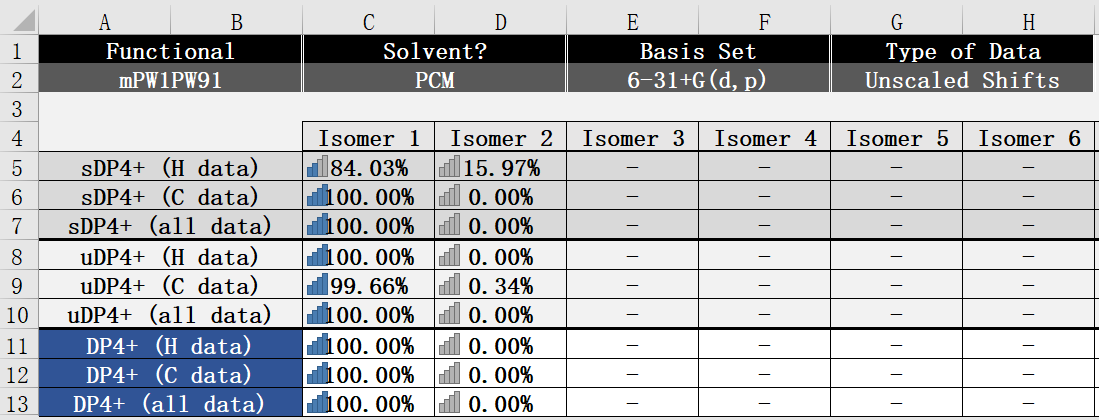


**Fig. S46** DP4+ analysis of **4a** and **4b**.

**9.3 Computational data of 4a**

**Table S3** Conformational analysis of the B3LYP-D3BJ/6-31G(d) optimized conformers of **4a** in the gas phase (T=298.15 K)

| Conformer | E (Hartree)*^a^* | C (Hartree)*^b^* | G (kcal/mol)*^c^* | *Δ*G (kcal/mol)*^d^* | Population*^e^* |
| --- | --- | --- | --- | --- | --- |
| **4a-1** | -2610.714755 | 0.836925 | -1637698.338275 | 0.0 | 64.58% |
| **4a-2** | -2610.715307 | 0.838044 | -1637697.982765 | 0.35551 | 35.42% |

*^a^*Electronic energy obtained at M06-2X-D3/6-311+G(2d,p) level of theory; *^b^*Thermal correction to Gibbs free energy obtained at B3LYP-D3BJ/6-31G(d) level of theory; *^c^*Gibbs free energy (E + C); *^d^*The relative Gibbs free energy; *^e^*The Boltzmann distribution of each conformer.

**Table S4** Atomic coordinates (Å) of **4a-1** obtained at the B3LYP-D3BJ/6-31G(d) level of theory in the gas phase.

| C | -5.215283 | 1.826997 | 0.691743 | O | 1.264506 | -2.697893 | 0.741388 |
| --- | --- | --- | --- | --- | --- | --- | --- |
| C | -6.360408 | 2.789802 | 0.939062 | H | 5.312635 | -3.452780 | -1.893812 |
| C | -7.528420 | 2.027458 | 1.565185 | H | -5.649357 | -2.465704 | 0.608965 |
| C | -8.098180 | 0.948391 | 0.622183 | H | -6.582866 | -2.464360 | -1.858013 |
| C | -6.954660 | -0.056237 | 0.259392 | H | 7.785403 | 1.765922 | 1.289744 |
| C | -7.253836 | -0.838130 | -1.019082 | O | 6.327683 | 2.246368 | -0.093407 |
| C | -3.104471 | 1.867759 | -0.451422 | H | 1.843504 | 0.914976 | -3.062708 |
| C | -3.008013 | 0.400023 | -0.075395 | H | 1.781854 | -0.829518 | -2.938614 |
| C | -4.359055 | -0.416304 | 0.086514 | H | 5.614840 | 2.030789 | 2.453143 |
| C | -5.563979 | 0.560388 | -0.095785 | H | 6.187388 | 0.382799 | 2.683886 |
| C | -4.285880 | -1.277190 | 1.370640 | H | 1.968440 | 1.671969 | -0.178070 |
| C | -2.944664 | -2.047641 | 1.448429 | H | 0.592380 | 1.038209 | 0.717227 |
| C | -1.693416 | -1.201202 | 1.096122 | H | 9.157356 | 0.821235 | -0.613548 |
| C | -2.098867 | 0.278338 | 1.173447 | H | 7.809505 | 0.977344 | -1.752083 |
| C | -2.212188 | -0.399084 | -1.121340 | H | 8.578039 | -0.595079 | -1.508291 |
| C | -1.289602 | -1.368151 | -0.386081 | H | -2.858415 | -2.451104 | 2.461712 |
| C | -1.253876 | -2.759027 | -1.071759 | H | -3.026931 | -2.914918 | 0.783687 |
| C | -9.184138 | 0.159487 | 1.376592 | H | 5.353816 | -1.802785 | 2.164644 |
| C | -8.769369 | 1.633732 | -0.587995 | H | 3.941672 | -2.486126 | 1.344949 |
| C | -5.727960 | 0.831224 | -1.613525 | H | -2.590288 | 0.549155 | 2.110316 |
| C | 4.553558 | 0.576543 | 1.286981 | H | -1.245748 | 0.946646 | 1.049197 |
| C | 5.825838 | 1.090810 | 1.933691 | H | -7.190478 | 1.549706 | 2.496378 |
| C | 6.891073 | 1.307065 | 0.864796 | H | -8.328065 | 2.723336 | 1.845172 |
| C | 7.276240 | 0.002919 | 0.136687 | H | -4.369736 | -0.623102 | 2.246925 |
| C | 5.999223 | -0.641627 | -0.493760 | H | -6.011031 | 3.591350 | 1.598510 |
| C | 6.169454 | -2.135543 | -0.769679 | H | -6.657092 | 3.264096 | -0.001388 |
| C | 2.426265 | -0.260197 | 1.987751 | H | 3.632649 | 1.123559 | -1.506915 |
| C | 2.162225 | -0.437912 | 0.503300 | H | 7.203460 | -2.444983 | -0.958527 |
| C | 3.397667 | -0.756102 | -0.439896 | H | -0.866596 | -1.438303 | 1.767568 |
| C | 4.713700 | -0.650290 | 0.386174 | H | -4.808408 | 1.530501 | 1.667823 |
| C | 3.321164 | 0.092841 | -1.718040 | H | -0.304249 | -1.914793 | -2.858973 |
| C | 1.910636 | 0.085612 | -2.350684 | H | 0.786714 | -3.045006 | -1.994178 |
| C | 0.760911 | 0.210018 | -1.310600 | H | 4.130127 | 1.393234 | 0.694654 |
| C | 1.358981 | 0.780143 | -0.015054 | H | -8.318910 | -0.988792 | -1.218657 |
| C | 1.193052 | -1.597993 | 0.236936 | H | -1.004548 | -3.538438 | -0.349356 |
| C | 0.227498 | -1.171983 | -0.868104 | H | -2.145892 | -3.037660 | -1.638823 |
| C | -0.021325 | -2.317270 | -1.884398 | H | 8.445602 | -1.772104 | 0.667919 |
| C | 8.262907 | 0.323437 | -1.001656 | H | 8.848633 | -0.328434 | 1.595164 |
| C | 8.018389 | -0.892405 | 1.154895 | H | 7.386640 | -1.249728 | 1.968521 |
| C | 4.890954 | -1.964985 | 1.185866 | H | -0.038547 | 0.839232 | -1.705424 |
| C | 7.059601 | 3.344332 | -0.414946 | H | -4.806721 | 0.657732 | -2.177697 |
| C | 6.316836 | 4.185959 | -1.423060 | H | -6.065951 | 1.850859 | -1.820613 |
| O | -6.731348 | -0.087412 | -2.084097 | H | -9.517706 | 2.347556 | -0.224592 |
| O | 5.757234 | -2.802841 | 0.402460 | H | -8.070869 | 2.181579 | -1.222288 |
| O | -6.630929 | -2.127654 | -0.948414 | H | -9.289673 | 0.914870 | -1.226647 |
| H | 4.031903 | -0.305818 | -2.445038 | H | -10.015668 | 0.817005 | 1.655581 |
| O | -5.352800 | -2.205889 | 1.502494 | H | -9.593745 | -0.649055 | 0.760039 |
| O | -4.183134 | 2.557356 | -0.017247 | H | -8.782910 | -0.289194 | 2.292232 |
| H | -4.412183 | -1.127055 | -0.743204 | O | -2.215538 | 2.445224 | -1.034351 |
| H | 3.315466 | -1.797743 | -0.757152 | O | 1.589608 | -0.487406 | 2.830441 |
| H | -6.850033 | -0.741895 | 1.099041 | O | 8.149112 | 3.595381 | 0.046758 |
| H | 5.813565 | -0.113838 | -1.427767 | H | 6.904880 | 5.071800 | -1.663242 |
| O | 5.367396 | -2.483145 | -1.886905 | H | 6.132718 | 3.603148 | -2.331659 |
| O | 3.621628 | 0.265205 | 2.350046 | H | 5.342093 | 4.480948 | -1.021573 |
| O | -2.382187 | -0.339202 | -2.320278 | - | - | - | - |

**Table S5** Atomic coordinates (Å) of **4a-2** obtained at the B3LYP-D3BJ/6-31G(d) level of theory in the gas phase.

| C | 4.641360 | 1.574628 | -1.125503 | O | -1.102176 | -2.530722 | -1.405229 |
| --- | --- | --- | --- | --- | --- | --- | --- |
| C | 5.563625 | 2.662214 | -1.641168 | H | -5.659740 | -3.768071 | -0.299206 |
| C | 6.915567 | 2.042943 | -1.995300 | H | 5.987205 | -2.268689 | 0.265511 |
| C | 7.629868 | 1.448969 | -0.764209 | H | 6.683277 | -1.335561 | 2.619454 |
| C | 6.708199 | 0.357142 | -0.124926 | H | -7.096658 | 2.653671 | -0.722528 |
| C | 7.069202 | 0.075874 | 1.332879 | O | -5.947042 | 2.162144 | 0.922269 |
| C | 2.507067 | 1.464673 | -0.030563 | H | -2.315973 | -1.145945 | 3.468660 |
| C | 2.734942 | -0.037330 | 0.005139 | H | -2.302548 | -2.565071 | 2.447833 |
| C | 4.243978 | -0.524910 | 0.119107 | H | -4.713686 | 3.105112 | -1.228841 |
| C | 5.192740 | 0.711141 | 0.012958 | H | -5.315243 | 1.892739 | -2.353936 |
| C | 4.473225 | -1.705679 | -0.851463 | H | -1.791080 | 0.974349 | 1.369886 |
| C | 3.337149 | -2.745711 | -0.728621 | H | -0.265661 | 0.682088 | 0.555575 |
| C | 1.907077 | -2.145135 | -0.718394 | H | -8.608883 | -0.610693 | 0.317419 |
| C | 1.999981 | -0.687962 | -1.197639 | H | -8.898563 | 1.133410 | 0.188540 |
| C | 2.023235 | -0.702658 | 1.199520 | H | -7.823169 | 0.500010 | 1.445282 |
| C | 1.338711 | -1.973764 | 0.713800 | H | 3.447930 | -3.450206 | -1.558540 |
| C | 1.356101 | -3.199590 | 1.655398 | H | 3.535898 | -3.321559 | 0.181593 |
| C | 8.918201 | 0.748680 | -1.233928 | H | -4.737213 | -0.372968 | -2.896769 |
| C | 8.036781 | 2.593991 | 0.188646 | H | -3.581379 | -1.584480 | -2.322432 |
| C | 5.172448 | 1.442291 | 1.380053 | H | 2.527526 | -0.577508 | -2.147267 |
| C | -4.015430 | 1.119942 | -0.813689 | H | 1.014654 | -0.237034 | -1.333417 |
| C | -5.085372 | 2.078271 | -1.301552 | H | 6.765093 | 1.249454 | -2.741846 |
| C | -6.342477 | 1.911382 | -0.455686 | H | 7.564451 | 2.791987 | -2.464355 |
| C | -6.949289 | 0.497357 | -0.564075 | H | 4.501288 | -1.327456 | -1.880210 |
| C | -5.877944 | -0.565745 | -0.155897 | H | 5.100744 | 3.131252 | -2.515832 |
| C | -6.188788 | -1.956090 | -0.709298 | H | 5.673522 | 3.446273 | -0.885720 |
| C | -1.854081 | 0.377624 | -1.520750 | H | -3.698676 | 0.095898 | 1.963873 |
| C | -1.903724 | -0.506662 | -0.286072 | H | -7.255226 | -2.151102 | -0.868662 |
| C | -3.334099 | -1.045203 | 0.149261 | H | 1.236090 | -2.738065 | -1.340244 |
| C | -4.435188 | -0.347369 | -0.702547 | H | 4.387372 | 0.921032 | -1.970175 |
| C | -3.494676 | -0.941634 | 1.671753 | H | -0.767357 | -3.818125 | 2.117062 |
| C | -2.256857 | -1.471525 | 2.424467 | H | -0.218273 | -4.163476 | 0.458632 |
| C | -0.905350 | -0.995744 | 1.817837 | H | -3.683976 | 1.466383 | 0.169230 |
| C | -1.185260 | 0.200203 | 0.893757 | H | 8.122323 | 0.241335 | 1.578649 |
| C | -1.058786 | -1.780870 | -0.452637 | H | 2.086787 | -3.980845 | 1.432690 |
| C | -0.280434 | -2.020409 | 0.835379 | H | 1.484464 | -2.873318 | 2.691696 |
| C | -0.113903 | -3.482505 | 1.308380 | H | -8.075470 | -0.583568 | -2.101269 |
| C | -8.139513 | 0.375009 | 0.405754 | H | -8.182965 | 1.171939 | -2.217047 |
| C | -7.502479 | 0.340950 | -1.998751 | H | -6.730467 | 0.328272 | -2.768513 |
| C | -4.510397 | -1.060658 | -2.075794 | H | -0.204556 | -0.740552 | 2.613432 |
| C | -6.663477 | 3.068065 | 1.636245 | H | 4.270496 | 1.227438 | 1.961235 |
| C | -6.100439 | 3.191573 | 3.030607 | H | 5.258665 | 2.527762 | 1.275236 |
| O | 6.312996 | 0.962226 | 2.115555 | H | 8.637079 | 3.324310 | -0.366244 |
| O | -5.574048 | -2.022575 | -1.976679 | H | 7.187030 | 3.124795 | 0.620530 |
| O | 6.750314 | -1.285564 | 1.651930 | H | 8.649302 | 2.234567 | 1.019941 |
| H | -4.364323 | -1.529535 | 1.973133 | H | 8.698379 | -0.027922 | -1.975078 |
| O | 5.715602 | -2.371173 | -0.667114 | H | 9.605386 | 1.470366 | -1.690650 |
| O | 3.425329 | 2.217769 | -0.674944 | H | 9.444587 | 0.273394 | -0.398030 |
| H | 4.377401 | -0.932687 | 1.125189 | O | 1.513920 | 1.994530 | 0.413042 |
| H | -3.377113 | -2.108075 | -0.096823 | O | -0.908435 | 0.417376 | -2.273860 |
| H | 6.823598 | -0.548720 | -0.718402 | O | -7.613644 | 3.678871 | 1.203209 |
| H | -5.869698 | -0.596263 | 0.932304 | H | -6.119858 | 2.217063 | 3.529412 |
| O | -5.665936 | -2.925909 | 0.184507 | H | -5.055312 | 3.514451 | 2.984999 |
| O | -2.897328 | 1.216146 | -1.725308 | H | -6.687861 | 3.912740 | 3.599033 |
| O | 2.103124 | -0.334270 | 2.352765 | - | - | - | - |

**Table S6** Key transitions, oscillator strengths, and rotatory strengths in the ECD spectrum of conformer **4A-1** at the PBE0/6-311+G(2d,p) level of theory in MeOH with IEFPCM solvent model.

| *Num^a^* | *Transition^b^* | *CI-coeff^b^* | *ΔE (eV)^d^* | *λ (nm)^e^* | *f^f^* | *R_vel_^g^* | *R_len_^h^* |
| --- | --- | --- | --- | --- | --- | --- | --- |
| 1 | 205->206 | 0.66201 | 3.7697 | 328.89 | 0.0015 | 21.8173 | 21.3122 |
| 2 | 203->206 | 0.54465 | 4.1186 | 301.03 | 0.0000 | 2.498 | 2.486 |
|  | 205->207 | 0.39629 |  |  |  |  |  |
| 3 | 204->206 | 0.67404 | 5.0186 | 247.05 | 0.0028 | 2.6304 | 2.4519 |
| 4 | 201->206 | 0.33984 | 5.2755 | 235.02 | 0.0142 | 18.3143 | 18.2925 |
|  | 202->206 | 0.40964 |  |  |  |  |  |
| 5 | 203->206 | -0.36886 | 5.3152 | 233.26 | 0.0114 | 5.1566 | 5.0794 |
|  | 205->207 | 0.50732 |  |  |  |  |  |
| 6 | 199->206 | 0.51081 | 5.4074 | 229.28 | 0.0172 | 13.4709 | 13.4445 |
| 7 | 201->206 | -0.37257 | 5.5752 | 222.38 | 0.0023 | -0.5804 | -0.5884 |
|  | 202->206 | 0.43624 |  |  |  |  |  |
| 8 | 201->206 | 0.22932 | 5.6040 | 221.24 | 0.0008 | -4.7431 | -4.7172 |
|  | 205->208 | 0.33756 |  |  |  |  |  |
| 9 | 200->206 | -0.38862 | 5.6622 | 218.97 | 0.0287 | 13.8349 | 13.7828 |
|  | 205->208 | 0.42668 |  |  |  |  |  |
| 10 | 193->206 | 0.2712 | 5.6668 | 218.79 | 0.0004 | -2.2387 | -2.1707 |
|  | 194->206 | 0.2791 |  |  |  |  |  |
| 11 | 199->208 | 0.23238 | 5.6918 | 217.83 | 0.0209 | -38.9914 | -38.797 |
|  | 203->207 | 0.41424 |  |  |  |  |  |
| 12 | 193->206 | -0.23238 | 5.6994 | 217.54 | 0.0401 | -35.9207 | -35.7804 |
|  | 201->206 | 0.24068 |  |  |  |  |  |
|  | 203->207 | 0.25017 |  |  |  |  |  |
| 13 | 200->206 | 0.37445 | 5.7480 | 215.70 | 0.0232 | -15.7351 | -15.7362 |
|  | 205->208 | 0.30583 |  |  |  |  |  |
| 14 | 203->208 | -0.23845 | 5.7822 | 214.43 | 0.0106 | -24.8624 | -24.6445 |
|  | 205->209 | 0.47576 |  |  |  |  |  |
| 15 | 198->206 | 0.53528 | 5.8316 | 212.61 | 0.0007 | -2.305 | -2.3226 |
| 16 | 196->206 | -0.23726 | 5.8549 | 211.76 | 0.0042 | -8.5324 | -8.5381 |
|  | 197->206 | 0.59384 |  |  |  |  |  |
| 17 | 193->206 | 0.48294 | 5.8958 | 210.29 | 0.0404 | 25.0209 | 25.0116 |
| 18 | 196->210 | 0.54764 | 5.9913 | 206.94 | 0.0009 | 3.4656 | 2.9898 |
|  | 198->210 | 0.28584 |  |  |  |  |  |
| 19 | 204->207 | 0.65987 | 6.0822 | 203.85 | 0.0068 | -2.8344 | -2.7876 |
| 20 | 192->206 | 0.28208 | 6.0903 | 203.58 | 0.0132 | -4.2833 | -4.2111 |
|  | 195->206 | 0.34687 |  |  |  |  |  |
|  | 196->206 | -0.28154 |  |  |  |  |  |
| 21 | 192->206 | 0.25247 | 6.2246 | 199.18 | 0.0060 | -1.4205 | -1.3212 |
|  | 195->206 | 0.23104 |  |  |  |  |  |
|  | 196->206 | 0.52006 |  |  |  |  |  |
| 22 | 191->206 | 0.57965 | 6.2529 | 198.28 | 0.0375 | -5.1099 | -4.8542 |
| 23 | 190->206 | 0.39141 | 6.3066 | 196.59 | 0.0023 | 4.0136 | 3.9807 |
|  | 192->206 | 0.40321 |  |  |  |  |  |
|  | 195->206 | -0.23822 |  |  |  |  |  |
| 24 | 189->206 | 0.41267 | 6.3415 | 195.51 | 0.0159 | -18.179 | -18.0835 |
|  | 190->206 | -0.228 |  |  |  |  |  |
|  | 194->206 | 0.24928 |  |  |  |  |  |
| 25 | 189->206 | 0.32569 | 6.3504 | 195.24 | 0.0310 | -13.1794 | -13.1669 |
|  | 190->206 | 0.40898 |  |  |  |  |  |
|  | 192->206 | -0.31423 |  |  |  |  |  |
| 26 | 201->207 | 0.23982 | 6.3987 | 193.76 | 0.0007 | -1.102 | -1.0526 |
|  | 202->207 | 0.41192 |  |  |  |  |  |
|  | 204->208 | 0.33422 |  |  |  |  |  |
| 27 | 202->207 | -0.32744 | 6.4070 | 193.51 | 0.0001 | -1.1998 | -1.2036 |
|  | 204->208 | 0.3933 |  |  |  |  |  |
|  | 204->209 | -0.23568 |  |  |  |  |  |
| 28 | 185->206 | 0.22367 | 6.4662 | 191.74 | 0.0171 | -14.6744 | -14.7297 |
|  | 188->206 | 0.53949 |  |  |  |  |  |
| 29 | 203->208 | 0.53567 | 6.4878 | 191.10 | 0.0044 | 4.5535 | 4.49 |
|  | 205->209 | 0.23017 |  |  |  |  |  |
| 30 | 188->206 | 0.35226 | 6.5155 | 190.29 | 0.0061 | 6.2959 | 6.2688 |
|  | 199->207 | 0.31653 |  |  |  |  |  |
| 31 | 205->211 | 0.45305 | 6.5538 | 189.18 | 0.0015 | -3.3256 | -3.1787 |
| 32 | 185->206 | -0.26502 | 6.5610 | 188.97 | 0.0066 | -2.9151 | -2.8984 |
|  | 199->207 | -0.27657 |  |  |  |  |  |
|  | 205->211 | 0.31907 |  |  |  |  |  |
| 33 | 203->209 | 0.50085 | 6.5758 | 188.55 | 0.0017 | -0.2776 | -0.3008 |
| 34 | 187->206 | 0.41125 | 6.6077 | 187.64 | 0.0029 | 1.4857 | 1.3918 |
|  | 201->207 | -0.2839 |  |  |  |  |  |
|  | 202->207 | 0.22829 |  |  |  |  |  |
| 35 | 187->206 | 0.34587 | 6.6141 | 187.46 | 0.0074 | -1.7744 | -1.677 |
|  | 201->207 | 0.31946 |  |  |  |  |  |
| 36 | 205->210 | 0.39375 | 6.6606 | 186.14 | 0.0003 | 1.062 | 0.9955 |
|  | 205->213 | 0.47263 |  |  |  |  |  |

*^a^*Number of the excited states; *^b^*Only transitions with contribution over 10.0% were listed; *^c^*Configuration-interaction coefficient; *^d^*Excitation energy; *^e^*Wavelength; *^f^*Oscillator strength; *^g^*Rotatory strength in velocity form (10^-40^ cgs); *^h^*Rotatory strength in length form (10^-40^ cgs).

**Table S7** Key transitions, oscillator strengths, and rotatory strengths in the ECD spectrum of conformer **4A-2** at the PBE0/6-311+G(2d,p) level of theory in MeOH with IEFPCM solvent model.

| *Num^a^* | *Transition^b^* | *CI-coeff^b^* | *ΔE (eV)^d^* | *λ (nm)^e^* | *f^f^* | *R_vel_^g^* | *R_len_^h^* |
| --- | --- | --- | --- | --- | --- | --- | --- |
| 1 | 203->207 | 0.25374 | 3.8286 | 323.84 | 0.0017 | 19.5013 | 19.563 |
|  | 205->206 | 0.64069 |  |  |  |  |  |
| 2 | 203->206 | 0.53858 | 4.0281 | 307.80 | 0.0002 | 4.3356 | 4.2682 |
|  | 205->207 | 0.41373 |  |  |  |  |  |
| 3 | 204->206 | 0.65957 | 5.0352 | 246.23 | 0.0084 | -4.3707 | -4.3786 |
| 4 | 203->206 | -0.42292 | 5.1261 | 241.87 | 0.0023 | -2.6302 | -2.6422 |
|  | 205->207 | 0.53983 |  |  |  |  |  |
| 5 | 201->206 | 0.32367 | 5.2681 | 235.35 | 0.0059 | 6.0606 | 5.9447 |
|  | 202->206 | 0.49221 |  |  |  |  |  |
| 6 | 203->207 | 0.58153 | 5.3583 | 231.39 | 0.0395 | -20.4097 | -20.3086 |
|  | 205->206 | -0.24784 |  |  |  |  |  |
| 7 | 199->206 | 0.50796 | 5.4169 | 228.88 | 0.0087 | 16.8258 | 16.8233 |
|  | 201->206 | 0.23873 |  |  |  |  |  |
| 8 | 201->206 | 0.42409 | 5.6121 | 220.92 | 0.0012 | -1.7258 | -1.7482 |
|  | 202->206 | -0.41324 |  |  |  |  |  |
| 9 | 200->206 | 0.43676 | 5.6609 | 219.02 | 0.0565 | -84.9045 | -84.5996 |
| 10 | 199->208 | 0.31441 | 5.6825 | 218.18 | 0.0021 | 0.0324 | 0.0084 |
|  | 201->209 | -0.25049 |  |  |  |  |  |
|  | 202->209 | -0.22513 |  |  |  |  |  |
| 11 | 199->206 | -0.29862 | 5.7264 | 216.51 | 0.0012 | 14.7708 | 14.5181 |
|  | 200->206 | 0.41609 |  |  |  |  |  |
| 12 | 203->209 | -0.25769 | 5.7667 | 215.00 | 0.0183 | -27.4869 | -27.2238 |
|  | 205->208 | 0.57138 |  |  |  |  |  |
| 13 | 203->208 | -0.28922 | 5.7764 | 214.64 | 0.0163 | 11.2846 | 11.2137 |
|  | 205->209 | 0.54186 |  |  |  |  |  |
| 14 | 204->207 | 0.6283 | 5.8214 | 212.98 | 0.0118 | 10.9658 | 10.9933 |
| 15 | 196->206 | 0.25978 | 5.8342 | 212.51 | 0.0010 | 1.3549 | 1.3131 |
|  | 197->206 | 0.3812 |  |  |  |  |  |
|  | 198->206 | -0.37656 |  |  |  |  |  |
| 16 | 197->206 | 0.49682 | 5.8625 | 211.49 | 0.0008 | -0.8028 | -0.8111 |
|  | 198->206 | 0.38976 |  |  |  |  |  |
| 17 | 194->206 | 0.35395 | 5.9143 | 209.64 | 0.0726 | 13.5818 | 13.658 |
|  | 195->206 | 0.42734 |  |  |  |  |  |
| 18 | 196->210 | 0.55378 | 5.9921 | 206.91 | 0.0009 | 3.4893 | 3.2542 |
|  | 198->210 | 0.32956 |  |  |  |  |  |
| 19 | 188->206 | -0.3027 | 6.0050 | 206.47 | 0.0031 | 0.022 | 0.0335 |
|  | 189->206 | 0.37531 |  |  |  |  |  |
|  | 191->206 | 0.29854 |  |  |  |  |  |
| 20 | 201->207 | 0.29109 | 6.0652 | 204.42 | 0.0096 | -12.8126 | -12.6386 |
|  | 202->207 | 0.45454 |  |  |  |  |  |
| 21 | 194->206 | 0.27161 | 6.1468 | 201.70 | 0.0068 | -4.7029 | -4.7297 |
|  | 202->207 | -0.25382 |  |  |  |  |  |
| 22 | 192->206 | 0.23346 | 6.2384 | 198.75 | 0.0139 | -3.0677 | -2.8585 |
|  | 196->206 | 0.42449 |  |  |  |  |  |
|  | 199->207 | 0.25503 |  |  |  |  |  |
| 23 | 193->206 | 0.39733 | 6.2499 | 198.38 | 0.0406 | -20.7604 | -20.6069 |
|  | 196->206 | 0.34668 |  |  |  |  |  |
| 24 | 190->206 | -0.25948 | 6.2677 | 197.82 | 0.0320 | -12.1752 | -12.1956 |
|  | 199->207 | 0.38984 |  |  |  |  |  |
|  | 201->207 | 0.27234 |  |  |  |  |  |
| 25 | 190->206 | -0.2969 | 6.3091 | 196.52 | 0.0014 | 0.2903 | 0.2385 |
|  | 191->206 | 0.22532 |  |  |  |  |  |
|  | 192->206 | 0.36451 |  |  |  |  |  |
| 26 | 203->208 | 0.42652 | 6.3300 | 195.87 | 0.0031 | 3.1458 | 3.1133 |
|  | 204->208 | 0.31446 |  |  |  |  |  |
| 27 | 191->206 | 0.23876 | 6.3442 | 195.43 | 0.0043 | -1.3815 | -1.3987 |
|  | 193->206 | 0.29213 |  |  |  |  |  |
|  | 194->206 | -0.27727 |  |  |  |  |  |
| 28 | 192->206 | 0.25399 | 6.3721 | 194.57 | 0.0087 | -0.1192 | -0.1497 |
|  | 193->206 | 0.31149 |  |  |  |  |  |
|  | 194->206 | -0.22734 |  |  |  |  |  |
|  | 201->207 | -0.25393 |  |  |  |  |  |
| 29 | 191->206 | -0.29518 | 6.3875 | 194.10 | 0.0094 | 0.2787 | 0.301 |
|  | 199->207 | -0.27558 |  |  |  |  |  |
|  | 201->207 | 0.3405 |  |  |  |  |  |
|  | 202->207 | -0.25603 |  |  |  |  |  |
| 30 | 203->208 | 0.32427 | 6.4021 | 193.66 | 0.0029 | 4.7029 | 4.6029 |
|  | 203->209 | 0.4713 |  |  |  |  |  |
|  | 205->209 | 0.24508 |  |  |  |  |  |
| 31 | 203->208 | -0.25824 | 6.4348 | 192.68 | 0.0060 | -1.779 | -1.7529 |
|  | 203->209 | 0.34006 |  |  |  |  |  |
|  | 204->208 | 0.40183 |  |  |  |  |  |
| 32 | 188->206 | 0.39226 | 6.4658 | 191.75 | 0.0070 | -13.8735 | -13.8023 |
|  | 189->206 | 0.29976 |  |  |  |  |  |
| 33 | 188->206 | 0.23562 | 6.5016 | 190.70 | 0.0079 | -3.02 | -3.0058 |
|  | 199->207 | -0.24025 |  |  |  |  |  |
|  | 200->207 | 0.48966 |  |  |  |  |  |
| 34 | 183->206 | 0.22845 | 6.5552 | 189.14 | 0.0053 | 6.1504 | 6.0809 |
|  | 185->206 | 0.2914 |  |  |  |  |  |
|  | 189->206 | -0.2558 |  |  |  |  |  |
|  | 198->207 | -0.28191 |  |  |  |  |  |
| 35 | 185->206 | 0.3226 | 6.5914 | 188.10 | 0.0033 | 2.3377 | 2.3282 |
|  | 198->207 | 0.36601 |  |  |  |  |  |
| 36 | 187->206 | 0.41488 | 6.6259 | 187.12 | 0.0015 | -1.0842 | -1.0603 |
|  | 197->207 | 0.23547 |  |  |  |  |  |

*^a^*Number of the excited states; *^b^*Only transitions with contribution over 10.0% were listed; *^c^*Configuration-interaction coefficient; *^d^*Excitation energy; *^e^*Wavelength; *^f^*Oscillator strength; *^g^*Rotatory strength in velocity form (10^-40^ cgs); *^h^*Rotatory strength in length form (10^-40^ cgs).

**Table S8** Key transitions, oscillator strengths, and rotatory strengths in the ECD spectrum of conformer **4A-1** at the CAM-B3LYP/6-311+G(2d,p) level of theory in MeOH with IEFPCM solvent model.

| *Num^a^* | *Transition^b^* | *CI-coeff^b^* | *ΔE (eV)^d^* | *λ (nm)^e^* | *f^f^* | *R_vel_^g^* | *R_len_^h^* |
| --- | --- | --- | --- | --- | --- | --- | --- |
| 1 | 203->207 | 0.28624 | 3.9809 | 311.45 | 0.0004 | 15.1555 | 15.2253 |
|  | 205->206 | 0.60884 |  |  |  |  |  |
| 2 | 203->206 | 0.50323 | 4.2145 | 294.19 | 0.0000 | 3.4006 | 3.5737 |
|  | 205->207 | 0.41466 |  |  |  |  |  |
| 3 | 198->212 | 0.22273 | 5.6690 | 218.70 | 0.0002 | 4.5483 | 4.5299 |
|  | 201->212 | -0.19302 |  |  |  |  |  |
|  | 202->212 | 0.22376 |  |  |  |  |  |
| 4 | 199->206 | -0.16372 | 5.6825 | 218.19 | 0.0005 | 0.6003 | 0.6003 |
|  | 199->209 | 0.22519 |  |  |  |  |  |
|  | 199->210 | 0.20663 |  |  |  |  |  |
|  | 199->212 | 0.20294 |  |  |  |  |  |
| 5 | 196->216 | -0.23406 | 6.0156 | 206.10 | 0.0008 | 3.5057 | 3.3207 |
|  | 196->217 | -0.38383 |  |  |  |  |  |
|  | 196->218 | -0.24505 |  |  |  |  |  |
| 6 | 193->206 | -0.22327 | 6.0919 | 203.52 | 0.1014 | 2.8514 | 2.9045 |
|  | 199->206 | -0.18772 |  |  |  |  |  |
|  | 201->206 | 0.22606 |  |  |  |  |  |
|  | 202->206 | -0.21079 |  |  |  |  |  |
|  | 204->206 | 0.41658 |  |  |  |  |  |
| 7 | 199->206 | 0.34615 | 6.1905 | 200.28 | 0.0365 | -18.4475 | -18.5921 |
|  | 204->206 | 0.38417 |  |  |  |  |  |
|  | 204->207 | 0.16869 |  |  |  |  |  |
| 8 | 194->206 | 0.4257 | 6.2320 | 198.95 | 0.0081 | 0.6688 | 0.6653 |
|  | 195->206 | 0.41647 |  |  |  |  |  |
|  | 203->206 | 0.19212 |  |  |  |  |  |
|  | 205->207 | -0.15848 |  |  |  |  |  |
| 9 | 198->206 | 0.23105 | 6.3696 | 194.65 | 0.0137 | 17.1738 | 17.0179 |
|  | 200->206 | 0.19979 |  |  |  |  |  |
|  | 202->206 | 0.26537 |  |  |  |  |  |
|  | 203->206 | 0.1956 |  |  |  |  |  |
|  | 205->212 | -0.16943 |  |  |  |  |  |
| 10 | 203->212 | 0.30098 | 6.4283 | 192.87 | 0.0377 | -2.8916 | -2.8364 |
|  | 205->208 | -0.17787 |  |  |  |  |  |
|  | 205->209 | 0.40453 |  |  |  |  |  |
|  | 205->210 | 0.18481 |  |  |  |  |  |
|  | 205->211 | -0.18194 |  |  |  |  |  |
| 11 | 194->206 | 0.19022 | 6.4810 | 191.30 | 0.0028 | -2.4415 | -2.3995 |
|  | 203->206 | -0.29869 |  |  |  |  |  |
|  | 203->207 | -0.19424 |  |  |  |  |  |
|  | 205->207 | 0.33251 |  |  |  |  |  |
|  | 205->212 | 0.17584 |  |  |  |  |  |
| 12 | 203->209 | 0.27568 | 6.4999 | 190.75 | 0.0147 | -26.2352 | -26.0375 |
|  | 205->207 | -0.2635 |  |  |  |  |  |
|  | 205->210 | 0.16433 |  |  |  |  |  |
|  | 205->212 | 0.41257 |  |  |  |  |  |
| 13 | 192->206 | -0.16897 | 6.6169 | 187.37 | 0.0106 | -10.1336 | -10.1475 |
|  | 202->206 | 0.17562 |  |  |  |  |  |
|  | 203->207 | 0.38226 |  |  |  |  |  |
|  | 205->206 | -0.20787 |  |  |  |  |  |
|  | 205->207 | 0.20688 |  |  |  |  |  |
| 14 | 192->206 | 0.25114 | 6.7327 | 184.15 | 0.0089 | -11.8562 | -11.8814 |
|  | 193->206 | -0.23901 |  |  |  |  |  |
|  | 194->206 | 0.18129 |  |  |  |  |  |
|  | 199->206 | 0.24873 |  |  |  |  |  |
|  | 203->207 | 0.23765 |  |  |  |  |  |
|  | 204->206 | -0.18668 |  |  |  |  |  |
| 15 | 194->206 | 0.25855 | 6.8225 | 181.73 | 0.0121 | -7.6891 | -7.5973 |
|  | 195->206 | -0.16006 |  |  |  |  |  |
|  | 201->206 | 0.29619 |  |  |  |  |  |
|  | 202->206 | 0.20862 |  |  |  |  |  |
| 16 | 191->206 | 0.19911 | 6.9894 | 177.39 | 0.0127 | -14.8236 | -14.8284 |
|  | 197->206 | 0.38973 |  |  |  |  |  |
|  | 197->207 | 0.16718 |  |  |  |  |  |
|  | 198->206 | -0.18666 |  |  |  |  |  |
|  | 200->206 | -0.18876 |  |  |  |  |  |
| 17 | 189->206 | -0.22941 | 7.0370 | 176.19 | 0.0651 | -56.3472 | -56.3971 |
|  | 198->206 | 0.34633 |  |  |  |  |  |
|  | 200->206 | -0.21107 |  |  |  |  |  |
|  | 202->206 | -0.19524 |  |  |  |  |  |
| 18 | 183->206 | 0.25495 | 7.0944 | 174.76 | 0.0433 | -10.6733 | -10.6735 |
|  | 185->206 | -0.17705 |  |  |  |  |  |
|  | 194->207 | -0.17421 |  |  |  |  |  |
|  | 195->207 | -0.21334 |  |  |  |  |  |
|  | 201->206 | 0.19441 |  |  |  |  |  |
|  | 202->206 | 0.25414 |  |  |  |  |  |
| 19 | 197->206 | 0.28155 | 7.1224 | 174.08 | 0.0089 | -1.1682 | -1.2091 |
|  | 200->206 | 0.25774 |  |  |  |  |  |
|  | 200->207 | 0.19221 |  |  |  |  |  |
|  | 201->206 | 0.19893 |  |  |  |  |  |
| 20 | 205->208 | -0.27811 | 7.1331 | 173.82 | 0.0111 | 22.6385 | 22.2223 |
|  | 205->210 | 0.29381 |  |  |  |  |  |
|  | 205->211 | 0.2123 |  |  |  |  |  |
|  | 205->215 | 0.19833 |  |  |  |  |  |
| 21 | 191->206 | -0.1815 | 7.2269 | 171.56 | 0.0051 | 18.0069 | 17.928 |
|  | 194->207 | 0.30033 |  |  |  |  |  |
|  | 200->206 | -0.22089 |  |  |  |  |  |
| 22 | 191->206 | 0.17517 | 7.2416 | 171.21 | 0.0251 | 7.3068 | 7.3081 |
|  | 192->206 | 0.34221 |  |  |  |  |  |
|  | 205->213 | -0.15895 |  |  |  |  |  |
| 23 | 192->206 | 0.1628 | 7.2538 | 170.92 | 0.0476 | -66.4781 | -66.5075 |
|  | 200->206 | -0.17529 |  |  |  |  |  |
|  | 205->213 | 0.31422 |  |  |  |  |  |
|  | 205->218 | 0.18272 |  |  |  |  |  |
| 24 | 189->207 | 0.18719 | 7.2753 | 170.42 | 0.0121 | -13.2232 | -13.2243 |
|  | 193->207 | 0.23516 |  |  |  |  |  |
|  | 194->206 | -0.15997 |  |  |  |  |  |
|  | 199->207 | 0.16148 |  |  |  |  |  |
|  | 201->206 | 0.18624 |  |  |  |  |  |
|  | 201->207 | -0.17922 |  |  |  |  |  |
|  | 204->207 | -0.21987 |  |  |  |  |  |
| 25 | 204->209 | 0.28507 | 7.3015 | 169.81 | 0.0186 | 16.7801 | 16.5836 |
|  | 204->210 | -0.24421 |  |  |  |  |  |
|  | 204->211 | 0.15844 |  |  |  |  |  |
|  | 204->216 | -0.25215 |  |  |  |  |  |
| 26 | 204->209 | 0.26868 | 7.4036 | 167.46 | 0.0508 | 10.2122 | 9.8747 |
|  | 204->210 | 0.22931 |  |  |  |  |  |
|  | 204->212 | 0.20114 |  |  |  |  |  |
| 27 | 180->206 | 0.16138 | 7.4113 | 167.29 | 0.0040 | -12.303 | -12.2611 |
|  | 187->206 | 0.28672 |  |  |  |  |  |
|  | 193->206 | 0.16149 |  |  |  |  |  |
| 28 | 185->206 | 0.1823 | 7.4325 | 166.81 | 0.0172 | 3.1031 | 4.3055 |
|  | 201->208 | -0.16156 |  |  |  |  |  |
| 29 | 201->206 | 0.1795 | 7.4752 | 165.86 | 0.0119 | -57.0486 | -56.7308 |
| 30 | 181->206 | 0.19145 | 7.4799 | 165.76 | 0.0256 | 2.8176 | 2.8579 |
|  | 183->206 | 0.26391 |  |  |  |  |  |
|  | 194->207 | 0.24883 |  |  |  |  |  |
|  | 195->207 | 0.29711 |  |  |  |  |  |
| 31 | 205->208 | 0.33782 | 7.5082 | 165.13 | 0.0167 | -33.2907 | -33.3183 |
|  | 205->210 | 0.2302 |  |  |  |  |  |
|  | 205->211 | 0.23804 |  |  |  |  |  |
|  | 205->219 | 0.15813 |  |  |  |  |  |
| 32 | 200->209 | 0.16484 | 7.5309 | 164.63 | 0.0094 | -28.0285 | -27.8944 |
|  | 204->213 | -0.17849 |  |  |  |  |  |
|  | 205->208 | 0.17315 |  |  |  |  |  |
|  | 205->211 | 0.21325 |  |  |  |  |  |
| 33 | 204->210 | 0.17058 | 7.5398 | 164.44 | 0.2092 | 65.4984 | 65.0476 |
| 34 | 199->207 | 0.16087 | 7.5558 | 164.09 | 0.0204 | 60.3733 | 59.8827 |
|  | 204->207 | 0.3576 |  |  |  |  |  |
| 35 | 205->210 | 0.15863 | 7.5752 | 163.67 | 0.0093 | -6.1713 | -6.348 |
|  | 205->211 | -0.26725 |  |  |  |  |  |
|  | 205->214 | 0.34789 |  |  |  |  |  |
|  | 205->215 | 0.17034 |  |  |  |  |  |
|  | 205->220 | 0.17386 |  |  |  |  |  |
| 36 | 202->208 | 0.15836 | 7.6076 | 162.97 | 0.0171 | -10.6281 | -11.1295 |

*^a^*Number of the excited states; *^b^*Only transitions with contribution over 5.0% were listed; *^c^*Configuration-interaction coefficient; *^d^*Excitation energy; *^e^*Wavelength; *^f^*Oscillator strength; *^g^*Rotatory strength in velocity form (10^-40^ cgs); *^h^*Rotatory strength in length form (10^-40^ cgs).

**Table S9** Key transitions, oscillator strengths, and rotatory strengths in the ECD spectrum of conformer **4A-2** at the CAM-B3LYP/6-311+G(2d,p) level of theory in MeOH with IEFPCM solvent model.

| *Num^a^* | *Transition^b^* | *CI-coeff^b^* | *ΔE (eV)^d^* | *λ (nm)^e^* | *f^f^* | *R_vel_^g^* | *R_len_^h^* |
| --- | --- | --- | --- | --- | --- | --- | --- |
| 1 | 203->207 | 0.31705 | 4.0038 | 309.66 | 0.0006 | 13.5504 | 13.7871 |
|  | 205->206 | 0.58253 |  |  |  |  |  |
| 2 | 203->206 | 0.48366 | 4.1315 | 300.10 | 0.0002 | 4.1257 | 4.1262 |
|  | 204->206 | 0.14732 |  |  |  |  |  |
|  | 205->207 | 0.43341 |  |  |  |  |  |
| 3 | 198->210 | 0.15749 | 5.6715 | 218.61 | 0.0007 | -5.3426 | -5.3162 |
|  | 198->213 | 0.17156 |  |  |  |  |  |
|  | 201->210 | -0.15024 |  |  |  |  |  |
|  | 201->213 | -0.15608 |  |  |  |  |  |
|  | 202->206 | 0.15966 |  |  |  |  |  |
|  | 202->210 | 0.21167 |  |  |  |  |  |
|  | 202->211 | -0.14596 |  |  |  |  |  |
|  | 202->213 | 0.22875 |  |  |  |  |  |
| 4 | 199->206 | -0.1515 | 5.6817 | 218.22 | 0.0018 | -10.5782 | -10.5551 |
|  | 199->209 | -0.24595 |  |  |  |  |  |
|  | 199->210 | 0.25942 |  |  |  |  |  |
|  | 199->213 | -0.17833 |  |  |  |  |  |
| 5 | 196->215 | 0.17255 | 6.0164 | 206.08 | 0.0008 | 3.2452 | 2.992 |
|  | 196->216 | 0.14449 |  |  |  |  |  |
|  | 196->217 | -0.43051 |  |  |  |  |  |
|  | 196->219 | -0.16842 |  |  |  |  |  |
|  | 196->220 | 0.14539 |  |  |  |  |  |
|  | 196->222 | -0.1638 |  |  |  |  |  |
|  | 198->217 | -0.16898 |  |  |  |  |  |
| 6 | 203->206 | -0.40395 | 6.1148 | 202.76 | 0.0076 | -0.5611 | -0.5705 |
|  | 204->206 | -0.14503 |  |  |  |  |  |
|  | 205->207 | 0.45013 |  |  |  |  |  |
| 7 | 203->206 | -0.17401 | 6.1319 | 202.20 | 0.0349 | 4.359 | 4.3473 |
|  | 204->206 | 0.49184 |  |  |  |  |  |
|  | 204->207 | 0.22878 |  |  |  |  |  |
| 8 | 199->206 | 0.30078 | 6.1916 | 200.24 | 0.1221 | -65.5928 | -65.9472 |
|  | 200->206 | 0.16445 |  |  |  |  |  |
|  | 202->206 | -0.27014 |  |  |  |  |  |
|  | 203->207 | 0.29585 |  |  |  |  |  |
|  | 205->206 | -0.1875 |  |  |  |  |  |
| 9 | 198->206 | 0.14673 | 6.3086 | 196.53 | 0.0124 | 0.572 | 0.6292 |
|  | 202->206 | 0.29713 |  |  |  |  |  |
|  | 202->207 | -0.15125 |  |  |  |  |  |
|  | 203->207 | 0.37866 |  |  |  |  |  |
|  | 205->206 | -0.23274 |  |  |  |  |  |
| 10 | 190->206 | -0.16154 | 6.3671 | 194.73 | 0.0438 | 16.3503 | 16.3982 |
|  | 195->206 | -0.17238 |  |  |  |  |  |
|  | 199->206 | 0.2832 |  |  |  |  |  |
|  | 200->206 | 0.2402 |  |  |  |  |  |
|  | 201->206 | -0.23404 |  |  |  |  |  |
|  | 202->206 | 0.14842 |  |  |  |  |  |
|  | 203->207 | -0.1474 |  |  |  |  |  |
|  | 204->206 | 0.20266 |  |  |  |  |  |
| 11 | 203->210 | -0.20998 | 6.4989 | 190.78 | 0.0136 | -7.8795 | -7.8507 |
|  | 203->211 | 0.1425 |  |  |  |  |  |
|  | 203->213 | -0.22395 |  |  |  |  |  |
|  | 205->210 | 0.30543 |  |  |  |  |  |
|  | 205->211 | -0.22055 |  |  |  |  |  |
|  | 205->213 | 0.32879 |  |  |  |  |  |
| 12 | 203->209 | -0.2289 | 6.5167 | 190.25 | 0.0211 | 0.1445 | 0.1913 |
|  | 203->210 | 0.22731 |  |  |  |  |  |
|  | 203->213 | -0.16512 |  |  |  |  |  |
|  | 205->209 | -0.31452 |  |  |  |  |  |
|  | 205->210 | 0.32435 |  |  |  |  |  |
|  | 205->213 | -0.20376 |  |  |  |  |  |
| 13 | 188->206 | 0.15363 | 6.6550 | 186.30 | 0.0077 | -0.1409 | -0.1214 |
|  | 189->206 | 0.28073 |  |  |  |  |  |
|  | 191->206 | -0.14939 |  |  |  |  |  |
|  | 195->206 | 0.29656 |  |  |  |  |  |
|  | 201->206 | -0.25847 |  |  |  |  |  |
|  | 205->207 | 0.18254 |  |  |  |  |  |
| 14 | 189->206 | 0.24654 | 6.6922 | 185.27 | 0.0150 | -5.0525 | -5.0452 |
|  | 191->206 | -0.14696 |  |  |  |  |  |
|  | 192->206 | 0.23604 |  |  |  |  |  |
|  | 193->206 | 0.20588 |  |  |  |  |  |
|  | 194->206 | -0.15302 |  |  |  |  |  |
|  | 195->206 | -0.2115 |  |  |  |  |  |
|  | 199->206 | -0.1725 |  |  |  |  |  |
|  | 199->207 | -0.16023 |  |  |  |  |  |
|  | 204->206 | -0.16431 |  |  |  |  |  |
| 15 | 185->206 | -0.16718 | 6.8211 | 181.77 | 0.0163 | -17.3617 | -17.1246 |
|  | 189->206 | -0.18008 |  |  |  |  |  |
|  | 194->206 | -0.14451 |  |  |  |  |  |
|  | 195->206 | 0.1561 |  |  |  |  |  |
|  | 201->206 | -0.23086 |  |  |  |  |  |
|  | 201->207 | 0.24678 |  |  |  |  |  |
|  | 202->206 | -0.15427 |  |  |  |  |  |
| 16 | 189->206 | -0.1985 | 6.9593 | 178.16 | 0.0026 | 3.7139 | 3.779 |
|  | 191->206 | -0.17413 |  |  |  |  |  |
|  | 195->207 | 0.15906 |  |  |  |  |  |
|  | 197->206 | 0.3789 |  |  |  |  |  |
|  | 200->206 | -0.16534 |  |  |  |  |  |
|  | 200->207 | -0.14771 |  |  |  |  |  |
|  | 204->207 | -0.17674 |  |  |  |  |  |
| 17 | 189->207 | 0.15588 | 7.0233 | 176.53 | 0.0923 | -54.7441 | -54.7041 |
|  | 190->206 | 0.26417 |  |  |  |  |  |
|  | 194->206 | -0.16316 |  |  |  |  |  |
|  | 198->206 | -0.24569 |  |  |  |  |  |
|  | 198->207 | 0.14375 |  |  |  |  |  |
|  | 204->207 | -0.1936 |  |  |  |  |  |
| 18 | 197->206 | -0.16138 | 7.0614 | 175.58 | 0.0049 | 3.7127 | 3.8322 |
|  | 198->206 | 0.27974 |  |  |  |  |  |
|  | 201->206 | -0.15577 |  |  |  |  |  |
|  | 202->206 | -0.24255 |  |  |  |  |  |
|  | 202->207 | 0.22818 |  |  |  |  |  |
| 19 | 183->206 | -0.22186 | 7.1194 | 174.15 | 0.0352 | -8.3149 | -8.2408 |
|  | 185->206 | 0.21242 |  |  |  |  |  |
|  | 189->207 | 0.15226 |  |  |  |  |  |
|  | 192->207 | 0.1509 |  |  |  |  |  |
|  | 195->207 | -0.14659 |  |  |  |  |  |
|  | 197->206 | 0.24524 |  |  |  |  |  |
|  | 200->207 | 0.14269 |  |  |  |  |  |
| 20 | 193->206 | 0.18467 | 7.1430 | 173.57 | 0.0066 | 2.2334 | 2.2508 |
|  | 194->206 | 0.1449 |  |  |  |  |  |
|  | 195->206 | 0.20554 |  |  |  |  |  |
|  | 195->207 | 0.16027 |  |  |  |  |  |
|  | 200->206 | 0.3014 |  |  |  |  |  |
|  | 204->207 | -0.21547 |  |  |  |  |  |
| 21 | 205->208 | 0.26158 | 7.1956 | 172.30 | 0.0176 | 16.6381 | 16.0901 |
|  | 205->210 | -0.18203 |  |  |  |  |  |
|  | 205->211 | -0.17247 |  |  |  |  |  |
|  | 205->212 | 0.22014 |  |  |  |  |  |
|  | 205->215 | -0.14182 |  |  |  |  |  |
| 22 | 184->206 | 0.14443 | 7.2077 | 172.02 | 0.0016 | 7.8892 | 7.9505 |
|  | 193->206 | -0.15078 |  |  |  |  |  |
|  | 198->207 | 0.14659 |  |  |  |  |  |
|  | 199->206 | -0.14441 |  |  |  |  |  |
|  | 200->206 | 0.31256 |  |  |  |  |  |
|  | 200->207 | 0.1514 |  |  |  |  |  |
|  | 202->207 | 0.19225 |  |  |  |  |  |
|  | 205->211 | 0.14147 |  |  |  |  |  |
| 23 | 200->206 | -0.16072 | 7.2397 | 171.26 | 0.0435 | -37.4792 | -38.0794 |
|  | 203->211 | 0.15399 |  |  |  |  |  |
|  | 205->208 | 0.1984 |  |  |  |  |  |
|  | 205->211 | 0.31036 |  |  |  |  |  |
| 24 | 183->206 | 0.16619 | 7.2419 | 171.20 | 0.0182 | -25.0096 | -25.2439 |
|  | 190->207 | -0.1533 |  |  |  |  |  |
|  | 199->206 | -0.17619 |  |  |  |  |  |
|  | 199->207 | 0.27706 |  |  |  |  |  |
|  | 201->206 | -0.16429 |  |  |  |  |  |
|  | 203->207 | 0.14245 |  |  |  |  |  |
| 25 | 195->206 | 0.16424 | 7.2874 | 170.14 | 0.0089 | 11.0281 | 10.8821 |
|  | 201->206 | 0.17496 |  |  |  |  |  |
|  | 204->207 | -0.17397 |  |  |  |  |  |
|  | 204->209 | -0.15825 |  |  |  |  |  |
|  | 204->210 | -0.15395 |  |  |  |  |  |
|  | 204->216 | 0.18261 |  |  |  |  |  |
| 26 | 176->206 | 0.20166 | 7.3054 | 169.72 | 0.0102 | 20.0629 | 19.7928 |
|  | 189->207 | 0.17327 |  |  |  |  |  |
|  | 204->209 | 0.21287 |  |  |  |  |  |
|  | 204->216 | -0.20161 |  |  |  |  |  |
| 27 | 189->207 | 0.15 | 7.3451 | 168.80 | 0.0065 | -21.3013 | -20.9709 |
|  | 193->206 | 0.24576 |  |  |  |  |  |
|  | 195->207 | 0.15154 |  |  |  |  |  |
|  | 201->206 | -0.14418 |  |  |  |  |  |
|  | 202->207 | 0.17997 |  |  |  |  |  |
|  | 204->207 | 0.21306 |  |  |  |  |  |
| 28 | 204->209 | 0.32181 | 7.4079 | 167.37 | 0.0332 | -11.3791 | -11.6249 |
|  | 204->210 | -0.31823 |  |  |  |  |  |
|  | 204->213 | 0.22069 |  |  |  |  |  |
| 29 | 185->206 | 0.1455 | 7.4254 | 166.97 | 0.0548 | -9.6687 | -9.6303 |
|  | 187->206 | 0.2136 |  |  |  |  |  |
|  | 204->207 | -0.15372 |  |  |  |  |  |
| 30 | 201->208 | -0.1531 | 7.4447 | 166.54 | 0.0266 | -33.7448 | -33.5875 |
|  | 202->207 | 0.19467 |  |  |  |  |  |
| 31 | 195->207 | -0.14508 | 7.4493 | 166.44 | 0.0077 | 24.5555 | 25.2312 |
|  | 201->208 | 0.18023 |  |  |  |  |  |
|  | 202->206 | 0.15004 |  |  |  |  |  |
|  | 204->207 | -0.16546 |  |  |  |  |  |
| 32 | 180->206 | -0.18475 | 7.5095 | 165.10 | 0.2350 | 101.7137 | 101.1236 |
| 33 | 187->206 | 0.15259 | 7.5281 | 164.70 | 0.0272 | -53.5006 | -53.5662 |
| 34 | 200->209 | 0.22619 | 7.5327 | 164.60 | 0.0102 | 41.6205 | 41.3879 |
|  | 204->211 | 0.24848 |  |  |  |  |  |
|  | 204->213 | 0.14231 |  |  |  |  |  |
|  | 204->214 | -0.15712 |  |  |  |  |  |
| 35 | 198->208 | -0.14404 | 7.5867 | 163.42 | 0.0098 | -19.453 | -20.1019 |
|  | 202->208 | 0.16793 |  |  |  |  |  |
|  | 202->211 | -0.16028 |  |  |  |  |  |
| 36 | 180->206 | 0.15094 | 7.6256 | 162.59 | 0.0116 | 9.8658 | 9.8863 |
|  | 184->206 | 0.14608 |  |  |  |  |  |
|  | 188->206 | 0.25616 |  |  |  |  |  |
|  | 199->206 | 0.17042 |  |  |  |  |  |
|  | 199->207 | -0.14957 |  |  |  |  |  |

*^a^*Number of the excited states; *^b^*Only transitions with contribution over 4.0% were listed; *^c^*Configuration-interaction coefficient; *^d^*Excitation energy; *^e^*Wavelength; *^f^*Oscillator strength; *^g^*Rotatory strength in velocity form (10^-40^ cgs); *^h^*Rotatory strength in length form (10^-40^ cgs).

**9.4 Computational data of 4b**

**Table S10** Conformational analysis of the B3LYP-D3BJ/6-31G(d) optimized conformers of **4b** in the gas phase (T=298.15 K)

| Conformer | E (Hartree)*^a^* | C (Hartree)*^b^* | G (kcal/mol)*^c^* | *Δ*G (kcal/mol)*^d^* | Population*^e^* |
| --- | --- | --- | --- | --- | --- |
| **4b-1** | -2610.713652 | 0.836689 | -1637697.794339 | 0.0 | 61.54% |
| **4b-2** | -2610.712975 | 0.836455 | -1637697.516024 | 0.278315 | 38.46% |

*^a^*Electronic energy obtained at M06-2X-D3/6-311+G(2d,p) level of theory; *^b^*Thermal correction to Gibbs free energy obtained at B3LYP-D3BJ/6-31G(d) level of theory; *^c^*Gibbs free energy (E + C); *^d^*The relative Gibbs free energy; *^e^*The Boltzmann distribution of each conformer.

**Table S11** Atomic coordinates (Å) of **4b-1** obtained at the B3LYP-D3BJ/6-31G(d) level of theory in the gas phase.

| C | -5.270894 | 2.035214 | 0.052879 | O | 1.176754 | -2.307526 | 1.454549 |
| --- | --- | --- | --- | --- | --- | --- | --- |
| C | -6.395281 | 3.050331 | -0.012378 | H | 5.035320 | -4.047267 | -0.976494 |
| C | -7.539380 | 2.587847 | 0.889784 | H | -5.743716 | -1.999116 | 1.510814 |
| C | -8.161073 | 1.258851 | 0.414451 | H | -6.786418 | -2.844360 | -0.756009 |
| C | -7.043933 | 0.163118 | 0.386024 | H | 6.400968 | 1.682228 | -0.988608 |
| C | -7.408240 | -1.012688 | -0.519664 | O | 8.055710 | 2.004653 | 0.191409 |
| C | -3.214646 | 1.618282 | -1.113296 | H | 1.596015 | -0.270259 | -3.404043 |
| C | -3.112614 | 0.378291 | -0.243135 | H | 1.537088 | -1.860548 | -2.674983 |
| C | -4.461439 | -0.296905 | 0.249076 | H | 5.691565 | 2.557461 | 1.240471 |
| C | -5.665685 | 0.580490 | -0.219449 | H | 6.231695 | 1.059078 | 2.006075 |
| C | -4.337529 | -0.651011 | 1.750603 | H | 1.857069 | 1.445267 | -0.972614 |
| C | -2.999356 | -1.373824 | 2.043052 | H | 0.524620 | 1.186730 | 0.146592 |
| C | -1.758567 | -0.731141 | 1.369237 | H | 8.322659 | -1.366693 | -1.751042 |
| C | -2.151824 | 0.688074 | 0.932253 | H | 9.071625 | 0.179987 | -1.323791 |
| C | -2.367997 | -0.756788 | -0.966227 | H | 7.658202 | 0.144142 | -2.394147 |
| C | -1.417016 | -1.418544 | 0.028273 | H | -2.872658 | -1.398709 | 3.129464 |
| C | -1.412138 | -2.962100 | -0.122911 | H | -3.115788 | -2.417084 | 1.728309 |
| C | -9.218069 | 0.814856 | 1.442223 | H | 5.328320 | -1.127833 | 2.289390 |
| C | -8.880794 | 1.487725 | -0.932511 | H | 3.848701 | -1.979923 | 1.820268 |
| C | -5.895741 | 0.300248 | -1.726868 | H | -2.601096 | 1.283222 | 1.730042 |
| C | 4.532461 | 0.845066 | 0.687025 | H | -1.300722 | 1.251182 | 0.547106 |
| C | 5.848766 | 1.488252 | 1.076493 | H | -7.162938 | 2.462865 | 1.915600 |
| C | 6.833865 | 1.276620 | -0.067511 | H | -8.319451 | 3.356880 | 0.936464 |
| C | 7.194024 | -0.204816 | -0.283108 | H | -4.377768 | 0.271744 | 2.341912 |
| C | 5.862235 | -0.960135 | -0.617411 | H | -6.009321 | 4.024974 | 0.304657 |
| C | 5.974735 | -2.467537 | -0.381459 | H | -6.730808 | 3.167235 | -1.047323 |
| C | 2.416825 | 0.381029 | 1.706643 | H | 3.448155 | 0.438926 | -2.092734 |
| C | 2.078228 | -0.299233 | 0.392888 | H | 6.990307 | -2.864345 | -0.486243 |
| C | 3.261629 | -0.957260 | -0.433448 | H | -0.906250 | -0.732333 | 2.050612 |
| C | 4.617243 | -0.620836 | 0.256123 | H | -4.822117 | 2.094070 | 1.053570 |
| C | 3.132449 | -0.598103 | -1.921111 | H | -0.538607 | -2.816127 | -2.128982 |
| C | 1.694237 | -0.799997 | -2.450793 | H | 0.587040 | -3.587430 | -0.965711 |
| C | 0.595312 | -0.298933 | -1.470650 | H | 4.108500 | 1.430042 | -0.139655 |
| C | 1.255028 | 0.676393 | -0.483048 | H | -8.482670 | -1.197431 | -0.610890 |
| C | 1.089936 | -1.456705 | 0.595485 | H | -1.133415 | -3.440967 | 0.817558 |
| C | 0.078827 | -1.428806 | -0.549933 | H | -2.327577 | -3.408253 | -0.520144 |
| C | -0.214702 | -2.854551 | -1.087147 | H | 8.817315 | -0.106774 | 1.143010 |
| C | 8.116287 | -0.317930 | -1.511645 | H | 7.352309 | -0.798061 | 1.847396 |
| C | 7.958167 | -0.751804 | 0.941782 | H | 8.335323 | -1.759855 | 0.753407 |
| C | 4.801649 | -1.590656 | 1.448851 | H | -0.219789 | 0.168417 | -2.025684 |
| C | 8.071050 | 3.312667 | -0.165825 | H | -5.002411 | -0.084015 | -2.228279 |
| C | 9.407343 | 3.933079 | 0.157988 | H | -6.233888 | 1.188100 | -2.269198 |
| O | -6.927259 | -0.701163 | -1.801061 | H | -9.604804 | 2.302643 | -0.816870 |
| O | 5.590916 | -2.686949 | 0.957445 | H | -8.207174 | 1.757557 | -1.747299 |
| O | -6.795284 | -2.209472 | -0.021185 | H | -9.436842 | 0.602655 | -1.253172 |
| H | 3.809749 | -1.232821 | -2.496365 | H | -9.663022 | -0.149090 | 1.169271 |
| O | -5.403781 | -1.449826 | 2.243391 | H | -8.778975 | 0.709321 | 2.440605 |
| O | -4.266810 | 2.442192 | -0.909935 | H | -10.029858 | 1.548977 | 1.503045 |
| H | -4.557149 | -1.253142 | -0.273428 | O | -2.349229 | 1.929426 | -1.899116 |
| H | 3.148496 | -2.041604 | -0.371889 | O | 1.617156 | 0.493161 | 2.606173 |
| H | -6.906845 | -0.183944 | 1.409234 | O | 7.131160 | 3.883959 | -0.672801 |
| H | 5.650434 | -0.770425 | -1.670232 | H | 10.207999 | 3.392076 | -0.356447 |
| O | 5.117881 | -3.133380 | -1.294691 | H | 9.406487 | 4.979229 | -0.148491 |
| O | 3.637754 | 0.959534 | 1.816916 | H | 9.601739 | 3.859463 | 1.232953 |
| O | -2.591956 | -1.124960 | -2.099430 | - | - | - | - |

**Table S12** Atomic coordinates (Å) of **4b-2** obtained at the B3LYP-D3BJ/6-31G(d) level of theory in the gas phase.

| C | -5.308356 | 2.027758 | 0.196009 | O | 1.142299 | -2.389303 | 1.314318 |
| --- | --- | --- | --- | --- | --- | --- | --- |
| C | -6.436771 | 3.040633 | 0.201194 | H | 5.042933 | -3.913209 | -1.204120 |
| C | -7.589054 | 2.501001 | 1.048439 | H | -5.778908 | -2.113819 | 1.312366 |
| C | -8.198680 | 1.213199 | 0.458108 | H | -6.792885 | -2.775607 | -1.027950 |
| C | -7.076099 | 0.127785 | 0.353424 | H | 6.380288 | 1.802210 | -0.736531 |
| C | -7.424247 | -0.970930 | -0.649992 | O | 7.982936 | 2.073041 | 0.523148 |
| C | -3.236288 | 1.716335 | -0.975308 | H | 1.611124 | 0.040579 | -3.356341 |
| C | -3.138671 | 0.409101 | -0.209480 | H | 1.550434 | -1.604485 | -2.761604 |
| C | -4.489893 | -0.309424 | 0.209675 | H | 5.641018 | 2.489309 | 1.566507 |
| C | -5.692890 | 0.598777 | -0.199558 | H | 6.176137 | 0.935497 | 2.202696 |
| C | -4.381446 | -0.785230 | 1.678420 | H | 1.834432 | 1.551145 | -0.790606 |
| C | -3.043522 | -1.525076 | 1.925779 | H | 0.489906 | 1.197398 | 0.287808 |
| C | -1.798065 | -0.824359 | 1.322030 | H | 7.688635 | 0.422594 | -2.227988 |
| C | -2.192741 | 0.624471 | 0.998813 | H | 8.290360 | -1.171273 | -1.730385 |
| C | -2.380283 | -0.659774 | -1.014650 | H | 9.079973 | 0.294061 | -1.140060 |
| C | -1.437591 | -1.397584 | -0.066870 | H | -2.929158 | -1.639476 | 3.007800 |
| C | -1.423286 | -2.923409 | -0.344307 | H | -3.151594 | -2.539021 | 1.524292 |
| C | -9.265377 | 0.682438 | 1.433410 | H | 5.275218 | -1.268876 | 2.294322 |
| C | -8.903841 | 1.549265 | -0.874010 | H | 3.808319 | -2.087216 | 1.734770 |
| C | -5.904614 | 0.442433 | -1.727468 | H | -2.653631 | 1.150388 | 1.837548 |
| C | 4.493601 | 0.825197 | 0.849263 | H | -1.339896 | 1.220325 | 0.671076 |
| C | 5.803078 | 1.438671 | 1.306805 | H | -7.224218 | 2.293484 | 2.065022 |
| C | 6.797615 | 1.330975 | 0.158644 | H | -8.373352 | 3.260562 | 1.148639 |
| C | 7.169668 | -0.129746 | -0.171586 | H | -4.432457 | 0.085594 | 2.343097 |
| C | 5.846712 | -0.860918 | -0.584084 | H | -6.059017 | 3.987197 | 0.602105 |
| C | 5.964401 | -2.381947 | -0.470040 | H | -6.760949 | 3.241316 | -0.824479 |
| C | 2.367000 | 0.273276 | 1.799960 | H | 3.444935 | 0.645493 | -1.969281 |
| C | 2.047598 | -0.298205 | 0.430906 | H | 6.983136 | -2.764500 | -0.594845 |
| C | 3.243861 | -0.882260 | -0.432038 | H | -0.953762 | -0.878682 | 2.011159 |
| C | 4.589882 | -0.599600 | 0.299295 | H | -4.871711 | 2.005741 | 1.203499 |
| C | 3.131004 | -0.402891 | -1.886738 | H | -0.526583 | -2.609759 | -2.320722 |
| C | 1.700174 | -0.565530 | -2.448805 | H | 0.588850 | -3.469533 | -1.210963 |
| C | 0.587076 | -0.150859 | -1.444614 | H | 4.077004 | 1.473786 | 0.067251 |
| C | 1.230185 | 0.742836 | -0.372386 | H | -8.496618 | -1.152366 | -0.768319 |
| C | 1.062265 | -1.472005 | 0.525956 | H | -1.153463 | -3.476805 | 0.557045 |
| C | 0.065042 | -1.354268 | -0.625778 | H | -2.331744 | -3.338992 | -0.787981 |
| C | -0.215007 | -2.732344 | -1.281643 | H | 8.319360 | -1.749718 | 0.748552 |
| C | 8.109822 | -0.145265 | -1.391935 | H | 8.762232 | -0.128666 | 1.290256 |
| C | 7.919190 | -0.768781 | 1.016722 | H | 7.298824 | -0.905080 | 1.903137 |
| C | 4.763926 | -1.663661 | 1.410618 | H | -0.223549 | 0.357196 | -1.969613 |
| C | 8.422181 | 3.025620 | -0.339648 | H | -5.004007 | 0.103348 | -2.248039 |
| C | 9.669336 | 3.680315 | 0.200286 | H | -6.239930 | 1.370783 | -2.199043 |
| O | -6.931263 | -0.552441 | -1.895865 | H | -8.222241 | 1.889044 | -1.655388 |
| O | 5.567360 | -2.711061 | 0.842568 | H | -9.450865 | 0.691147 | -1.273663 |
| O | -6.810180 | -2.201732 | -0.244722 | H | -9.633854 | 2.348299 | -0.700028 |
| H | 3.817926 | -0.985941 | -2.503663 | H | -10.081180 | 1.406114 | 1.544588 |
| O | -5.449708 | -1.625511 | 2.091514 | H | -9.702714 | -0.257465 | 1.077136 |
| O | -4.294857 | 2.516565 | -0.717759 | H | -8.837319 | 0.496978 | 2.424912 |
| H | -4.574700 | -1.220022 | -0.390437 | O | -2.362878 | 2.094831 | -1.721782 |
| H | 3.134772 | -1.968392 | -0.460904 | O | 1.555995 | 0.310124 | 2.695454 |
| H | -6.949313 | -0.301469 | 1.346322 | O | 7.883824 | 3.297244 | -1.389138 |
| H | 5.647286 | -0.585963 | -1.620390 | H | 9.985804 | 4.474844 | -0.475666 |
| O | 5.120593 | -2.975161 | -1.443374 | H | 9.479730 | 4.086949 | 1.198461 |
| O | 3.584627 | 0.844378 | 1.972941 | H | 10.466587 | 2.935851 | 0.297529 |
| O | -2.589620 | -0.934349 | -2.176778 | - | - | - | - |

1. **Information of known compounds**

**Fig. S44** Chemical structures of known diterpenoids (**5**−**41**) isolated from *Isodon silvaticus*.

**Table S13** Compound names and references of known diterpenoids (**5**−**41**) isolated from *Isodon silvaticus*.

| **No.** | **Compound name** | **Reference** |
| --- | --- | --- |
| **5** | effusanin E | Fujita T, Takeda Y, Shingu T and Ueno A. Structures of effusanins, antibacterial diterpenoids from *Rabdosia effusa*. Chem Lett*.* 2006;9(12):1635–8. |
| **6** | nervosanin B | Feng C, Guo LQ, Yan FL, Cui JM and Di XM. 1α,6β,7β,11α,15β-Penta-hydr-oxy-7α,20-ep-oxy-ent-kaur-16-ene. Acta Crystallogr Sect E Struct Rep Online*.* 2010;66(Pt 2):o334. |
| **7** | taibaihenryiin A | Li BL, Pan YJ and Pan WJ. Two New C(20)-Oxygenated *ent*-Kaurene Diterpenoids from *Isodon henryi*. Helvetica Chimica Acta*.* 2001;84(11):3418–22. |
| **8** | effusanin C | Fujita T, Takeda Y, Shingu T and Ueno A. Structures of effusanins, antibacterial diterpenoids from *Rabdosia effusa*. Chem Lett*.* 2006;9(12):1635–8. |
| **9** | longikaurin D | Fujita T, Takeda Y and Shingu T. Longikaurin C, D, E and F; New Antibacterial Diterpenoids from *Rabdosia longituba*. Heterocycles*.* 1981;16:227. |
| **10** | wikstroemioidin A | Wu SH, Zang HJ, Chen YP, Lin ZW and Sun HD. Diterpenoids from *Isodon wikstroemioides*. Phytochemistry*.* 1993;34(4):1099–102. |
| **11** | oreskaurin B | Xiang W, Li RT, Wang ZY, Li SH, Zhao QS, Zhang HJ, et al. *ent*-Kaurene diterpenoids from *Isodon oresbius*. Phytochemistry*.* 2004;65(8):1173–7. |
| **12** | longikaurin A | Fujita T, Takeda Y and Shingu T. Longikaurin A and B; new, biologically active diterpenoids from *Rabdosia longituba*. J Chem Soc Chem Commun*.* 1980;(5):205–7. |
| **13** | phyllostacin C | Sun HD, Li X, Pu JX, Li SH, Huang SX, Weng ZY, et al. Three new *ent*-kauranoids from *Isodon phyllostachys*. Heterocycles*.* 2007;71(11):2441. |
| **14** | enmenol | Mori S, Shudo K, Ageta T, Koizumi T and Okamoto T. Studies on the Constituents of *Isodon trichocarpus* KUDO. I. Isolation of the Constituents and the Structures of Isodonol, Enmedol, and Enmenol. Chem Pharm Bull*.* 1970;18(5):871–83. |
| **15** | oridonin | Fujita E, Fujita T, Katayama H, Shibuya M and Shingu T. Terpenoids. Part XV. Structure and absolute configuration of oridonin isolated from *Isodon japonicus* and *Isodon trichocarpus*. J Chem Soc C*.* 1970;(12):1674–81. |
| **16** | rubluanin D | Zhang HB, Pu JX, Wang YY, He F, Zhao Y, Li XN, et al. Four New *ent*-Kauranoids from *Isodon rubescens* var. lushanensis and Data Reassignment of Dayecrystal B. Chem Pharm Bull*.* 2010;58(1):56–60. |
| **17** | dayecrystal B | Zhang HB, Pu JX, Wang YY, He F, Zhao Y, Li XN, et al. Four New *ent*-Kauranoids from *Isodon rubescens* var. lushanensis and Data Reassignment of Dayecrystal B. Chem Pharm Bull*.* 2010;58(1):56–60. |
| **18** | lasiokaurin | Fujita E and Taoka M. Terpenoids. XX. The Structure and Absolute Configuration of Lasiokaurin and Lasiodonin, New Diterpenoids from *Isodon lasiocarpus* (HAYATA) KUDO. Chem Pharm Bull*.* 1972;20:1752–4. |
| **19** | isojiangrubesin D | Zhang YY, Jiang HY, Liu M, Hu K, Wang WG, Du X, et al. Bioactive *ent*-kaurane diterpenoids from *Isodon rubescens*. Phytochemistry*.* 2017;143:199–207. |
| **20** | 6*β*,7*β*,11*α*-Trihydroxy-7*α*,20-epoxy-*ent*-kaur-16-en-15-one | Fujita, E.; Fuji, K.; Noide, M.; Takashima, J.; Furui, M.; Abe, T.; Egawa, M. JP07173147A, 1995. |
| **21** | excisanin C | Chang R, Kim D, U Z, Xiaoyu S, Yiping C and Handong S. A diterpenoid from *Rabdosia excisa*. Phytochemistry*.* 1992;31(1):342–3. |
| **22** | excisanin J | Gui MY, Aoyagi Y, Jin Y, Li XW, Hasuda T and Takeya K. Excisanin H, a novel cytotoxic 14,20-epoxy-ent-kaurene diterpenoid, and three new *ent*-kaurene diterpenoids from *Rabdosia excisa*. J Nat Prod*.* 2004;(673):373–6. |
| **23** | isorosthornin G | Zhan R, Li XN, Du X, Wang WG, Dong K, Su J, et al. *ent*-Atisane and *ent*-kaurane diterpenoids from *Isodon rosthornii*. Fitoterapia*.* 2013;(88):76–81. |
| **24** | sculponeatin F | Jiang B, Hou AJ, Li ML, Li SH, Han QB, Wang SJ, et al. Cytotoxic *ent*-kaurane diterpenoids from *Isodon sculponeata*. Planta Med*.* 2002;68(10):921–5. |
| **25** | nodosin | Fujita E, Fujita T and Shibuya M. Terpenoids. VII. The Structure and Absolute Configuration of nodosin, a New Diterpenoid from *Isodon* Species. Chem Pharm Bull*.* 1968;16(3):509–15. |
| **26** | epinodosinol | Fujita E, Fujita T, Taoka M, Katayama H and Shibuya M. The structure and absolute configuration of sodoponin and epinodosinol, new minor diterpenoids of *Isodon japonicus*. Tetrahedron Lett*.* 1970;11(6):421–4. |
| **27** | longirabdolide C | Takeda Y, Matsumoto T and Otsuka H. Longirabdolide C, a new diterpenoid from *Rabdosia longituba*. J Nat Prod*.* 1994;57(5):650–3. |
| **28** | carpalasionin | Zhang YY, Jiang HY, Liu M, Hu K, Wang WG, Du X, et al. Bioactive *ent*-kaurane diterpenoids from *Isodon rubescens*. Phytochemistry*.* 2017;143:199–207. |
| **29** | dihydrorugosanin | Fang L and Xu XJ. Studies on the chemical constituents of *Rabdosia rubescens*. J Chin Med Mater*.* 2008;31(9):1340–3. |
| **30** | sculponeatin A | Sun H, Lin Z, Xu Y, Minami Y, Marunaka T, Togo T, et al. structures of sculponeatin A, sculponeatin B, and sculponeatin C, 3 new diterpenoids having unique acetal structures from *Rabdosia sculponeata*. Heterocycles*.* 1986;24(1):1–4. |
| **31** | 15*β*-hydroxy-6,7-seco-6,11*β*:6,20-diepoxy-1*α*,7-olide-*ent*-kaur-16-one | Yan FL, Guo LQ, Zhang JX, Bai SP and Sun HD. New *ent*-kaurane-type diterpenoids from *Isodon nervosus*. Chin Chem Lett*.* 2008;19(4):441–3. |
| **32** | enmein-type diterpenoid analogs **11** | Li DH, Xu ST, Cai H, Pei LL, Zhang HY, Wang L, et al. Enmein-type diterpenoid analogs from natural kaurene-type oridonin: Synthesis and their antitumor biological evaluation. Eur J Med Chem*.* 2013;64:215–21. |
| **33** | enmein-type diterpenoid analogs **10** | Li DH, Xu ST, Cai H, Pei LL, Zhang HY, Wang L, et al. Enmein-type diterpenoid analogs from natural kaurene-type oridonin: Synthesis and their antitumor biological evaluation. Eur J Med Chem*.* 2013;64:215–21. |
| **34** | *δ*-lactone hemiacetal | Mori S, Shudo K, Ageta T, Koizumi T and Okamoto T. Studies on the constituents of *Isodon trichocarpus* KUDO. *I*. Isolation of the constituents and the structures of isodonol, enmedol, and enmenol. Chem Pharm Bull*.* 1970;18(5):871–83. |
| **35** | enmein-3-acetate | 藤田 栄, 藤多 哲 and 渋谷 雅. Terpenoids(第6報) : ヒキオコシ *Isodon japonicus* Hara から Enmein および Enmein 3-acetate の分離について. 藥學雜誌*.* 1967;87(9):1076–8. |
| **36** | dihydroisodocarpin | Fang L and Xu XJ. Studies on the chemical constituents of *Rabdosia rubescens*. J Chin Med Mater*.* 2008;31(9):1340–3. |
| **37** | isodocarpin | Fujita E, Fujita T and Shibuya M. Terpenoids. IX. The structure and absolute configuration of isodocarpin, a New Diterpenoid from *Isodon trichocarpus* KUDO and *I. japonicus* Hara. Chem Pharm Bull*.* 1968;16(8):1573–5. |
| **38** | dihydroenmein | 池田 鉄 and 金友 昭. クロバナヒキオコシ成分の研究 (第1報). 藥學雜誌*.* 1958;78(10):1128–32.  Fujita E, Fujita T and Shibuya M. Diterpenoid constituents of *Isodon trichocarpus* and *Isodon japonicus* (terpenoids IV). Tetrahedron Lett*.* 1966;7(27):3153–62. |
| **39** | enmein | 池田 鉄 and 金友 昭. クロバナヒキオコシ成分の研究 (第1報). 藥學雜誌*.* 1958;78(10):1128–32.  Fujita E, Fujita T and Shibuya M. Diterpenoid constituents of *Isodon trichocarpus* and *Isodon japonicus* (terpenoids IV). Tetrahedron Lett*.* 1966;7(27):3153–62. |
| **40** | bisjaponin B | Yang LB, Yang J, Li LM, Lei C, Zhao Y, Huang SX, et al. Symmetric and asymmetric *ent*-kaurane dimers isolated from *Isodon japonicus*. Tetrahedron Lett*.* 2008;49(22):3574–7. |
| **41** | lushanrubescensin J | Han QB, Lu Y, Wu L, He ZD, Qiao CF, Xu HX, et al. An asymmetric *ent*-kauranoid dimer from *Isodon rubescens* var. lushanensis. Tetrahedron Lett*.* 2005;46(32):5373–5. |
